# Crystal structures of seven mixed-valence gold compounds of the form [(*R*^1^*R*^2^*R*^3^P*E*)_2_Au^I^]^+^[Au^III^*X*_4_]^−^ (*R* = *tert*-butyl or isopropyl, *E* = S or Se, and *X* = Cl or Br)

**DOI:** 10.1107/S2056989024009095

**Published:** 2024-09-30

**Authors:** Daniel Upmann, Dirk Bockfeld, Peter G. Jones, Eliza Târcoveanu

**Affiliations:** a Institut für Anorganische und Analytische Chemie, Technische Universität Braunschweig, Hagenring 30, D-38106 Braunschweig, Germany; Vienna University of Technology, Austria

**Keywords:** crystal structure, gold, phosphane chalcogenides, secondary inter­actions

## Abstract

During our studies of the oxidation of gold(I) complexes of tri­alkyl­phosphane chalcogenides, general formula *R*^1^*R*^2^*R*^3^P*E*Au*X*, (*R* = *tert*-butyl or isopropyl, *E* = S or Se, *X* = Cl or Br) with PhICl_2_ or elemental bromine, we have isolated a set of seven mixed-valence by-products, the bis­(tri­alkyl­phosphane chalcogenido)gold(I) tetra­halogenidoaurates(III) [(*R*^1^*R*^2^*R*^3^P*E*)_2_Au]^+^[Au*X*_4_]^−^. These corres­pond to the addition of one halogen atom per gold atom of the Au^I^ precursor. Com­pound **1**, bis­(triiso­propyl­phosphane sulfide)­gold(I) tetra­chlorido­aur­ate(III), [Au(C_9_H_21_PS)_2_][AuCl_4_] or [(^*i*^Pr_3_PS)_2_Au][AuCl_4_], crystallizes in space group *P*2_1_/*n* with *Z* = 4; the gold(I) atoms of the two cations lie on twofold rotation axes, and the gold(III) atoms of the two anions lie on inversion centres. Compound **2**, bis­(*tert*-butyl­diiso­propyl­phosphane sulfide)­gold(I) tetra­chlorido­aurate(III), [Au(C_10_H_23_PS)_2_][AuCl_4_] or [(^*t*^Bu^*i*^Pr_2_PS)_2_Au][AuCl_4_], crystallizes in space group *P*1 with *Z* = 4; the asymmetric unit contains two cations and two anions with no imposed symmetry. A least-squares fit of the two cations gave an r.m.s. deviation of 0.19 Å. Compound **3**, bis­(tri-*tert*-butyl­phosphane sulfide)­gold(I) tetra­chlorido­aurate(III), [Au(C_12_H_27_PS)_2_][AuCl_4_] or [(^*t*^Bu_3_PS)_2_Au][AuCl_4_], crystallizes in space group *P*1 with *Z* = 1; both gold atoms lie on inversion centres. Compound **4a**, bis­(*tert*-butyl­diiso­propyl­phosphane sulfide)­gold(I) tetra­bromi­doaurate(III), [Au(C_10_H_23_PS)_2_][AuBr_4_] or [(^*t*^Bu^*i*^Pr_2_PS)_2_Au][AuBr_4_], crystallizes in space group *P*2_1_/*c* with *Z* = 4; the cation lies on a general position, whereas the gold(III) atoms of the two anions lie on inversion centres. Compound **4b**, bis­(*tert*-butyl­diiso­propyl­phosphane selenide)gold(I) tetra­bromido­aurate(III), [Au(C_10_H_23_PSe)_2_][AuBr_4_] or [(^*t*^Bu^*i*^Pr_2_PSe)_2_Au][AuBr_4_], is isotypic with **4a**. Compound **5a**, bis­(tri-*tert*-butyl­phosphane sulfide)­gold(I) tetra­bromido­aurate(III), [Au(C_12_H_27_PS)_2_][AuBr_4_] or [(^*t*^Bu_3_PS)_2_Au][AuBr_4_], is isotypic with compound **4a**. Compound **5a**, bis­(tri-*tert*-butyl­phosphane sulfide)­gold(I) tetra­bromido­aurate(III), [Au(C_12_H_27_PS)_2_][AuBr_4_] or [(^*t*^Bu_3_PS)_2_Au][AuBr_4_], crystallizes in space group *P*1 with *Z* = 1; both gold atoms lie on inversion centres. Compound **5b**, bis­(tri-*tert*-butyl­phosphane selenide)gold(I) tetra­bromido­aurate(III), [Au(C_12_H_27_PSe)_2_][AuBr_4_] or [(^*t*^Bu_3_PSe)_2_Au][AuBr_4_], is isotypic with **5a**. All Au^I^ atoms are linearly coordinated and all Au^III^ atoms exhibit a square-planar coordination environment. The ligands at the Au^I^ atoms are anti­periplanar to each other across the S⋯S vectors. There are several short intra­molecular H⋯Au and H⋯*E* contacts. Average bond lengths (Å) are: P—S = 2.0322, P—Se = 2.1933, S—Au = 2.2915, and Se—Au = 2.4037. The complex three-dimensional packing of **1** involves two short C—H_methine_⋯Cl contacts (and some slightly longer contacts). For **2**, four C—H_methine_⋯Cl inter­actions combine to produce zigzag chains of residues parallel to the *c* axis. Additionally, an S⋯Cl contact is observed that might qualify as a ‘chalcogen bond’. The packing of **3** is three-dimensional, but can be broken down into two layer structures, each involving an S⋯Cl and an H⋯Cl contact. For the bromido derivatives **4a**/**b** and **5a**/**b**, loose associations of the anions form part of the packing patterns. For all four compounds, these combine with an *E*⋯Br contact to form layers parallel to the *ab* plane.

## Abstract 2

The ligands at the Au^I^ atoms are anti­periplanar to each other across the S⋯S vectors. Residues are linked by various contacts of the types C—H⋯*X*, *E*⋯*X* and Br⋯Br.

## 1. Chemical context

In the three previous publications in this series, we have presented structures of tri­alkyl­phosphane chalcogenido complexes of gold(I), general formula [(*R*^1^*R*^2^*R*^3^P*E*)Au*X*] (Upmann *et al.*, 2024*a* ▸), the corresponding trihalogenido-gold(III) complexes [(*R*^1^*R*^2^*R*^3^P*E*)Au*X*_3_] (Upmann *et al.*, 2024*b* ▸) and the further oxidized phospho­nium gold(III) deriv­atives (*R*^1^*R*^2^*R*^3^P*EX*)^+^[Au*X*_4_]^−^ (Upmann *et al.*, 2024*c* ▸), where the *R* groups are *tert*-butyl or isopropyl, the chalcogen atoms *E* are S or Se, and the halogen atoms *X* are Cl or Br [the iodido­gold(I) derivatives however cannot be oxidized to gold(III)]. The majority of the gold(III) derivatives were synthesized successfully by the oxidation of the Au^I^ series with PhICl_2_ or elemental bromine, whereby the two oxidation steps each correspond to the addition of two atom equivalents of halogen per atom equivalent of gold. However, some failed syntheses and several syntheses with low yields led us to suspect that the systems in solution were in some cases complex mixtures. One further set of isolated products were the mixed-valence bis(trialkylphosphane chalcogenido)gold(I) tetrahalogenidoaurates(III) of the form [(*R*^1^*R*^2^*R*^3^P*E*)_2_Au]^+^[Au*X*_4_]^−^, and the structures of seven such compounds (Scheme, Table 1 ▸) are presented here; they correspond to the addition of one halogen atom per gold atom of the Au^I^ precursor, rather than the two or four halogen atoms added to produce [(*R*^1^*R*^2^*R*^3^P*E*)Au*X*_3_] or (*R*^1^*R*^2^*R*^3^P*EX*)^+^[Au*X*_4_]^−^ respectively.
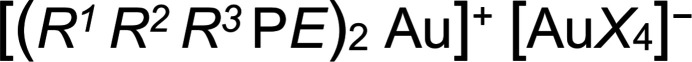


Much introductory material is given in Part 6 of this series (Upmann *et al.*, 2024*a* ▸) and is not repeated here. It is however worth repeating that writing a formal double bond P=*E* in the formulae of phosphane chalcogenides is an old-fashioned convention that probably does not represent the true nature of the bond. A considerable admixture of the resonance form ^+^P—*E*^−^ is likely to be involved, especially for metal complexes.

## 2. Structural commentary

**Figure 1:**
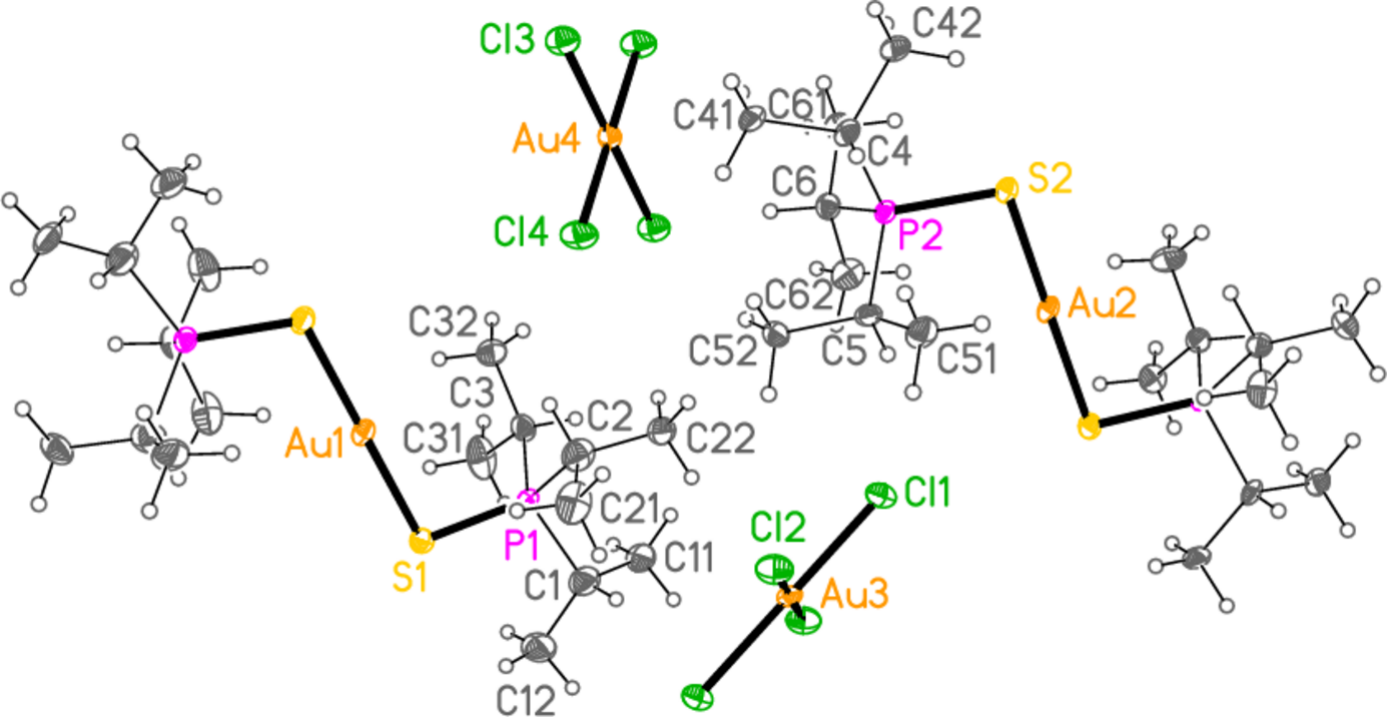
The structure of compound **1** in the crystal. Only the asymmetric unit is labelled.

The mol­ecular structures of compounds **1**–**5b** are shown in Figs. 1 ▸–7 ▸ ▸ ▸ ▸ ▸ ▸; some short inter­ionic contacts are included in these Figures and are discussed in section 3, *Supra­molecular features*. Ellipsoid plots correspond to 50% probability levels except for **4a** (30%). All the structures are solvent-free. Because some alkyl groups are overlapped, not all carbon atoms are labelled. Selected bond lengths and angles are presented in Tables 2 ▸–8 ▸ ▸ ▸ ▸ ▸ ▸. All Au^I^ atoms are linearly coordin­ated and all Au^III^ atoms are in a square-planar coordination environment (the anions have the ideal 4/*mmm* symmetry to a close approximation). For each phosphane chalcogenido ligand, there is a carbon atom that has an absolute torsion angle C—P—S—Au close to 180°; this is given the lowest number (C1 or C4) of the three carbon atoms bonded to the phospho­rus atom. This position is always occupied by a *tert*-butyl group, if present.

**Figure 2:**
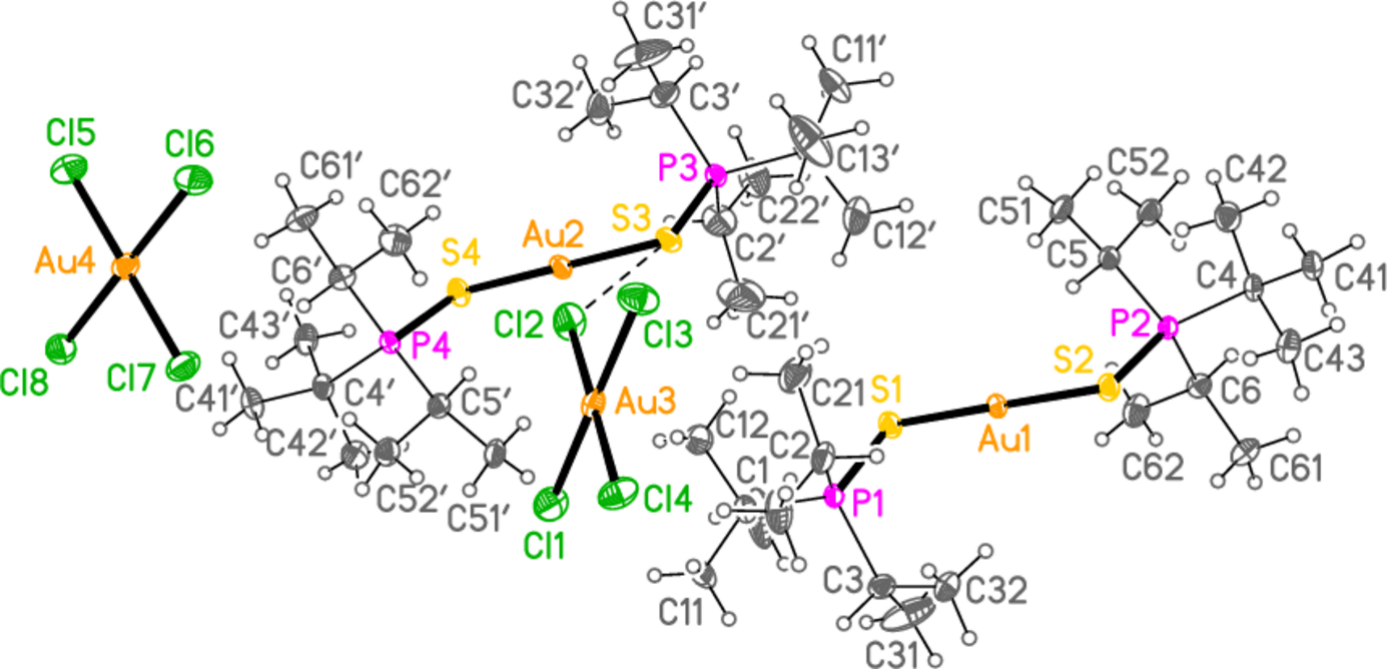
The structure of compound **2** in the crystal. Carbon atoms of the second independent cation are labelled with primes. The contact S3⋯Cl2 is indicated by a dashed bond.

**Figure 3:**
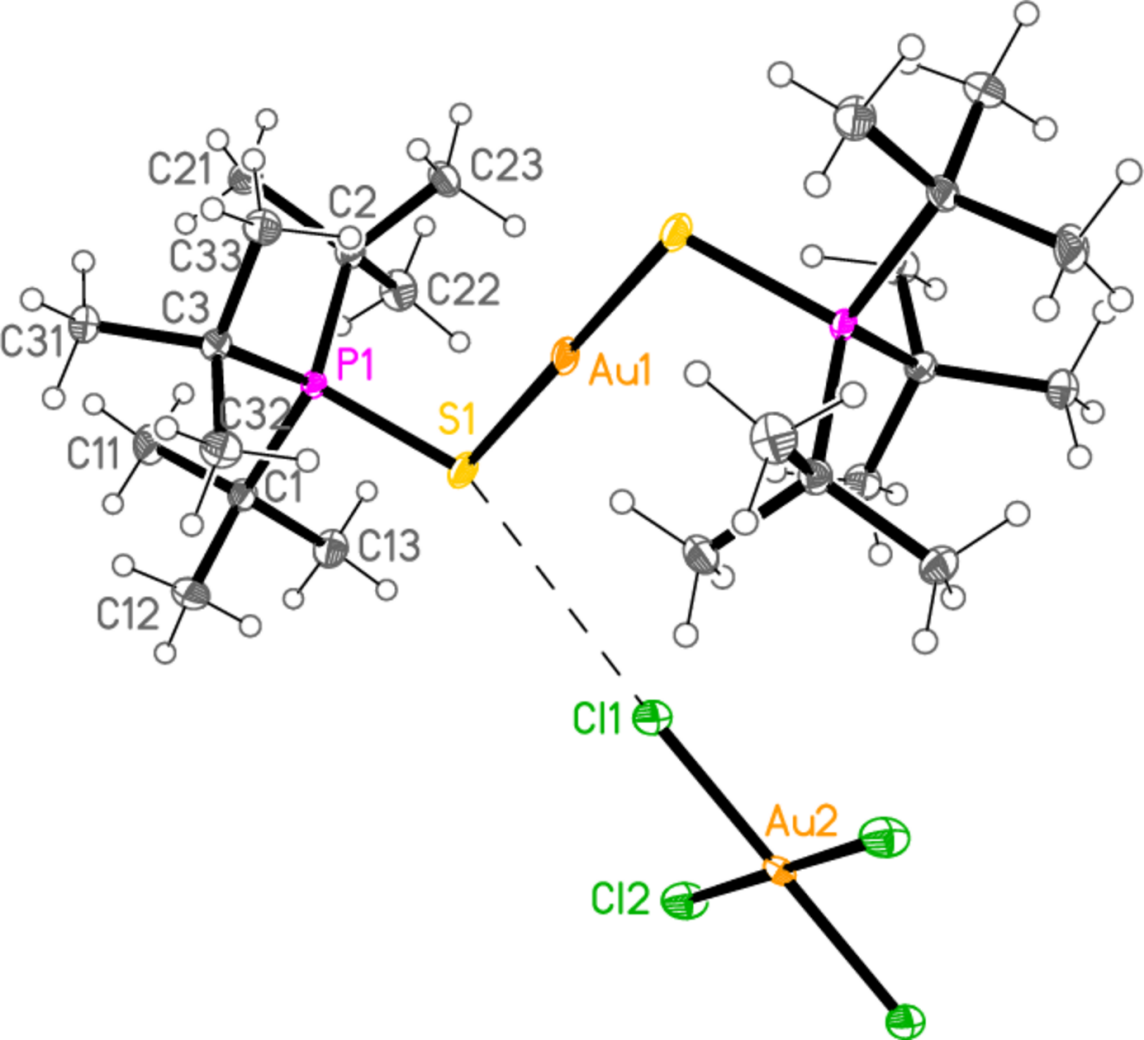
The structure of compound **3** in the crystal. Only the asymmetric unit is labelled. The contact S1⋯Cl1 is indicated by a dashed bond.

**Figure 4:**
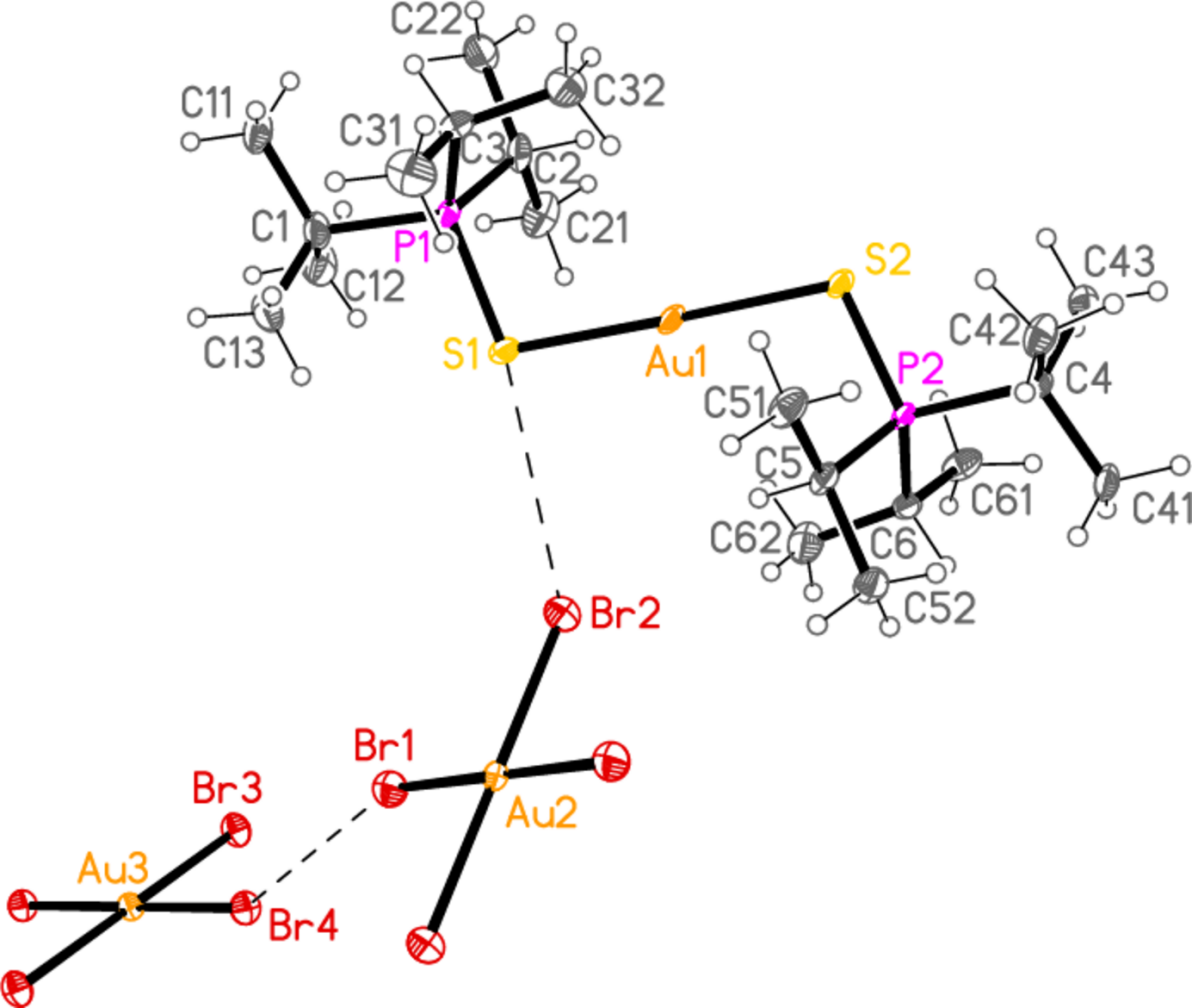
The structure of compound **4a** in the crystal. Only the asymmetric unit is labelled. The contacts S1⋯Br2 and Br1⋯Br4 are indicated by dashed bonds.

**Figure 5:**
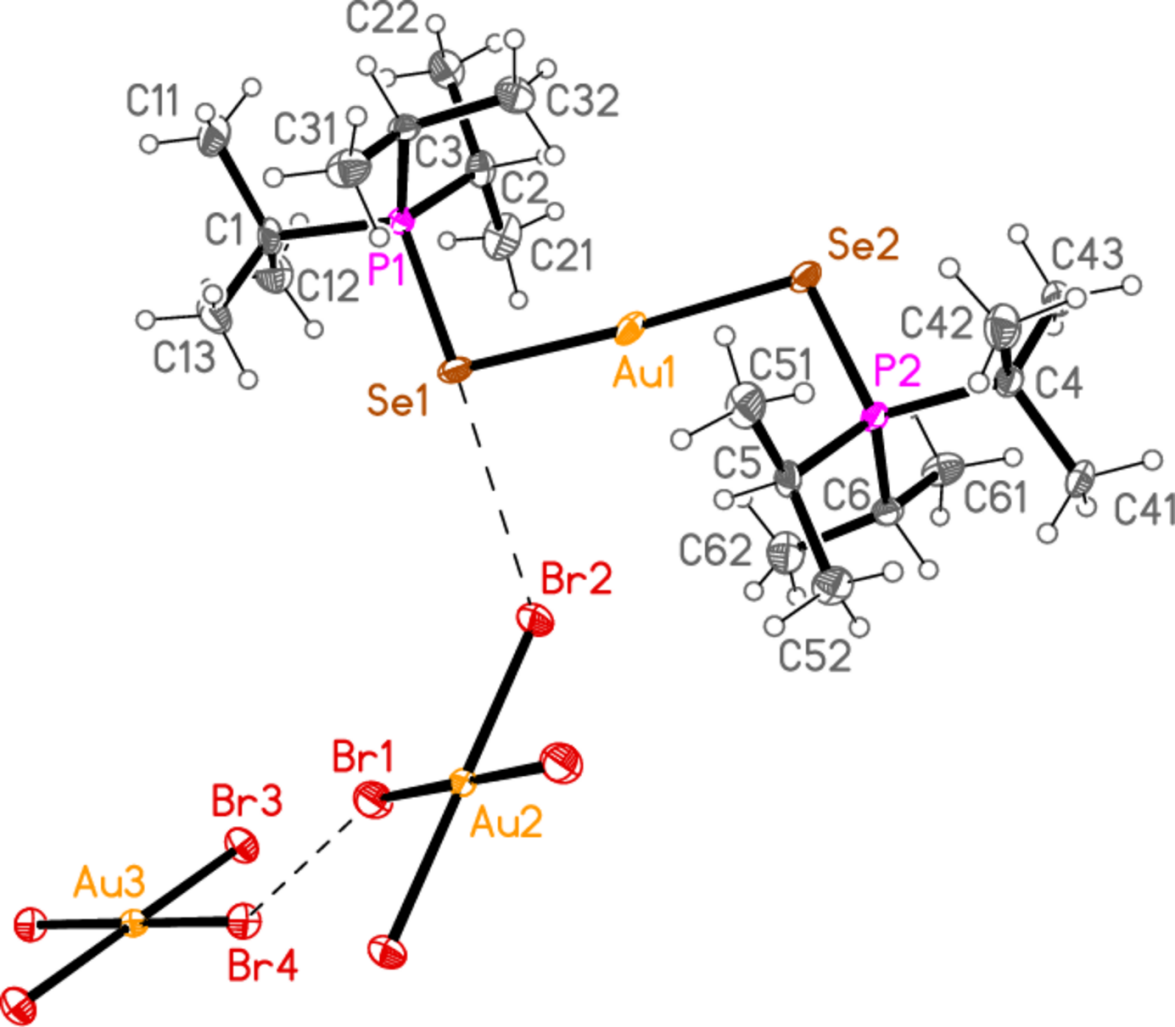
The structure of compound **4b** in the crystal. Only the asymmetric unit is labelled. The contacts Se1⋯Br2 and Br1⋯Br4 are indicated by dashed bonds.

**Figure 6:**
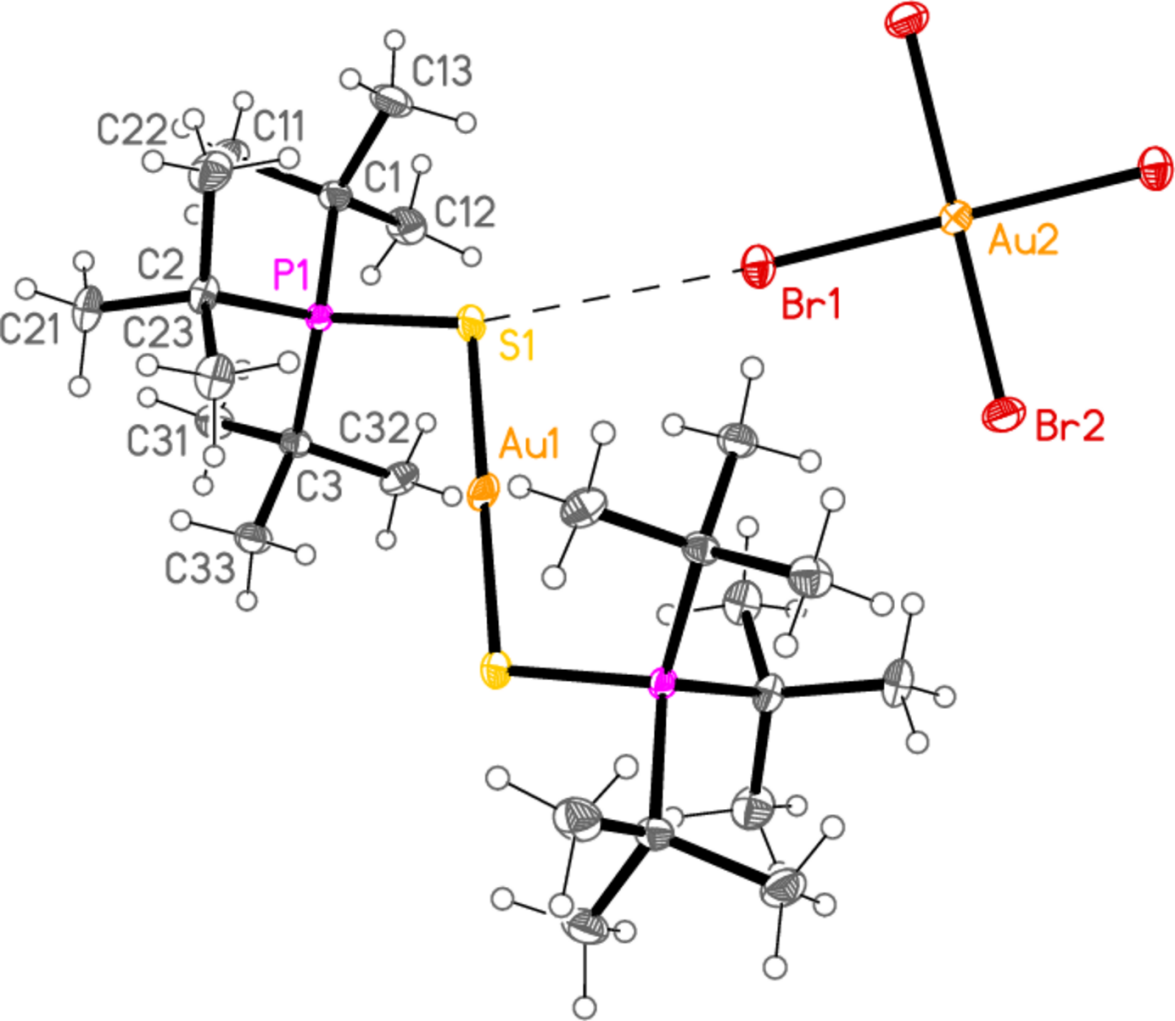
The structure of compound **5a** in the crystal. Only the asymmetric unit is labelled. The contact S1⋯Br1 is indicated by a dashed bond.

**Figure 7:**
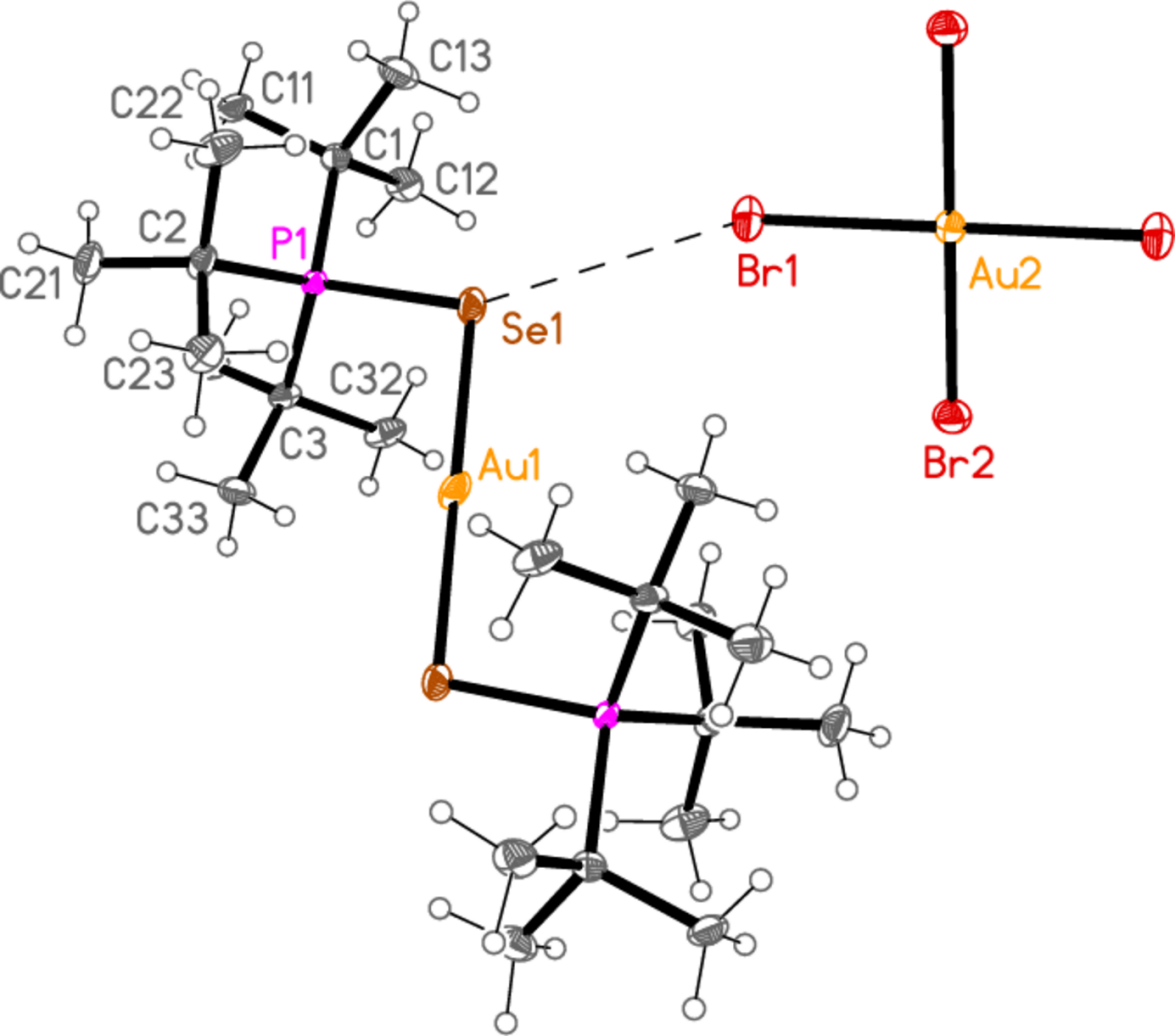
The structure of compound **5b** in the crystal. Only the asymmetric unit is labelled. The contact Se1⋯Br1 is indicated by a dashed bond.

**Figure 8:**
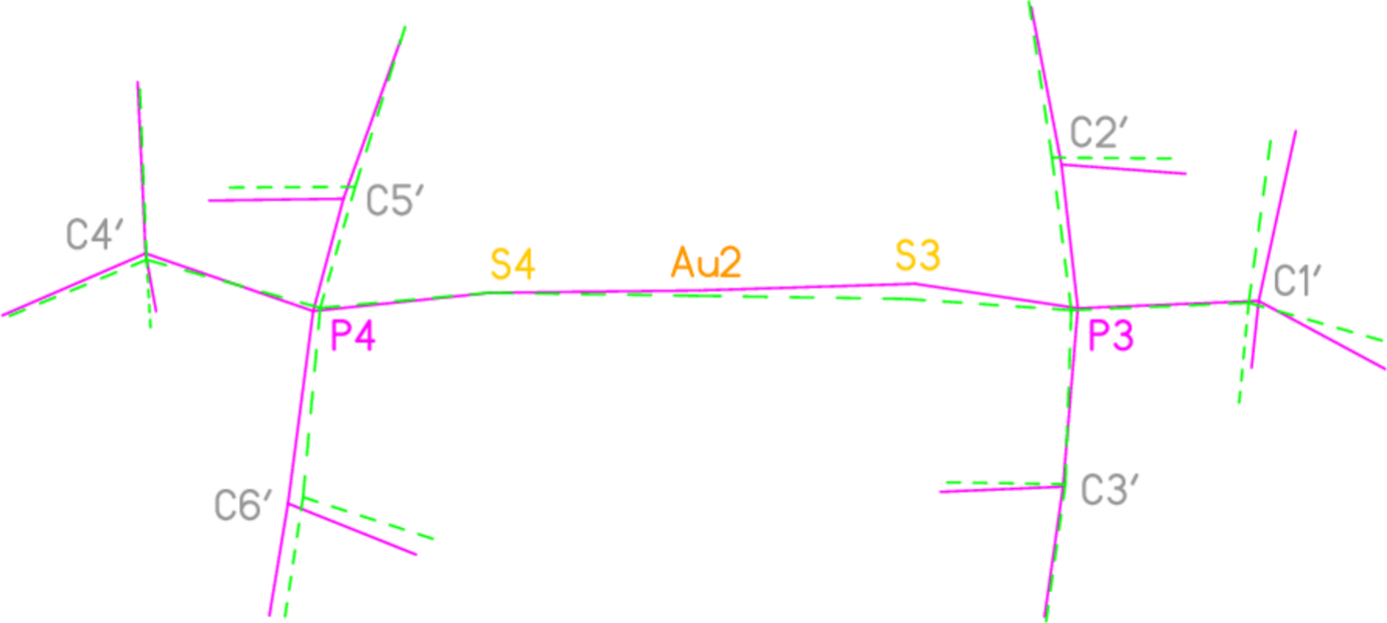
Least-squares fit of the two independent cations of compound **2**. Cation 1 has the dotted bonds; cation 2 (inverted) is labelled.

The asymmetric unit of compound **1**, [(^*i*^Pr_3_PS)_2_Au][AuCl_4_], contains two half cations, with the gold(I) atoms on twofold rotation axes 0.25, *y*, 0.75 (Au1) or 0.25, *y*, 0.25 (Au2) and two half anions, with the gold(III) atoms on inversion centres 0.5, 0.5, 0.5 (Au3) and 0, 0, 0.5 (Au4); the complete cations and anions are shown in Fig. 1 ▸. The three Au—S—P—C torsion angles of the first cation are all roughly 10° larger than those of the second cation. The asymmetric unit of compound **2**, [(^*t*^Bu^*i*^Pr_2_PS)_2_Au][AuCl_4_], contains two cations and two anions with no imposed symmetry (Fig. 2 ▸); the carbon atoms of the second cation are designated with primes. The cations are quite similar, with an r.m.s. deviation of all non-H atoms of 0.193 Å, or 0.117 Å if the carbon atoms are not fitted (Fig. 8 ▸); the numbering of the second cation was chosen carefully to give the best fit for all corresponding atom pairs such as C21/C21′. The asymmetric unit of compound **3**, [(^*t*^Bu_3_PS)_2_Au][AuCl_4_], contains half a cation, with the gold(I) atom (Au1) on the inversion centre 0.5, 0, 0.5 and half an anion, with the gold(III) atom (Au2) on the inversion centre 0.5, 0.5, 1; the complete cation and anion are shown in Fig. 3 ▸. The asymmetric unit of compound **4a**, [(^*t*^Bu^*i*^Pr_2_PS)_2_Au][AuBr_4_], contains a complete cation on a general position and two half anions with the gold(III) atoms on inversion centres (Au2 on 0.5, 0, 0.5 and Au3 on 1, 0.5, 0.5); the cation and both complete anions are shown in Fig. 4 ▸. Compound **4b**, [(^*t*^Bu^*i*^Pr_2_PSe)_2_Au][AuBr_4_] (Fig. 5 ▸), is isotypic with **4a**. The asymmetric unit of compound **5a**, [(^*t*^Bu_3_PS)_2_Au][AuBr_4_], contains half a cation, with the gold(I) atom Au1 on the inversion centre 0.5, 0.5, 0.5, and half an anion, with the gold(III) atom Au2 on the inversion centre 0.5, 1, 1; Fig. 6 ▸ shows a complete cation and anion. Compound **5b**, [(^*t*^Bu_3_PSe)_2_Au][AuBr_4_] (Fig. 7 ▸), is effectively isotypic with **5a**, although there are some appreciable differences in unit cell parameters and in some aspects of the structures (e.g. P—*E*—Au and *E*⋯Br—Au angles, see below).

The crowding effect of the bulky alkyl groups is seen in the short intra­molecular H⋯Au and H⋯*E* contacts, such as H32*C*⋯Au1 2.70 Å and H12*B*⋯S1 2.75 Å for compound **1**. The angles C—H⋯Au and (especially) C—H⋯*E* are necessarily narrow. These contacts are included for convenience in Tables 10 ▸–16 ▸ ▸ ▸ ▸ ▸ ▸. The ligands at the Au^I^ atoms are anti­periplanar to each other across the S⋯S vectors, with P—S⋯S—P torsion angles of exactly 180° (by symmetry) for **3**, **5a** and **5b**. Other values are 150.01 (8) (the largest deviation from 180°) and 173.16 (8)° for **1**, 169.90 (8) and −163.51 (9)° for **2**, 169.04 (14)° for **4a** and 170.12 (4)° for **4b**.

The ten P—S bond lengths lie in the narrow range 2.0263 (13)–2.0384 (7), av. 2.0322 Å; the three P—Se bond lengths are 2.1864 (10)–2.2009 (6) Å, av. 2.1933 Å. These are closely similar to the averages of 2.0368 and 2.1938 Å observed for the gold(I) halide derivatives (Upmann *et al.*, 2024*a* ▸). The ten S—Au bond lengths are 2.2869 (9)–2.299 (2) Å, av. 2.2915 Å, and the three Se—Au bond lengths are 2.4017 (4)–2.4057 (4) Å, av. 2.4037 Å. These compare best to the corresponding bond lengths *trans* to iodine, 2.2959 Å (one value only) for *E* = S and 2.4017 Å (av. of three values) for *E* = Se in the complexes with gold(I) halides.

The P—S—Au angles are 101.88 (5)–107.87 (2)°, av. 104.05°, but the three largest values (for **3**, **5a** and one of four values for **2**) might be considered outliers. One possible explanation for this might be the steric effects of ^*t*^Bu_3_P groups, and another might be the additional short S⋯*X* contacts (see next section), but neither of these possible causes applies to **2**, nor is **5b** affected in the same way despite being isotypic with **5a**. The P—Se—Au angles are 98.27 (3)–101.806 (19)°, av. 100.26°; corresponding average values for the gold(I) halide derivatives were somewhat larger, at 106.17 and 103.86°, respectively. The *E*—P—C angles tend to be narrower for the atoms C1/C4.

## 3. Supra­molecular features

The exterior surface of the [(*R*^1^*R*^2^*R*^3^P*E*)_2_Au]^+^ cations consists, to a considerable extent, of hydrogen atoms. In the absence of classical hydrogen-bond donors, the packing energy is thus likely to be determined by a large number of weakly attractive C—H⋯*X* hydrogen bonds or H⋯H van der Waals inter­actions rather than a small number of short contacts between heavier atoms, a principle that has been expounded convincingly by Dance (2003 ▸). Nonetheless, packing diagrams need to be as simple as possible to be readily inter­preted. Accordingly, the following discussion attempts to show only the main features of the crystal packing, at the risk of oversimplification. Not all H⋯*X* hydrogen bonds are discussed, but are given in Tables 10 ▸ ▸–16 ▸ ▸ ▸ ▸ ▸ ▸ for completeness. Numerical details for contacts of the form *E*⋯*X* for all compounds are summarized in Table 9 ▸. In all packing diagrams, the atom labels indicate the asymmetric unit; hydrogen atoms not involved in H⋯*X* contacts (and some methyl groups, see individual captions for details) have been omitted for clarity.

**Figure 9:**
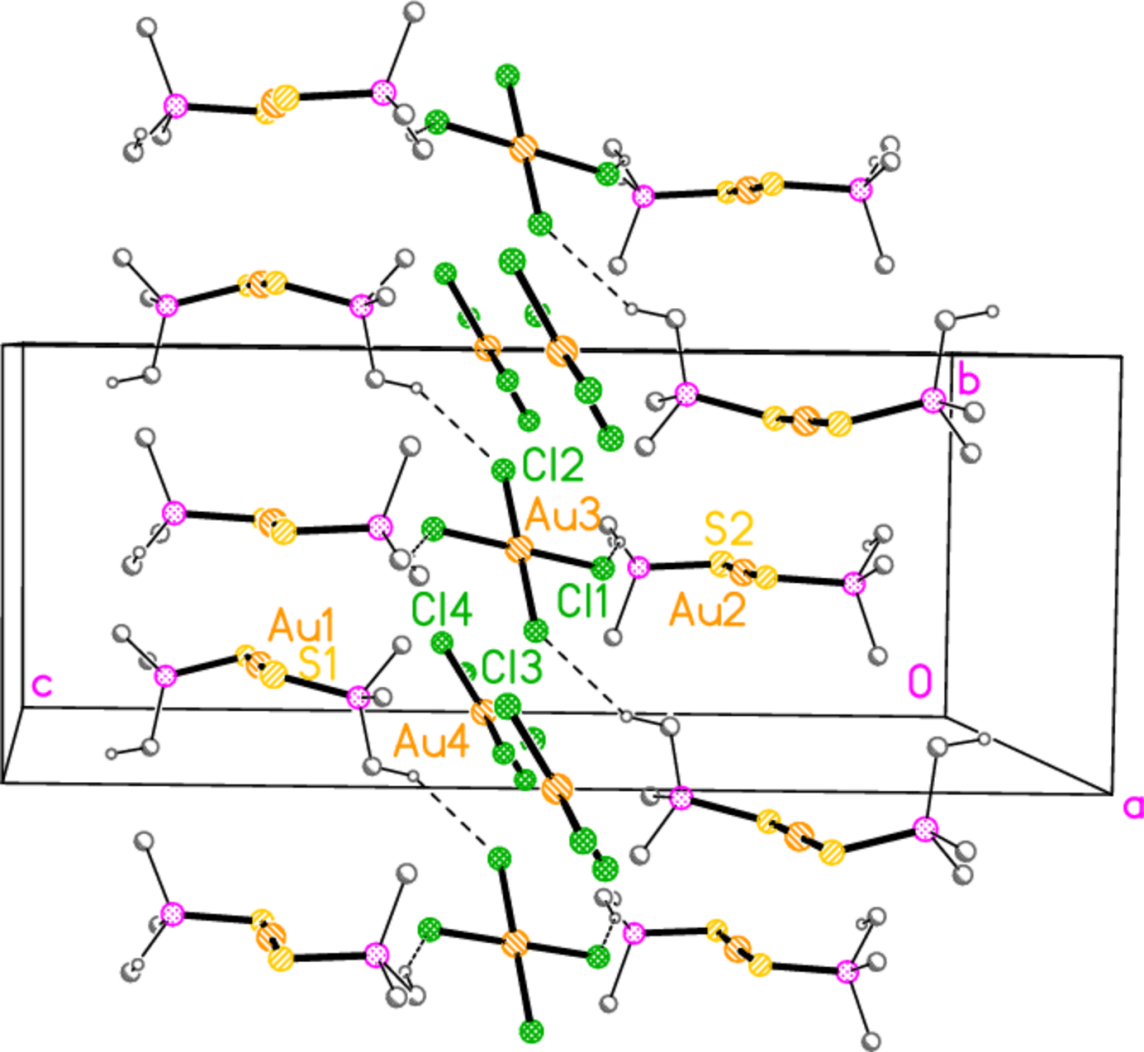
Packing diagram of compound **1** viewed perpendicular to the *bc* plane. Methyl groups are omitted for clarity. Dashed lines indicate the two short H⋯Cl contacts.

**Figure 10:**
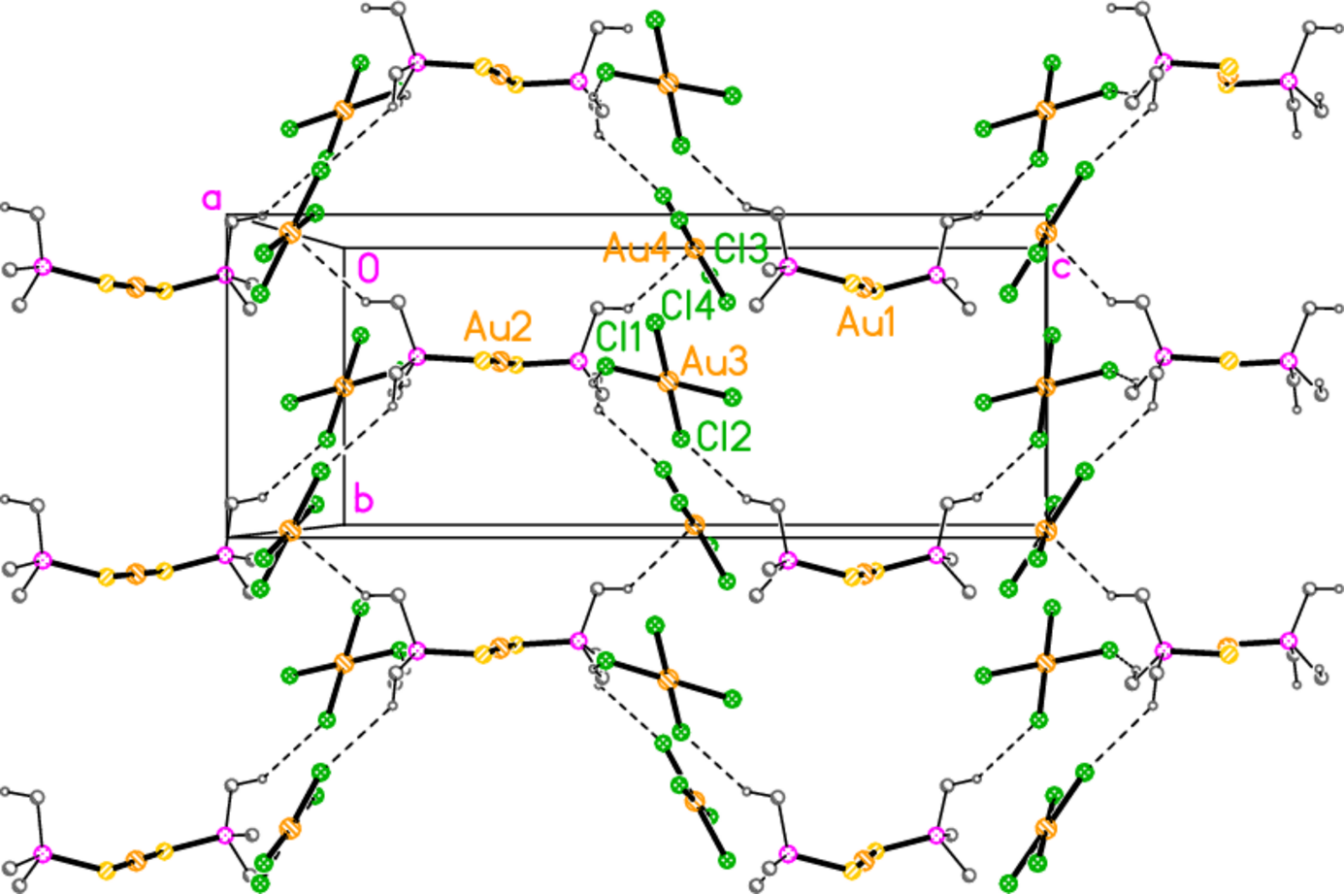
Packing diagram of compound **1**; the view from Fig. 9 ▸ has been extended to include two significantly longer contacts (see text).

The strongest hydrogen-bond donors are likely to be the methine hydrogen atoms of the isopropyl groups. In compound **1**, the shortest such contacts are H3⋯Cl2(*x*, −1 + *y*, *z*) = 2.79 Å and H5⋯Cl1 = 2.75 Å. Even for these two contacts, the crystallographic symmetry of cations and anions leads to a complex three-dimensional packing. A section of this is shown in Fig. 9 ▸, but has the obvious fault that the second anion (centred on Au4) seems to exist in a packing vacuum. The inclusion of the longer contacts H4⋯Cl4(−*x*, 1 − *y*, 1 − *z*) = 2.93 Å and H6⋯Au4 = 3.24 Å provides further information (Fig. 10 ▸); the latter might be regarded as a borderline case of a C—H⋯Au hydrogen bond (Schmidbaur, 2019 ▸; Schmidbaur *et al.*, 2014 ▸).

**Figure 11:**
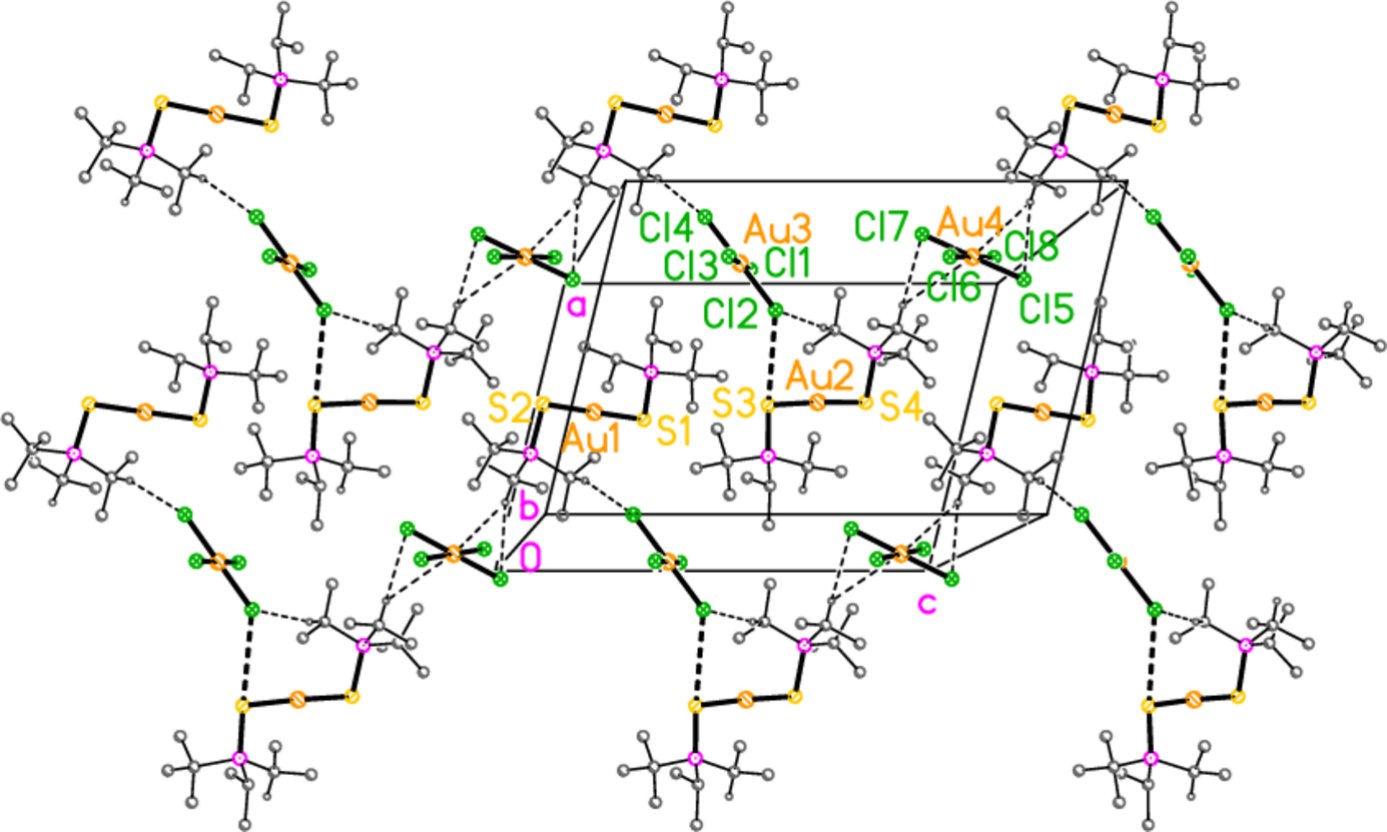
Packing diagram of compound **2** viewed perpendicular to the *ac* plane in the region *y* ≃ 0.25. Dashed lines indicate H⋯Cl or H⋯Au contacts (thin) or S⋯Cl contacts (thick).

For compound **2**, the methine hydrogen atoms again play an important role. Four C—H⋯Cl inter­actions (Table 11 ▸) combine to produce zigzag chains of residues parallel to the *c* axis (Fig. 11 ▸). The atom H6′ also has a short contact to Au4 and may thus be part of a three-centre inter­action. Additionally, the contact S3⋯Cl2, 3.6623 (15) Å, may be regarded as a significant inter­action; it would qualify as a ‘chalcogen bond’ (Aakeroy *et al.*, 2019 ▸; Vogel *et al.*, 2019 ▸), equivalent to the better known halogen bonds (see *e.g.* Metrangolo *et al.*, 2008 ▸). For all the *E*⋯*X* contacts in this paper, the P—*E*⋯*X* angles are reasonably close to linear [range 154.01 (3)–174.74 (5)°], as would be expected for a chalcogen bond, where the positive hole at the donor atom *E* should lie in the extension of the *P*—*E* vector beyond the atom *E*. The *E*⋯*X*—Au angles are also roughly linear [range 140.09 (2)–173.46 (1)°]. Initially, we subjectively judged the corres­ponding distance S1⋯Cl4(−1 + *x*, *y*, *z*] = 3.8505 (15) Å to be too long to be significant, and thus excluded it from the packing diagram. Closer inspection shows, however, that it plays an equivalent role to S3⋯Cl2, thereby linking the chains to form a layer structure parallel to the *ac* plane; this can be seen (implicitly) in Fig. 11 ▸. This shows the pitfalls in judging the importance of weak inter­actions based solely on inter­atomic distances. The contact H3′⋯Cl3 links the parent layer at *y* ≃ 0.25 with its inverted counterpart at *y* ≃ 0.75.

**Figure 12:**
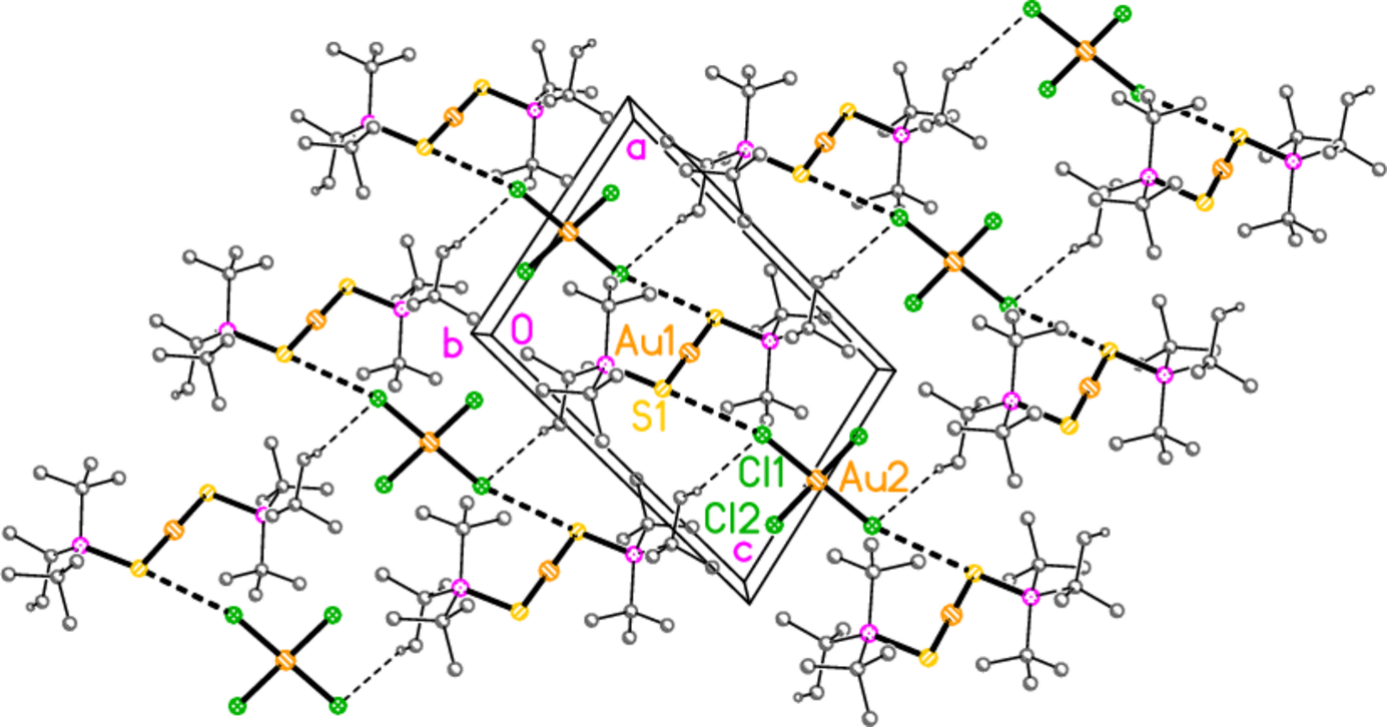
Packing diagram of compound **3** viewed perpendicular to the *ac* plane in the region *y* ≃ 0. Dashed lines indicate H⋯Cl contacts (thin) or S⋯Cl contacts (thick).

The *tert*-butyl derivative **3** contains only methyl hydrogens. The atom Cl1 is involved in the two shortest H⋯Cl contacts and in the contact S1⋯Cl1. The combination of S1⋯Cl1 and the H22*A*⋯Cl1 inter­action leads to a layer structure parallel to the *ac* plane (Fig. 12 ▸). Alternatively, the combination of S1⋯Cl1 and H12*A*⋯Cl1 leads to a layer structure parallel to the *bc* plane (not shown).

For the bromido derivatives, associations of the anions form a readily recognizable part of the packing patterns; we have presented several structures involving loosely connected [Au*X*_4_]^−^ networks in a previous paper (Döring & Jones, 2016 ▸).

**Figure 13:**
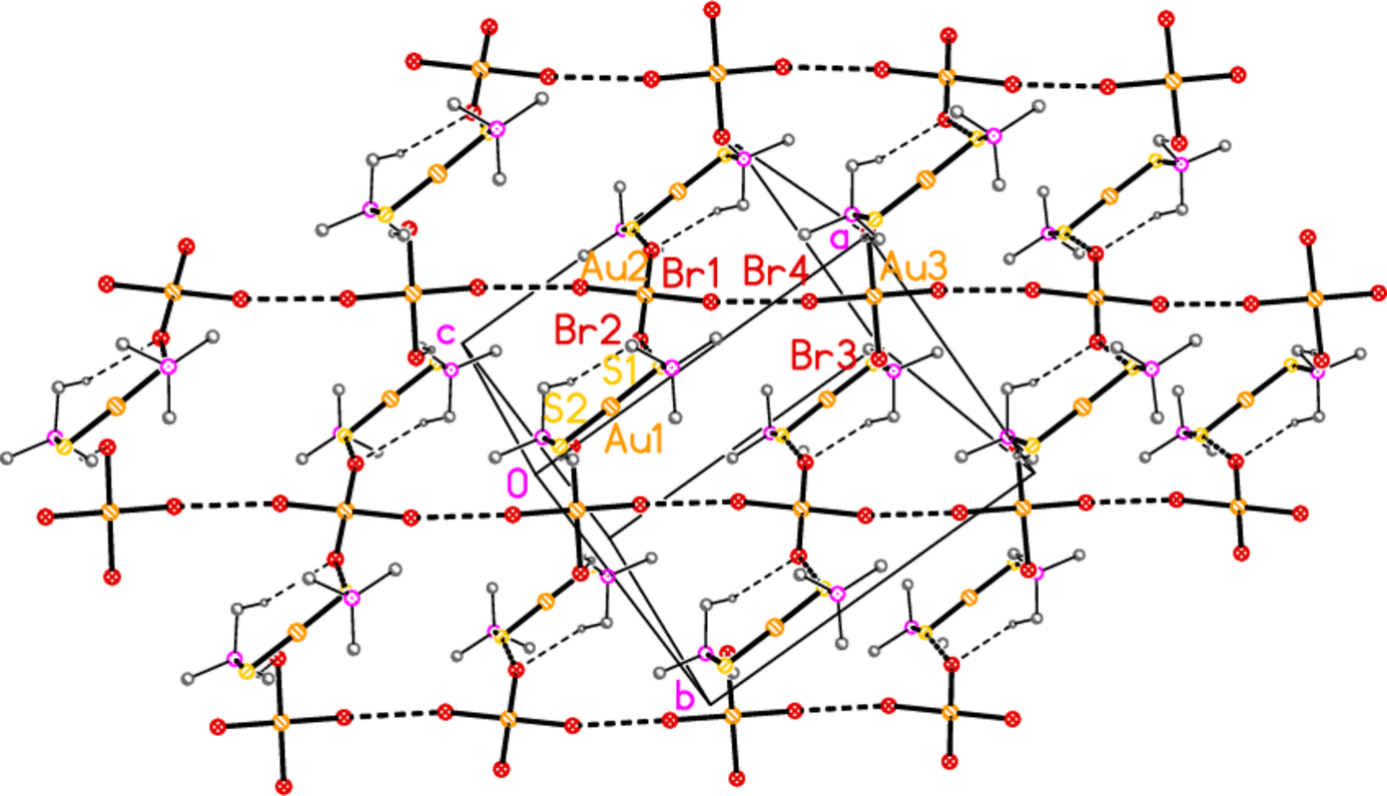
Packing diagram of compound **4a**. The layer structure is parallel to the *ab* plane, but for clarity has been rotated significantly from the ideal view direction perpendicular to this plane. The region *z* ≃ 0 is shown. Methyl groups are omitted. Dashed lines indicate H⋯Br contacts (thin) or Br⋯Br and S⋯Br contacts (thick).

For compound **4a**, there are no strikingly short contacts. An acceptable view of the packing as a layer parallel to the *ab* plane in the region *z* ≃ 0.5 can, however, be assembled as shown in Fig. 13 ▸, based on the heavy-atom distances Br1⋯Br4 = 3.9737 (14) and S1⋯Br2 = 3.746 (3) Å. Contact angles are Au2—Br1⋯Br4 = 158.85 (4) and Au3—Br4⋯Br1 = 153.36 (4). The Br⋯Br contacts link the anions to form a chain parallel to [110], and the S⋯Br contacts link one set of anions to the cations. Two of the four borderline H⋯Br hydrogen bonds (from H5 and H6) are also involved in this layer. In the corresponding layer at *z* ≃ 0, the anion chains run parallel to [1
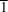
0]. The isotypic compound **4b** necessarily has the same general packing features. The Br1⋯Br4 distance is still very long at 4.0054 (6) Å, but the Se1⋯Br2 contact is shorter than S1⋯Br2 in **4a**. Inter­anionic contact angles are Au2—Br1⋯Br4 = 159.76 (2) and Au3—Br4⋯Br1 = 153.43 (2).

**Figure 14:**
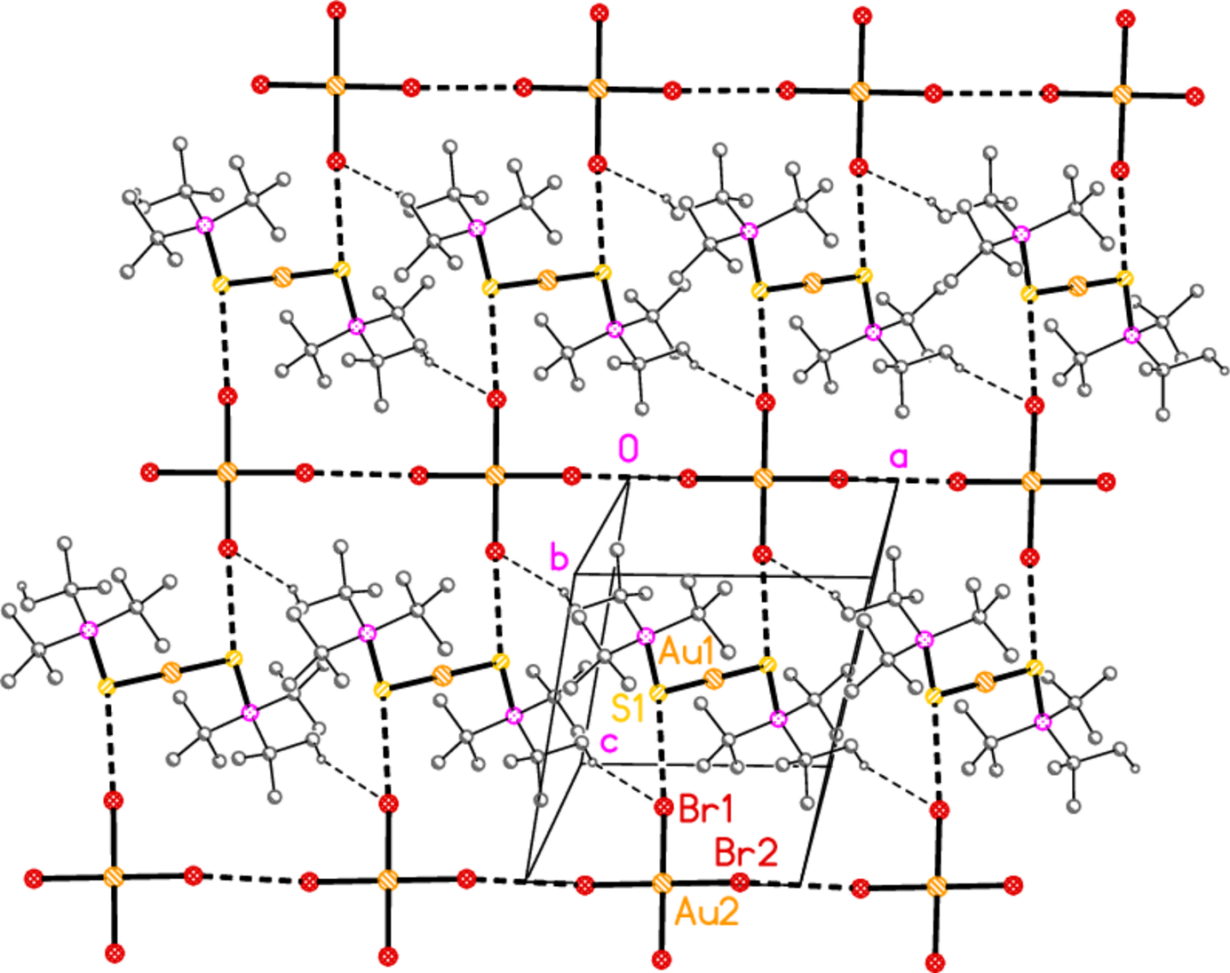
Packing diagram of compound **5a**. The layer structure is parallel to the *ab* plane, but for clarity is viewed approximately perpendicular to [01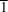]. Dashed lines indicate H⋯Br contacts (thin) or Br⋯Br and S⋯Br contacts (thick).

The packing of **5a** (Fig. 14 ▸) is similar to that of **4a**, but with chains of anions parallel to the *a* axis linked by the contact Br2⋯Br2(2 − *x*, 2 − *y*, 2 − *z*) = 3.7582 (5) Å. The chains are crosslinked *via* the contacts S1⋯Br2 and H22*C*⋯Br1(−*x*, 1 − *y*, 1 − *z*) = 2.88 Å, the shortest H⋯Br contact, to form a layer structure parallel to the *ab* plane. The inter­anionic contact angle is Au2—Br2⋯Br2′ = 160.34 (1). The corres­ponding values for the isotypic compound **5b** are: Br2⋯Br2′ = 3.7404 (5), H22*C*⋯Br1′ = 2.88 Å and Au2—Br2⋯Br2′ = 157.73 (1). Despite the isotypy, the *E*⋯*X*—Au angles for **5a** and **5b** differ by more than 13° (Table 9 ▸).

## 4. Database survey

The search employed the routine ConQuest (Bruno *et al.*, 2002 ▸), part of version 2024.1.0 of the Cambridge Structural Database (CSD; Groom *et al.*, 2016 ▸).

Only four structures with a bis­(phosphane chalcogenido)gold(I) cation were found in the Database, and all of these involved tri­phenyl­phosphane: bis­(tri­phenyl­phosphane sulf­ido)gold(I) di­fluoro­phosphate (refcode RIVZUR; LeBlanc *et al.*, 1997 ▸) and three structures from our own work, namely bis­(tri­phenyl­phosphane selenido)gold(I) hexa­fluor­ido­anti­monate (SOHCIB; Jones & Thöne, 1991 ▸), and the as yet unpublished (but deposited) structures bis­(tri­phenyl­phosphane sulfido)­gold(I) nitrate and bis­(tri­phenyl­phosphane sulfido)­gold(I) bis­(methane­sulfon­yl)amide bis­(methane­sulf­on­yl)amine di­chloro­methane solvate (UREBOK and UREBUQ; Jones & Geissler, 2016*a* ▸,*b* ▸). The five Au—S bond lengths lie in the range 2.277 (2)–2.2963 (3), av. 2.2893 Å and the two Au—Se bond lengths are 2.390 (1) and 2.395 (1) Å, *cf*. the average values in this paper of Au—S = 2.2915 and Au—Se = 2.4037 Å.

## 5. Synthesis and crystallization

Compound **1**: The gold(I) precursor ^*i*^Pr_3_PSAuCl (212 mg, 0.5 mmol) was dissolved in 10 ml of di­chloro­methane, and a solution of di­chloro­(phen­yl)-λ^3^-iodane (‘iodo­phenyl dichloride’, PhICl_2_; 344 mg, 1.25 mmol) was added dropwise. The solution initially turned red [presumably because of the formation of a gold(III) inter­mediate] but became yellow after 20 min stirring. The solvent was removed *in vacuo* and the residue redissolved in di­chloro­methane. The solution was overlayered with *n*-pentane and left to stand for 3 d in a refrigerator (276 K), after which yellow crystals had formed. ^31^P NMR: δ 78.86 ppm (*s*). Compounds **2** and **3** were synthesized from the appropriate gold(I) precursors in the same way as **1**. Unfortunately, details were lost when my (PGJ) research group disbanded in 2018.

Compound **4a**: ^*i*^Pr_2_^*t*^BuPSAuBr (327 mg, 0.677 mmol) was dissolved in 3 ml of di­chloro­methane and 6.7 ml of a stock bromine solution (0.1 *M* in di­chloro­methane) was added. After stirring, the solution was overlayered with *n*-pentane and stored in the refrigerator for 5 d. The red crystals thus obtained were suitable for X-ray diffraction analysis. Yield: 324 mg, 0.287 mmol, 85%. ^31^P{^1^H}-NMR (81.01 MHz, CDCl_3_, 300 K): δ = 81.6 ppm (*s*). Elemental analysis [%]: calc.: C 21.33, H 4.12, S 5.69; found: C19.94, H 3.82, S 6.08.

Compound **4b**: ^*i*^Pr_2_^*t*^BuPSeAuBr (369 mg, 0.696 mmol) was dissolved in 3 ml of di­chloro­methane and 6.9 ml of the stock bromine solution was added. After stirring, the solution was overlayered with *n*-pentane and stored in the refrigerator for 4 d. Since no formation of crystals or precipitation of the desired product was observed, the solvents were removed under reduced pressure and the red product was recrystallized from di­chloro­methane by overlayering with *n*-pentane. Yield: 316 mg, 0.259 mmol, 75%. ^31^P{^1^H}-NMR: δ = 80.2 ppm (*s* with P—Se satellites, ^1^*J*_P–Se_ = 528 Hz). Elemental analysis [%]: calc.: C 19.69, H 3.80; found: C18.14, H 3.45. Single crystals suitable for X-ray diffraction analysis were obtained from a solution in CDCl_3_ overlayered with *n*-pentane.

Compound **5a**: ^*t*^Bu_3_PSAuBr (134 mg, 0.263 mmol) was dissolved in 3 ml of di­chloro­methane and 2.6 ml of the stock bromine solution was added. After stirring, the solution was overlayered with *n*-pentane and stored in the refrigerator for 7 d. Instead of the formation of crystals or precipitation of the desired product, an oily residue was obtained. The solvents were removed under reduced pressure, the product was redissolved in a very small amount of di­chloro­methane, and the product was precipitated by overlayering with *n*-pentane. No crystallization was observed after 4 d; the solvents were again removed under reduced pressure. A third crystallization attempt, from a solution in di­chloro­methane overlayered with *n*-pentane, was then successful; red crystals suitable for X-ray diffraction analysis were obtained. Yield: 91 mg, 0.077 mmol, 59%. ^31^P{^1^H}-NMR: δ = 89.2 ppm (*s*). Elemental analysis [%]: calc.: C 24.38, H 4.60, S 5.42; found: C23.02, H 4.30, S 5.47.

Compound **5b**: ^*t*^Bu_3_PSeAuBr (154 mg, 0.276 mmol) was dissolved in 3 ml of di­chloro­methane and 3.3 ml of the stock bromine solution was added. After stirring, the solution was overlayered with *n*-pentane and stored in the refrigerator for 7 d. An oily residue was obtained. The solvents were removed under reduced pressure, the product was redissolved in a very small amount of di­chloro­methane, and the product precipitated by overlayering with *n*-pentane. Red crystals suitable for X-ray diffraction analysis were obtained after several recrystallizations from di­chloro­methane solutions overlayered with *n*-pentane. Additional crystals were found in this sample and were identified as [(^*t*^Bu_2_POSe)_2_H]^+^[AuBr_4_]^−^ by X-ray diffraction (structure to be reported in Part 10 of this series). Yield: 68 mg, 0.053 mmol, 38% (but this includes the impur­ities). ^31^P{^1^H}-NMR: δ = 87.0 ppm (*s* with P—Se satellites, ^1^*J*_P–Se_ = 549 Hz). Elemental analysis [%]: calc.: C 22.59, H 4.27; found: C19.71, H 3.74.

## 6. Refinement

Details of the measurements and refinements are given in Table 17 ▸. Methine hydrogen atoms were included at calculated positions and refined using a riding model with C—H 1.00 Å and *U*_iso_(H) = 1.2×*U*_eq_(C). Methyl groups were refined, using the command ‘AFIX 137’, as idealized rigid groups allowed to rotate (from a starting position determined from difference peaks) but not to tip, with C—H = 0.98 Å, H—C—H = 109.5° and *U*_iso_(H) = 1.5×*U*_eq_(C). This procedure is less reliable for heavy-atom structures, so that any postulated hydrogen bonds involving methyl hydrogen atoms should be inter­preted with caution.

*Special features*: For compounds **3** and **5a**, an extinction correction (Sheldrick, 2015 ▸) was applied. The data for **4a**, measured using a thin and weakly diffracting crystal plate, are appreciably worse than for the other structures, with a maximum electron density peak of 4.2 e Å^−3^ at 0.95 Å from Br1. Residual absorption errors are thus a likely cause of the large difference peak(s). However, the presence of the largest peak near a bromine rather than a gold atom means that some slight disorder of the anions cannot be ruled out; the components would have to be very close to each other. The data for the isotypic selenium derivative **4b** are of much better quality.

## Supplementary Material

Crystal structure: contains datablock(s) 1, 2, 3, 4a, 4b, 5a, 5b, global. DOI: 10.1107/S2056989024009095/wm5733sup1.cif

Structure factors: contains datablock(s) 1. DOI: 10.1107/S2056989024009095/wm57331sup2.hkl

Structure factors: contains datablock(s) 2. DOI: 10.1107/S2056989024009095/wm57332sup3.hkl

Structure factors: contains datablock(s) 3. DOI: 10.1107/S2056989024009095/wm57333sup4.hkl

Structure factors: contains datablock(s) 4a. DOI: 10.1107/S2056989024009095/wm57334asup5.hkl

Structure factors: contains datablock(s) 4b. DOI: 10.1107/S2056989024009095/wm57334bsup6.hkl

Structure factors: contains datablock(s) 5a. DOI: 10.1107/S2056989024009095/wm57335asup7.hkl

Structure factors: contains datablock(s) 5b. DOI: 10.1107/S2056989024009095/wm57335bsup8.hkl

CCDC references: 2156392, 2156788, 2156791, 2156792, 2156872, 2156873, 2156878

Additional supporting information:  crystallographic information; 3D view; checkCIF report

## Figures and Tables

**Table 1:** Compositions of the [(*R*^1^*R*^2^*R*^3^P*E*)_2_Au]^+^[Au*X*_4_]^−^ structures presented in this paper (see Scheme)

Compound	*R* ^1^	*R* ^2^	*R* ^3^	*E*	*X*
**1**	^*i*^Pr	^*i*^Pr	^*i*^Pr	S	Cl
**2**	^*i*^Pr	^*i*^Pr	^*t*^Bu	S	Cl
**3**	^*t*^Bu	^*t*^Bu	^*t*^Bu	S	Cl
**4a**	^*i*^Pr	^*i*^Pr	^*t*^Bu	S	Br
**4b**	^*i*^Pr	^*i*^Pr	^*i*^Bu	Se	Br
**5a**	^*i*^Bu	^*i*^Bu	^*t*^Bu	S	Br
**5b**	^*i*^Bu	^*t*^Bu	^*t*^Bu	Se	Br

**Table 2:** Selected geometric parameters (Å, °) for **1**[Chem scheme1]

Au1—S1	2.2918 (10)	Au3—Cl2	2.2833 (10)
P1—S1	2.0369 (14)	Au3—Cl1	2.2865 (10)
Au2—S2	2.2970 (9)	Au4—Cl3	2.2811 (10)
P2—S2	2.0310 (13)	Au4—Cl4	2.2849 (9)
			
S1—Au1—S1^i^	179.97 (5)	Cl2—Au3—Cl1	89.99 (4)
C1—P1—S1	107.31 (15)	Cl2—Au3—Cl1^iii^	90.01 (4)
P1—S1—Au1	102.05 (5)	Cl1—Au3—Cl1^iii^	180.0
S2^ii^—Au2—S2	177.70 (5)	Cl3^iv^—Au4—Cl3	180.0
C4—P2—S2	107.67 (13)	Cl3—Au4—Cl4^iv^	89.63 (4)
P2—S2—Au2	102.69 (5)	Cl3—Au4—Cl4	90.37 (4)
Cl2—Au3—Cl2^iii^	180.0	Cl4^iv^—Au4—Cl4	180.0
			
C1—P1—S1—Au1	171.60 (15)	C4—P2—S2—Au2	162.32 (12)

Symmetry codes: (i) 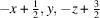; (ii) 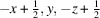; (iii) 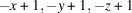; (iv) 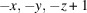.

**Table 3:** Selected geometric parameters (Å, °) for **2**[Chem scheme1]

Au1—S1	2.2869 (9)	Au3—Cl4	2.2746 (10)
Au1—S2	2.2910 (9)	Au3—Cl1	2.2754 (11)
P1—S1	2.0283 (14)	Au3—Cl2	2.2805 (10)
P2—S2	2.0263 (13)	Au3—Cl3	2.2852 (11)
Au2—S4	2.2935 (9)	Au4—Cl5	2.2780 (10)
Au2—S3	2.2953 (9)	Au4—Cl6	2.2825 (11)
P3—S3	2.0360 (14)	Au4—Cl7	2.2828 (10)
P4—S4	2.0312 (13)	Au4—Cl8	2.2839 (10)
			
S1—Au1—S2	179.28 (4)	Cl4—Au3—Cl2	179.22 (4)
C1—P1—S1	105.10 (13)	Cl1—Au3—Cl2	89.46 (4)
C4—P2—S2	105.95 (12)	Cl4—Au3—Cl3	90.14 (4)
P1—S1—Au1	101.88 (5)	Cl1—Au3—Cl3	179.77 (4)
P2—S2—Au1	102.91 (5)	Cl2—Au3—Cl3	90.64 (4)
S4—Au2—S3	177.24 (4)	Cl5—Au4—Cl6	89.61 (4)
C1′—P3—S3	106.48 (15)	Cl5—Au4—Cl7	179.44 (4)
C4′—P4—S4	106.46 (13)	Cl6—Au4—Cl7	90.15 (4)
P3—S3—Au2	103.22 (5)	Cl5—Au4—Cl8	90.54 (4)
P4—S4—Au2	106.42 (5)	Cl6—Au4—Cl8	179.58 (4)
Cl4—Au3—Cl1	89.76 (4)	Cl7—Au4—Cl8	89.70 (4)
			
C1—P1—S1—Au1	176.99 (13)	C1′—P3—S3—Au2	−179.04 (18)
C4—P2—S2—Au1	166.51 (12)	C4′—P4—S4—Au2	−162.56 (13)

**Table 4:** Selected geometric parameters (Å, °) for **3**[Chem scheme1]

Au1—S1	2.2889 (5)	Au2—Cl1	2.2802 (5)
P1—S1	2.0374 (6)	Au2—Cl2	2.2836 (5)
			
S1—Au1—S1^i^	180.0	Cl1—Au2—Cl2	89.664 (18)
C1—P1—S1	101.57 (6)	Cl1—Au2—Cl2^ii^	90.336 (18)
P1—S1—Au1	107.87 (2)	Cl2—Au2—Cl2^ii^	180.0
Cl1^ii^—Au2—Cl1	180.0		
			
C1—P1—S1—Au1	−172.57 (6)		

Symmetry codes: (i) 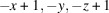; (ii) 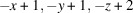.

**Table 5:** Selected geometric parameters (Å, °) for **4a**[Chem scheme1]

Au1—S1	2.291 (2)	Au2—Br2	2.4196 (10)
Au1—S2	2.299 (2)	Au2—Br1	2.4421 (11)
S1—P1	2.028 (3)	Au3—Br3	2.4238 (9)
S2—P2	2.028 (3)	Au3—Br4	2.4294 (8)
			
S1—Au1—S2	178.28 (8)	Br2—Au2—Br1^i^	89.52 (4)
P1—S1—Au1	102.39 (11)	Br1—Au2—Br1^i^	180.0
P2—S2—Au1	103.89 (11)	Br3^ii^—Au3—Br3	180.0
C1—P1—S1	106.3 (3)	Br3—Au3—Br4	90.52 (3)
C4—P2—S2	105.9 (3)	Br3—Au3—Br4^ii^	89.48 (3)
Br2^i^—Au2—Br2	180.0	Br4—Au3—Br4^ii^	180.0
Br2—Au2—Br1	90.48 (4)		
			
Au1—S1—P1—C1	175.0 (4)	Au1—S2—P2—C4	−164.7 (3)

Symmetry codes: (i) 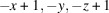; (ii) 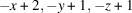.

**Table 6:** Selected geometric parameters (Å, °) for **4b**[Chem scheme1]

Au1—Se1	2.4017 (4)	Au2—Br2	2.4254 (4)
Au1—Se2	2.4057 (4)	Au2—Br1	2.4337 (4)
Se1—P1	2.1929 (10)	Au3—Br3	2.4285 (4)
Se2—P2	2.1864 (10)	Au3—Br4	2.4320 (4)
			
Se1—Au1—Se2	176.734 (16)	Br2—Au2—Br1^i^	89.419 (15)
P1—Se1—Au1	98.27 (3)	Br1—Au2—Br1^i^	180.0
P2—Se2—Au1	100.69 (3)	Br3^ii^—Au3—Br3	180.0
C1—P1—Se1	106.65 (14)	Br3—Au3—Br4^ii^	89.190 (14)
C4—P2—Se2	106.60 (13)	Br3—Au3—Br4	90.809 (14)
Br2—Au2—Br2^i^	180.0	Br4^ii^—Au3—Br4	180.0
Br2—Au2—Br1	90.581 (15)		
			
Au1—Se1—P1—C1	173.20 (15)	Au1—Se2—P2—C4	−163.72 (13)

Symmetry codes: (i) 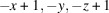; (ii) 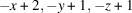.

**Table 7:** Selected geometric parameters (Å, °) for **5a**[Chem scheme1]

Au1—S1	2.2891 (5)	Au2—Br1	2.4245 (2)
P1—S1	2.0384 (7)	Au2—Br2	2.4260 (2)
			
S1^i^—Au1—S1	180.0	Br1—Au2—Br2^ii^	89.687 (9)
C1—P1—S1	101.58 (7)	Br1—Au2—Br2	90.312 (9)
P1—S1—Au1	107.15 (3)	Br2^ii^—Au2—Br2	180.0
Br1—Au2—Br1^ii^	180.0		
			
C1—P1—S1—Au1	−171.01 (7)		

Symmetry codes: (i) 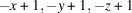; (ii) 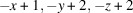.

**Table 8:** Selected geometric parameters (Å, °) for **5b**[Chem scheme1]

Au1—Se1	2.4036 (3)	Au2—Br1	2.4265 (3)
P1—Se1	2.2009 (6)	Au2—Br2	2.4295 (3)
			
C1—P1—Se1	102.74 (8)	Br1—Au2—Br2	90.899 (10)
P1—Se1—Au1	101.806 (19)	Br1—Au2—Br2^i^	89.102 (10)
Br1^i^—Au2—Br1	180.0	Br2—Au2—Br2^i^	180.0
			
C1—P1—Se1—Au1	−169.62 (8)		

Symmetry code: (i) 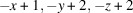.

**Table 9:** Geometric details (Å, °) of *E*⋯*X* contacts

Compound	Contact P—*E*⋯*X*—Au	*E*⋯*X*	P—*E*⋯*X*	*E*⋯*X*—Au
**1**	none			
**2**	P3—S3⋯Cl2—Au3	3.6623 (15)	166.47 (15)	138.96 (4)
**2** ^*a*^	P1—S1⋯Cl4—Au3	3.8505 (15)	174.74 (5)	137.71 (4)
**3**	P1—S1⋯Cl1—Au1	3.5617 (7)	157.52 (2)	165.36 (2)
**4a**	P1—S1⋯Br2—Au1	3.746 (3)	166.39 (13)	146.20 (5)
**4b**	P1—Se1⋯Br2—Au1	3.6251 (6)	173.35 (3)	140.09 (2)
**5a**	P1—S1⋯Br1—Au2	3.5260 (6)	154.01 (3)	173.46 (1)
**5b**	P1—Se1⋯Br1—Au2	3.6563 (4)	154.96 (2)	160.33 (1)

Note: (*a*) Operator for *X*—Au: −1 + *x*, *y*, *z*.

**Table 10:** Hydrogen-bond geometry (Å, °) for **1**[Chem scheme1]

*D*—H⋯*A*	*D*—H	H⋯*A*	*D*⋯*A*	*D*—H⋯*A*
C32—H32*C*⋯Au1	0.98	2.70	3.480 (5)	137
C62—H62*C*⋯Au2	0.98	2.92	3.641 (4)	131
C12—H12*B*⋯S1	0.98	2.75	3.180 (5)	107
C42—H42*B*⋯S2	0.98	2.74	3.240 (4)	112
C21—H21*A*⋯Cl1^iii^	0.98	2.88	3.840 (5)	167
C5—H5⋯Cl1	1.00	2.75	3.670 (4)	153
C3—H3⋯Cl2^v^	1.00	2.79	3.682 (4)	149
C52—H52*A*⋯Cl2	0.98	2.81	3.770 (4)	167
C32—H32*B*⋯Cl3^iv^	0.98	2.94	3.649 (5)	130
C4—H4⋯Cl4^vi^	1.00	2.93	3.726 (4)	137
C6—H6⋯Au4	1.00	3.24	4.022 (4)	136

Symmetry codes: (iii) 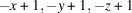; (iv) 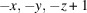; (v) 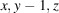; (vi) 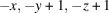.

**Table 11:** Hydrogen-bond geometry (Å, °) for **2**[Chem scheme1]

*D*—H⋯*A*	*D*—H	H⋯*A*	*D*⋯*A*	*D*—H⋯*A*
C32—H32*C*⋯Au1	0.98	2.73	3.538 (4)	140
C32′—H32*D*⋯Au2	0.98	2.73	3.503 (5)	136
C13—H13*B*⋯S1	0.98	2.62	3.220 (5)	120
C43—H43*B*⋯S2	0.98	2.75	3.219 (4)	110
C43′—H43*D*⋯S4	0.98	2.73	3.219 (4)	112
C13′—H13*E*⋯S3	0.98	2.75	3.227 (5)	111
C52′—H52*F*⋯Cl1	0.98	2.82	3.733 (4)	155
C5′—H5′⋯Cl2	1.00	2.87	3.839 (4)	163
C3′—H3′⋯Cl3^i^	1.00	2.88	3.585 (4)	128
C5—H5⋯Cl4^ii^	1.00	2.79	3.713 (4)	154
C62—H62*B*⋯Cl4^ii^	0.98	2.83	3.692 (4)	147
C42—H42*C*⋯Cl5^i^	0.98	2.91	3.754 (4)	145
C11—H11*C*⋯Cl7^iii^	0.98	2.80	3.736 (5)	161
C6′—H6′⋯Cl7	1.00	2.91	3.903 (4)	170
C6′—H6′⋯Au4	1.00	3.28	4.009 (4)	132
C62—H62*A*⋯Cl8^iv^	0.98	2.93	3.853 (4)	157

Symmetry codes: (i) 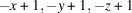; (ii) 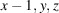; (iii) 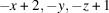; (iv) 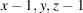.

**Table 12:** Hydrogen-bond geometry (Å, °) for **3**[Chem scheme1]

*D*—H⋯*A*	*D*—H	H⋯*A*	*D*⋯*A*	*D*—H⋯*A*
C23—H23*A*⋯Au1	0.98	2.81	3.421 (2)	121
C33—H33*C*⋯Au1	0.98	2.69	3.5832 (19)	151
C13—H13*A*⋯S1	0.98	2.66	3.164 (2)	112
C32—H32*A*⋯S1	0.98	2.87	3.353 (2)	111
C12—H12*A*⋯Cl1^iii^	0.98	2.91	3.786 (2)	150
C22—H22*A*⋯Cl1^iv^	0.98	2.83	3.607 (2)	136
C23—H23*B*⋯Cl1^i^	0.98	2.94	3.782 (2)	145

Symmetry codes: (i) 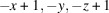; (iii) 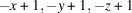; (iv) 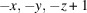.

**Table 13:** Hydrogen-bond geometry (Å, °) for **4a**[Chem scheme1]

*D*—H⋯*A*	*D*—H	H⋯*A*	*D*⋯*A*	*D*—H⋯*A*
C32—H32*C*⋯Au1	0.98	2.75	3.514 (11)	135
C13—H13*C*⋯S1	0.98	2.76	3.212 (11)	109
C43—H43*B*⋯S2	0.98	2.77	3.201 (9)	107
C3—H3⋯Br1^iii^	1.00	3.14	3.809 (9)	126
C52—H52*C*⋯Br1^i^	0.98	3.11	4.055 (9)	161
C5—H5⋯Br2	1.00	3.05	3.997 (9)	158
C62—H62*B*⋯Br2	0.98	3.04	3.883 (12)	145
C2—H2⋯Br3^iii^	1.00	3.12	3.927 (9)	139
C6—H6⋯Br3^iv^	1.00	3.03	3.898 (8)	146
C32—H32*B*⋯Br3^iii^	0.98	3.11	4.023 (11)	156
C42—H42*A*⋯Br3^v^	0.98	2.97	3.848 (10)	149
C62—H62*C*⋯Br4^iv^	0.98	3.08	4.007 (10)	158

Symmetry codes: (i) 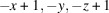; (iii) 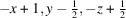; (iv) 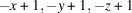; (v) 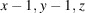.

**Table 14:** Hydrogen-bond geometry (Å, °) for **4b**[Chem scheme1]

*D*—H⋯*A*	*D*—H	H⋯*A*	*D*⋯*A*	*D*—H⋯*A*
C32—H32*C*⋯Au1	0.98	2.73	3.505 (4)	136
C13—H13*C*⋯Se1	0.98	2.80	3.305 (4)	113
C43—H43*B*⋯Se2	0.98	2.84	3.304 (4)	110
C3—H3⋯Br1^iii^	1.00	3.13	3.788 (4)	125
C52—H52*C*⋯Br1^i^	0.98	3.13	4.086 (4)	165
C5—H5⋯Br2	1.00	3.02	3.964 (4)	158
C62—H62*B*⋯Br2	0.98	3.04	3.802 (4)	136
C2—H2⋯Br3^iii^	1.00	3.20	3.990 (4)	137
C6—H6⋯Br3^iv^	1.00	3.02	3.870 (4)	144
C32—H32*B*⋯Br3^iii^	0.98	3.06	3.985 (4)	158
C42—H42*A*⋯Br3^v^	0.98	3.03	3.897 (4)	148
C62—H62*C*⋯Br4^iv^	0.98	3.10	4.018 (4)	158

Symmetry codes: (i) 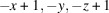; (iii) 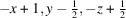; (iv) 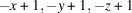; (v) 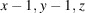.

**Table 15:** Hydrogen-bond geometry (Å, °) for **5a**[Chem scheme1]

*D*—H⋯*A*	*D*—H	H⋯*A*	*D*⋯*A*	*D*—H⋯*A*
C33—H33*C*⋯Au1	0.98	2.69	3.567 (2)	150
C23—H23*A*⋯Au1	0.98	2.86	3.421 (2)	118
C22—H22*C*⋯Br1^iii^	0.98	2.88	3.756 (2)	150
C12—H12*A*⋯Br1^iv^	0.98	3.05	3.890 (2)	145
C32—H32*A*⋯S1	0.98	2.86	3.338 (2)	111
C23—H23*A*⋯S1	0.98	2.98	3.456 (2)	111
C13—H13*A*⋯S1	0.98	2.67	3.160 (2)	112

Symmetry codes: (iii) 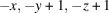; (iv) 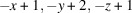.

**Table 16:** Hydrogen-bond geometry (Å, °) for **5b**[Chem scheme1]

*D*—H⋯*A*	*D*—H	H⋯*A*	*D*⋯*A*	*D*—H⋯*A*
C33—H33*C*⋯Au1	0.98	2.64	3.540 (3)	152
C23—H23*A*⋯Au1	0.98	2.86	3.449 (3)	120
C22—H22*C*⋯Br1^ii^	0.98	2.88	3.851 (3)	173
C12—H12*A*⋯Br1^iii^	0.98	3.02	3.793 (3)	137
C32—H32*A*⋯Se1	0.98	2.95	3.442 (3)	112
C23—H23*A*⋯Se1	0.98	3.03	3.556 (3)	115
C13—H13*A*⋯Se1	0.98	2.70	3.263 (3)	117

Symmetry codes: (ii) 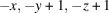; (iii) 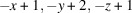.

**Table:** 

	**1**	**2**	**3**	**4a**
Crystal data				
Chemical formula	[Au(C_9_H_21_PS)_2_][AuCl_4_]	[C_20_H_46_AuP_2_S_2_][;AuCl_4_]	[Au(C_12_H_27_PS)_2_][AuCl_4_]	[C_20_H_46_AuP_2_S_2_][AuBr_4_]
*M*_r_	920.31	948.36	1004.46	1126.20
Crystal system, space group	Monoclinic, *P*2/*n*	Triclinic, *P* 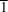	Triclinic, *P* 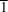	Monoclinic, *P*2_1_/*c*
Temperature (K)	100	100	100	100
*a*, *b*, *c* (Å)	14.2552 (4), 9.0574 (2), 23.0043 (7)	11.7607 (3), 16.4174 (4), 17.2173 (4)	8.5541 (2), 9.1550 (3), 12.0421 (4)	13.7871 (4), 10.4042 (3), 22.7240 (6)
α, β, γ (°)	90, 96.703 (3), 90	79.931 (2), 76.467 (2), 78.015 (2)	107.427 (3), 97.511 (3), 102.841 (3)	90, 93.035 (3), 90
*V* (Å^3^)	2949.89 (14)	3133.57 (14)	857.30 (5)	3255.05 (16)
*Z*	4	4	1	4
Radiation type	Mo *K*α	Mo *K*α	Mo *K*α	Mo *K*α
μ (mm^−1^)	10.55	9.94	9.09	14.15
Crystal size (mm)	0.2 × 0.1 × 0.01	0.2 × 0.1 × 0.03	0.2 × 0.15 × 0.15	0.25 × 0.15 × 0.02

Data collection				
Diffractometer	Oxford Diffraction Xcalibur, Eos	Oxford Diffraction Xcalibur, Eos	Oxford Diffraction Xcalibur, Eos	Oxford Diffraction Xcalibur, Eos
Absorption correction	Multi-scan (*CrysAlis PRO*; Rigaku OD, 2015 ▸)	Multi-scan (*CrysAlis PRO*; Rigaku OD, 2015 ▸)	Multi-scan (*CrysAlis PRO*; Rigaku OD, 2015 ▸)	Multi-scan (*CrysAlis PRO*; Rigaku OD, 2015 ▸)
*T*_min_, *T*_max_	0.352, 1.000	0.439, 1.000	0.623, 1.000	0.179, 1.000
No. of measured, independent and observed [*I* > 2σ(*I*)] reflections	79388, 8561, 6768	179513, 18142, 14554	166022, 5209, 4622	169756, 6651, 5373
*R*_int_	0.067	0.062	0.043	0.086
θ values (°)	θ_max_ = 30.0, θ_min_ = 2.3	θ_max_ = 30.0, θ_min_ = 2.4	θ_max_ = 31.1, θ_min_ = 2.4	θ_max_ = 26.4, θ_min_ = 2.2
(sin θ/λ)_max_ (Å^−1^)	0.704	0.704	0.726	0.625

Refinement				
*R*[*F*^2^ > 2σ(*F*^2^)], *wR*(*F*^2^), *S*	0.028, 0.049, 1.05	0.030, 0.055, 1.05	0.016, 0.038, 1.09	0.046, 0.112, 1.02
No. of reflections	8561	18142	5209	6651
No. of parameters	269	569	167	288
H-atom treatment	H-atom parameters constrained	H-atom parameters constrained	H-atom parameters constrained	H-atom parameters constrained
Δρ_max_, Δρ_min_ (e Å^−3^)	1.88, −1.42	1.76, −1.51	1.02, −1.38	4.29, −2.02
Extinction method	None	None	*SHELXL2019/3* (Sheldrick, 2015 ▸), *F*_c_^*^ = *kF*_c_[1 + 0.001 × *F*_c_^2^λ^3^/sin(2θ)]^-1/4^	None
Extinction coefficient	–	–	0.00495 (18)	–

**Table:** 

	**4b**	**5a**	**5b**
Crystal data			
Chemical formula	[Au(C_10_H_23_PSe)_2_][AuBr_4_]	[Au(C_12_H_27_PS)_2_][AuBr_4_]	[Au(C_12_H_27_PSe)_2_][AuBr_4_]
*M*_r_	1220.00	1182.30	1276.10
Crystal system, space group	Monoclinic, *P*2_1_/*c*	Triclinic, *P* 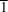	Triclinic, *P* 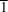
Temperature (K)	100	100	100
*a*, *b*, *c* (Å)	13.7265 (3), 10.5615 (3), 22.7782 (5)	8.4858 (4), 9.3738 (4), 11.9910 (5)	8.4403 (4), 9.2135 (4), 12.6496 (5)
α, β, γ (°)	90, 94.096 (2), 90	105.533 (4), 97.476 (4), 99.318 (4)	106.172 (4), 101.100 (4), 97.485 (4)
*V* (Å^3^)	3293.78 (14)	891.63 (7)	909.28 (7)
*Z*	4	1	1
Radiation type	Mo *K*α	Mo *K*α	Mo *K*α
μ (mm^−1^)	16.07	12.92	14.56
Crystal size (mm)	0.15 × 0.10 × 0.05	0.12 × 0.12 × 0.08	0.12 × 0.12 × 0.04

Data collection			
Diffractometer	Oxford Diffraction Xcalibur, Eos	Oxford Diffraction Xcalibur, Eos	Oxford Diffraction Xcalibur, Eos
Absorption correction	Multi-scan (*CrysAlis PRO*; Rigaku OD, 2015 ▸)	Multi-scan (*CrysAlis PRO*; Rigaku OD, 2015 ▸)	Multi-scan (*CrysAlis PRO*; Rigaku OD, 2015 ▸)
*T*_min_, *T*_max_	0.589, 1.000	0.765, 1.000	0.468, 1.000
No. of measured, independent and observed [*I* > 2σ(*I*)] reflections	106256, 9556, 7328	47038, 5273, 4754	48315, 5398, 4704
*R*_int_	0.068	0.035	0.039
θ values (°)	θ_max_ = 30.0, θ_min_ = 2.1	θ_max_ = 30.7, θ_min_ = 2.3	θ_max_ = 30.9, θ_min_ = 2.4
(sin θ/λ)_max_ (Å^−1^)	0.704	0.719	0.722

Refinement			
*R*[*F*^2^ > 2σ(*F*^2^)], *wR*(*F*^2^), *S*	0.030, 0.049, 1.03	0.017, 0.033, 1.06	0.020, 0.036, 1.06
No. of reflections	9556	5273	5398
No. of parameters	288	167	166
H-atom treatment	H-atom parameters constrained	H-atom parameters constrained	H-atom parameters constrained
Δρ_max_, Δρ_min_ (e Å^−3^)	1.57, −1.09	0.73, −0.71	0.83, −0.88
Extinction method	None	*SHELXL2019/3* (Sheldrick, 2015 ▸), *F*_c_^*^ = *kF*_c_[1 + 0.001 × *F*_c_^2^λ^3^/sin(2θ)]^-1/4^	None
Extinction coefficient	–	0.00106 (8)	–

Computer programs: *CrysAlis PRO* (Rigaku OD, 2015 ▸), *SHELXS97* (Sheldrick, 2008 ▸), *SHELXL2019/3* (Sheldrick, 2015 ▸), *XP* (Bruker, 1998 ▸) and *publCIF* (Westrip, 2010 ▸).

## supplementary crystallographic information

### Bis[tris(propan-2-yl)-λ5-phosphanethione-κS]gold(I) tetrachloridoaurate(III) (1) Crystal data

**Table:**

[Au(C_9_H_21_PS)_2_][AuCl_4_]	*F*(000) = 1752
*M**_r_* = 920.31	*D*_x_ = 2.072 Mg m^−^^3^
Monoclinic, *P*2/*n*	Mo *K*α radiation, λ = 0.71073 Å
*a* = 14.2552 (4) Å	Cell parameters from 13693 reflections
*b* = 9.0574 (2) Å	θ = 2.2–30.9°
*c* = 23.0043 (7) Å	µ = 10.55 mm^−^^1^
β = 96.703 (3)°	*T* = 100 K
*V* = 2949.89 (14) Å^3^	Plate, yellow
*Z* = 4	0.2 × 0.1 × 0.01 mm

### Bis[tris(propan-2-yl)-λ5-phosphanethione-κS]gold(I) tetrachloridoaurate(III) (1) Data collection

**Table:**

Oxford Diffraction Xcalibur, Eos diffractometer	8561 independent reflections
Radiation source: fine-focus sealed tube	6768 reflections with *I* > 2σ(*I*)
Detector resolution: 16.1419 pixels mm^-1^	*R*_int_ = 0.067
ω scans	θ_max_ = 30.0°, θ_min_ = 2.3°
Absorption correction: multi-scan (CrysAlisPro; Rigaku OD, 2015)	*h* = −20→19
*T*_min_ = 0.352, *T*_max_ = 1.000	*k* = −12→12
79388 measured reflections	*l* = −32→32

### Bis[tris(propan-2-yl)-λ5-phosphanethione-κS]gold(I) tetrachloridoaurate(III) (1) Refinement

**Table:**

Refinement on *F*^2^	Primary atom site location: structure-invariant direct methods
Least-squares matrix: full	Secondary atom site location: difference Fourier map
*R*[*F*^2^ > 2σ(*F*^2^)] = 0.028	Hydrogen site location: inferred from neighbouring sites
*wR*(*F*^2^) = 0.049	H-atom parameters constrained
*S* = 1.05	*w* = 1/[σ^2^(*F*_o_^2^) + (0.0115*P*)^2^ + 3.8854*P*] where *P* = (*F*_o_^2^ + 2*F*_c_^2^)/3
8561 reflections	(Δ/σ)_max_ = 0.001
269 parameters	Δρ_max_ = 1.88 e Å^−^^3^
0 restraints	Δρ_min_ = −1.42 e Å^−^^3^

### Bis[tris(propan-2-yl)-λ5-phosphanethione-κS]gold(I) tetrachloridoaurate(III) (1) Fractional atomic coordinates and isotropic or equivalent isotropic displacement parameters (Å^2^)

**Table:**

	*x*	*y*	*z*	*U*_iso_*/*U*_eq_	
Au1	0.250000	0.16205 (2)	0.750000	0.01848 (5)	
P1	0.40748 (7)	0.10509 (10)	0.65390 (4)	0.0155 (2)	
S1	0.40795 (7)	0.16199 (11)	0.73960 (4)	0.0205 (2)	
C1	0.5290 (3)	0.1275 (5)	0.6355 (2)	0.0308 (10)	
H1	0.532119	0.228422	0.618203	0.037*	
C2	0.3309 (3)	0.2305 (5)	0.60681 (19)	0.0316 (10)	
H2	0.264765	0.205070	0.613560	0.038*	
C3	0.3638 (3)	−0.0800 (4)	0.63756 (17)	0.0231 (9)	
H3	0.375877	−0.102080	0.596482	0.028*	
C11	0.5527 (3)	0.0174 (5)	0.58739 (19)	0.0306 (10)	
H11A	0.613911	0.043213	0.574737	0.046*	
H11B	0.503597	0.022832	0.553879	0.046*	
H11C	0.555460	−0.083109	0.603246	0.046*	
C12	0.6032 (3)	0.1233 (6)	0.6871 (2)	0.0399 (12)	
H12A	0.602198	0.026748	0.706203	0.060*	
H12B	0.590623	0.200846	0.714857	0.060*	
H12C	0.665336	0.139608	0.673958	0.060*	
C21	0.3437 (3)	0.3888 (5)	0.6232 (2)	0.0361 (12)	
H21A	0.408757	0.418712	0.619453	0.054*	
H21B	0.330692	0.402695	0.663722	0.054*	
H21C	0.299932	0.449242	0.597113	0.054*	
C22	0.3374 (3)	0.1980 (5)	0.54151 (17)	0.0290 (10)	
H22A	0.292393	0.260521	0.517277	0.044*	
H22B	0.322461	0.093894	0.533345	0.044*	
H22C	0.401610	0.218923	0.532475	0.044*	
C31	0.4205 (4)	−0.1933 (5)	0.67700 (19)	0.0393 (12)	
H31A	0.410246	−0.176105	0.717843	0.059*	
H31B	0.487841	−0.182985	0.672941	0.059*	
H31C	0.399513	−0.293202	0.665392	0.059*	
C32	0.2585 (3)	−0.0991 (5)	0.6398 (2)	0.0382 (12)	
H32A	0.240526	−0.201437	0.630085	0.057*	
H32B	0.223674	−0.032412	0.611456	0.057*	
H32C	0.243262	−0.075910	0.679211	0.057*	
Au2	0.250000	0.42020 (2)	0.250000	0.01671 (5)	
P2	0.08159 (7)	0.41223 (10)	0.34215 (4)	0.01334 (18)	
S2	0.08998 (7)	0.42528 (11)	0.25477 (4)	0.01859 (19)	
C4	−0.0376 (3)	0.4736 (4)	0.35505 (16)	0.0171 (8)	
H4	−0.033456	0.583258	0.359869	0.020*	
C5	0.1682 (3)	0.5331 (4)	0.38310 (16)	0.0172 (8)	
H5	0.231986	0.495079	0.376483	0.021*	
C6	0.1028 (3)	0.2246 (4)	0.37055 (16)	0.0182 (8)	
H6	0.090265	0.225372	0.412414	0.022*	
C41	−0.0681 (3)	0.4139 (4)	0.41232 (17)	0.0214 (8)	
H41A	−0.126748	0.462489	0.420171	0.032*	
H41B	−0.018520	0.433737	0.444578	0.032*	
H41C	−0.078608	0.307132	0.408812	0.032*	
C42	−0.1140 (3)	0.4461 (4)	0.30340 (17)	0.0215 (8)	
H42A	−0.122633	0.339554	0.297532	0.032*	
H42B	−0.094417	0.490538	0.267886	0.032*	
H42C	−0.173578	0.490530	0.311818	0.032*	
C51	0.1621 (3)	0.6919 (4)	0.35998 (18)	0.0257 (9)	
H51A	0.100251	0.733540	0.365331	0.038*	
H51B	0.170246	0.692107	0.318262	0.038*	
H51C	0.211915	0.751577	0.381555	0.038*	
C52	0.1636 (3)	0.5282 (4)	0.44935 (16)	0.0201 (8)	
H52A	0.218437	0.579700	0.469577	0.030*	
H52B	0.163846	0.425200	0.462415	0.030*	
H52C	0.105526	0.576554	0.458390	0.030*	
C61	0.0345 (3)	0.1131 (4)	0.33827 (18)	0.0224 (9)	
H61A	0.045051	0.109188	0.296955	0.034*	
H61B	−0.030649	0.143660	0.341327	0.034*	
H61C	0.045569	0.015228	0.355907	0.034*	
C62	0.2058 (3)	0.1760 (4)	0.36953 (19)	0.0273 (9)	
H62A	0.214190	0.076285	0.385846	0.041*	
H62B	0.247746	0.244578	0.393013	0.041*	
H62C	0.221056	0.176091	0.329094	0.041*	
Au3	0.500000	0.500000	0.500000	0.01729 (5)	
Cl1	0.42083 (7)	0.44491 (11)	0.41040 (4)	0.0263 (2)	
Cl2	0.39843 (7)	0.69109 (11)	0.50949 (5)	0.0276 (2)	
Au4	0.000000	0.000000	0.500000	0.01674 (5)	
Cl3	−0.14756 (7)	0.08430 (11)	0.51132 (5)	0.0264 (2)	
Cl4	0.06567 (7)	0.19959 (11)	0.55010 (5)	0.0282 (2)	

### Bis[tris(propan-2-yl)-λ5-phosphanethione-κS]gold(I) tetrachloridoaurate(III) (1) Atomic displacement parameters (Å^2^)

**Table:**

	*U*^11^	*U*^22^	*U*^33^	*U*^12^	*U*^13^	*U*^23^
Au1	0.01698 (11)	0.02131 (11)	0.01830 (11)	0.000	0.00687 (8)	0.000
P1	0.0137 (5)	0.0176 (5)	0.0158 (5)	−0.0032 (4)	0.0039 (4)	0.0001 (4)
S1	0.0168 (5)	0.0270 (5)	0.0183 (5)	−0.0012 (4)	0.0044 (4)	−0.0043 (4)
C1	0.024 (2)	0.037 (3)	0.033 (3)	−0.0023 (18)	0.008 (2)	−0.0043 (19)
C2	0.028 (2)	0.031 (2)	0.035 (3)	0.0010 (19)	0.003 (2)	0.010 (2)
C3	0.031 (2)	0.020 (2)	0.018 (2)	−0.0065 (18)	0.0019 (17)	−0.0022 (17)
C11	0.020 (2)	0.044 (3)	0.029 (2)	0.0002 (19)	0.0101 (18)	−0.011 (2)
C12	0.018 (2)	0.065 (3)	0.036 (3)	0.001 (2)	0.003 (2)	−0.011 (2)
C21	0.037 (3)	0.032 (2)	0.040 (3)	0.017 (2)	0.010 (2)	0.012 (2)
C22	0.025 (2)	0.035 (2)	0.025 (2)	−0.0068 (19)	−0.0020 (18)	0.0120 (19)
C31	0.071 (4)	0.019 (2)	0.028 (3)	0.005 (2)	0.007 (2)	0.0081 (19)
C32	0.039 (3)	0.047 (3)	0.029 (3)	−0.027 (2)	0.005 (2)	−0.005 (2)
Au2	0.01542 (11)	0.02050 (10)	0.01564 (11)	0.000	0.00788 (8)	0.000
P2	0.0125 (5)	0.0151 (4)	0.0130 (5)	−0.0004 (4)	0.0039 (4)	−0.0005 (4)
S2	0.0157 (5)	0.0266 (5)	0.0143 (5)	−0.0014 (4)	0.0051 (4)	0.0008 (4)
C4	0.0147 (19)	0.0197 (19)	0.018 (2)	0.0010 (14)	0.0084 (15)	−0.0035 (15)
C5	0.0127 (19)	0.0198 (19)	0.019 (2)	−0.0028 (14)	0.0022 (15)	−0.0022 (14)
C6	0.019 (2)	0.0180 (19)	0.018 (2)	0.0008 (15)	0.0025 (15)	0.0017 (15)
C41	0.015 (2)	0.030 (2)	0.020 (2)	−0.0040 (17)	0.0068 (16)	−0.0021 (17)
C42	0.0113 (19)	0.029 (2)	0.025 (2)	0.0030 (15)	0.0047 (16)	0.0020 (17)
C51	0.031 (2)	0.019 (2)	0.028 (2)	−0.0097 (17)	0.0075 (18)	−0.0045 (17)
C52	0.021 (2)	0.022 (2)	0.017 (2)	−0.0046 (15)	−0.0008 (16)	0.0006 (15)
C61	0.026 (2)	0.0153 (19)	0.025 (2)	−0.0034 (15)	0.0019 (18)	−0.0020 (15)
C62	0.023 (2)	0.022 (2)	0.037 (3)	0.0063 (17)	0.0039 (18)	0.0067 (18)
Au3	0.01436 (10)	0.01801 (10)	0.01919 (11)	−0.00544 (8)	0.00067 (8)	0.00143 (9)
Cl1	0.0208 (5)	0.0341 (5)	0.0228 (5)	−0.0042 (4)	−0.0026 (4)	−0.0027 (4)
Cl2	0.0228 (5)	0.0242 (5)	0.0349 (6)	0.0010 (4)	−0.0006 (4)	−0.0031 (4)
Au4	0.02179 (11)	0.01332 (9)	0.01512 (10)	−0.00296 (8)	0.00222 (8)	−0.00090 (8)
Cl3	0.0234 (5)	0.0247 (5)	0.0311 (6)	−0.0012 (4)	0.0033 (4)	−0.0080 (4)
Cl4	0.0264 (6)	0.0216 (5)	0.0362 (6)	−0.0042 (4)	0.0016 (4)	−0.0121 (4)

### Bis[tris(propan-2-yl)-λ5-phosphanethione-κS]gold(I) tetrachloridoaurate(III) (1) Geometric parameters (Å, º)

**Table:**

Au1—S1	2.2918 (10)	P2—C4	1.844 (4)
Au1—S1^i^	2.2919 (10)	P2—S2	2.0310 (13)
P1—C3	1.812 (4)	C4—C41	1.534 (5)
P1—C2	1.838 (4)	C4—C42	1.536 (5)
P1—C1	1.842 (4)	C4—H4	1.0000
P1—S1	2.0369 (14)	C5—C51	1.532 (5)
C1—C12	1.494 (6)	C5—C52	1.534 (5)
C1—C11	1.556 (6)	C5—H5	1.0000
C1—H1	1.0000	C6—C61	1.533 (5)
C2—C21	1.488 (6)	C6—C62	1.534 (5)
C2—C22	1.544 (6)	C6—H6	1.0000
C2—H2	1.0000	C41—H41A	0.9800
C3—C32	1.519 (6)	C41—H41B	0.9800
C3—C31	1.535 (6)	C41—H41C	0.9800
C3—H3	1.0000	C42—H42A	0.9800
C11—H11A	0.9800	C42—H42B	0.9800
C11—H11B	0.9800	C42—H42C	0.9800
C11—H11C	0.9800	C51—H51A	0.9800
C12—H12A	0.9800	C51—H51B	0.9800
C12—H12B	0.9800	C51—H51C	0.9800
C12—H12C	0.9800	C52—H52A	0.9800
C21—H21A	0.9800	C52—H52B	0.9800
C21—H21B	0.9800	C52—H52C	0.9800
C21—H21C	0.9800	C61—H61A	0.9800
C22—H22A	0.9800	C61—H61B	0.9800
C22—H22B	0.9800	C61—H61C	0.9800
C22—H22C	0.9800	C62—H62A	0.9800
C31—H31A	0.9800	C62—H62B	0.9800
C31—H31B	0.9800	C62—H62C	0.9800
C31—H31C	0.9800	Au3—Cl2	2.2833 (10)
C32—H32A	0.9800	Au3—Cl2^iii^	2.2833 (10)
C32—H32B	0.9800	Au3—Cl1	2.2865 (10)
C32—H32C	0.9800	Au3—Cl1^iii^	2.2865 (10)
Au2—S2^ii^	2.2970 (9)	Au4—Cl3^iv^	2.2811 (10)
Au2—S2	2.2970 (9)	Au4—Cl3	2.2811 (10)
P2—C5	1.828 (4)	Au4—Cl4^iv^	2.2849 (9)
P2—C6	1.833 (4)	Au4—Cl4	2.2849 (9)
			
S1—Au1—S1^i^	179.97 (5)	C4—P2—S2	107.67 (13)
C3—P1—C2	106.5 (2)	P2—S2—Au2	102.69 (5)
C3—P1—C1	111.3 (2)	C41—C4—C42	111.1 (3)
C2—P1—C1	107.8 (2)	C41—C4—P2	113.0 (3)
C3—P1—S1	113.40 (14)	C42—C4—P2	114.2 (2)
C2—P1—S1	110.41 (15)	C41—C4—H4	105.9
C1—P1—S1	107.31 (15)	C42—C4—H4	105.9
P1—S1—Au1	102.05 (5)	P2—C4—H4	105.9
C12—C1—C11	110.9 (4)	C51—C5—C52	111.4 (3)
C12—C1—P1	114.3 (3)	C51—C5—P2	112.1 (3)
C11—C1—P1	112.4 (3)	C52—C5—P2	112.9 (3)
C12—C1—H1	106.2	C51—C5—H5	106.7
C11—C1—H1	106.2	C52—C5—H5	106.7
P1—C1—H1	106.2	P2—C5—H5	106.7
C21—C2—C22	114.2 (4)	C61—C6—C62	111.0 (3)
C21—C2—P1	113.6 (3)	C61—C6—P2	111.6 (3)
C22—C2—P1	110.9 (3)	C62—C6—P2	112.3 (3)
C21—C2—H2	105.8	C61—C6—H6	107.2
C22—C2—H2	105.8	C62—C6—H6	107.2
P1—C2—H2	105.8	P2—C6—H6	107.2
C32—C3—C31	111.0 (4)	C4—C41—H41A	109.5
C32—C3—P1	114.5 (3)	C4—C41—H41B	109.5
C31—C3—P1	110.6 (3)	H41A—C41—H41B	109.5
C32—C3—H3	106.8	C4—C41—H41C	109.5
C31—C3—H3	106.8	H41A—C41—H41C	109.5
P1—C3—H3	106.8	H41B—C41—H41C	109.5
C1—C11—H11A	109.5	C4—C42—H42A	109.5
C1—C11—H11B	109.5	C4—C42—H42B	109.5
H11A—C11—H11B	109.5	H42A—C42—H42B	109.5
C1—C11—H11C	109.5	C4—C42—H42C	109.5
H11A—C11—H11C	109.5	H42A—C42—H42C	109.5
H11B—C11—H11C	109.5	H42B—C42—H42C	109.5
C1—C12—H12A	109.5	C5—C51—H51A	109.5
C1—C12—H12B	109.5	C5—C51—H51B	109.5
H12A—C12—H12B	109.5	H51A—C51—H51B	109.5
C1—C12—H12C	109.5	C5—C51—H51C	109.5
H12A—C12—H12C	109.5	H51A—C51—H51C	109.5
H12B—C12—H12C	109.5	H51B—C51—H51C	109.5
C2—C21—H21A	109.5	C5—C52—H52A	109.5
C2—C21—H21B	109.5	C5—C52—H52B	109.5
H21A—C21—H21B	109.5	H52A—C52—H52B	109.5
C2—C21—H21C	109.5	C5—C52—H52C	109.5
H21A—C21—H21C	109.5	H52A—C52—H52C	109.5
H21B—C21—H21C	109.5	H52B—C52—H52C	109.5
C2—C22—H22A	109.5	C6—C61—H61A	109.5
C2—C22—H22B	109.5	C6—C61—H61B	109.5
H22A—C22—H22B	109.5	H61A—C61—H61B	109.5
C2—C22—H22C	109.5	C6—C61—H61C	109.5
H22A—C22—H22C	109.5	H61A—C61—H61C	109.5
H22B—C22—H22C	109.5	H61B—C61—H61C	109.5
C3—C31—H31A	109.5	C6—C62—H62A	109.5
C3—C31—H31B	109.5	C6—C62—H62B	109.5
H31A—C31—H31B	109.5	H62A—C62—H62B	109.5
C3—C31—H31C	109.5	C6—C62—H62C	109.5
H31A—C31—H31C	109.5	H62A—C62—H62C	109.5
H31B—C31—H31C	109.5	H62B—C62—H62C	109.5
C3—C32—H32A	109.5	Cl2—Au3—Cl2^iii^	180.0
C3—C32—H32B	109.5	Cl2—Au3—Cl1	89.99 (4)
H32A—C32—H32B	109.5	Cl2^iii^—Au3—Cl1	90.01 (4)
C3—C32—H32C	109.5	Cl2—Au3—Cl1^iii^	90.01 (4)
H32A—C32—H32C	109.5	Cl2^iii^—Au3—Cl1^iii^	89.99 (4)
H32B—C32—H32C	109.5	Cl1—Au3—Cl1^iii^	180.0
S2^ii^—Au2—S2	177.70 (5)	Cl3^iv^—Au4—Cl3	180.0
C5—P2—C6	107.35 (17)	Cl3^iv^—Au4—Cl4^iv^	90.37 (4)
C5—P2—C4	108.43 (17)	Cl3—Au4—Cl4^iv^	89.63 (4)
C6—P2—C4	109.83 (17)	Cl3^iv^—Au4—Cl4	89.63 (4)
C5—P2—S2	111.16 (13)	Cl3—Au4—Cl4	90.37 (4)
C6—P2—S2	112.35 (13)	Cl4^iv^—Au4—Cl4	180.0
			
C3—P1—S1—Au1	−65.05 (16)	C5—P2—S2—Au2	43.70 (14)
C2—P1—S1—Au1	54.33 (16)	C6—P2—S2—Au2	−76.62 (14)
C1—P1—S1—Au1	171.60 (15)	C4—P2—S2—Au2	162.32 (12)
C3—P1—C1—C12	−102.6 (4)	C5—P2—C4—C41	−82.6 (3)
C2—P1—C1—C12	141.0 (3)	C6—P2—C4—C41	34.4 (3)
S1—P1—C1—C12	22.0 (4)	S2—P2—C4—C41	157.0 (2)
C3—P1—C1—C11	24.9 (4)	C5—P2—C4—C42	149.1 (3)
C2—P1—C1—C11	−91.5 (3)	C6—P2—C4—C42	−93.9 (3)
S1—P1—C1—C11	149.5 (3)	S2—P2—C4—C42	28.7 (3)
C3—P1—C2—C21	167.8 (3)	C6—P2—C5—C51	175.7 (3)
C1—P1—C2—C21	−72.6 (4)	C4—P2—C5—C51	−65.7 (3)
S1—P1—C2—C21	44.4 (4)	S2—P2—C5—C51	52.5 (3)
C3—P1—C2—C22	−62.0 (3)	C6—P2—C5—C52	−57.5 (3)
C1—P1—C2—C22	57.6 (3)	C4—P2—C5—C52	61.1 (3)
S1—P1—C2—C22	174.5 (2)	S2—P2—C5—C52	179.3 (2)
C2—P1—C3—C32	−51.0 (4)	C5—P2—C6—C61	−179.0 (3)
C1—P1—C3—C32	−168.3 (3)	C4—P2—C6—C61	63.3 (3)
S1—P1—C3—C32	70.6 (3)	S2—P2—C6—C61	−56.5 (3)
C2—P1—C3—C31	−177.3 (3)	C5—P2—C6—C62	−53.7 (3)
C1—P1—C3—C31	65.5 (4)	C4—P2—C6—C62	−171.4 (3)
S1—P1—C3—C31	−55.7 (3)	S2—P2—C6—C62	68.8 (3)

Symmetry codes: (i) −*x*+1/2, *y*, −*z*+3/2; (ii) −*x*+1/2, *y*, −*z*+1/2; (iii) −*x*+1, −*y*+1, −*z*+1; (iv) −*x*, −*y*, −*z*+1.

### Bis[tris(propan-2-yl)-λ5-phosphanethione-κS]gold(I) tetrachloridoaurate(III) (1) Hydrogen-bond geometry (Å, º)

**Table:**

*D*—H···*A*	*D*—H	H···*A*	*D*···*A*	*D*—H···*A*
C32—H32*C*···Au1	0.98	2.70	3.480 (5)	137
C62—H62*C*···Au2	0.98	2.92	3.641 (4)	131
C12—H12*B*···S1	0.98	2.75	3.180 (5)	107
C42—H42*B*···S2	0.98	2.74	3.240 (4)	112
C21—H21*A*···Cl1^iii^	0.98	2.88	3.840 (5)	167
C5—H5···Cl1	1.00	2.75	3.670 (4)	153
C3—H3···Cl2^v^	1.00	2.79	3.682 (4)	149
C52—H52*A*···Cl2	0.98	2.81	3.770 (4)	167
C32—H32*B*···Cl3^iv^	0.98	2.94	3.649 (5)	130
C4—H4···Cl4^vi^	1.00	2.93	3.726 (4)	137
C6—H6···Au4	1.00	3.24	4.022 (4)	136

Symmetry codes: (iii) −*x*+1, −*y*+1, −*z*+1; (iv) −*x*, −*y*, −*z*+1; (v) *x*, *y*−1, *z*; (vi) −*x*, −*y*+1, −*z*+1.

### (2) Crystal data

**Table:**

[C_20_H_46_AuP_2_S_2_][AuCl_4_]	*Z* = 4
*M**_r_* = 948.36	*F*(000) = 1816
Triclinic, *P*1	*D*_x_ = 2.010 Mg m^−^^3^
*a* = 11.7607 (3) Å	Mo *K*α radiation, λ = 0.71073 Å
*b* = 16.4174 (4) Å	Cell parameters from 29113 reflections
*c* = 17.2173 (4) Å	θ = 2.6–29.3°
α = 79.931 (2)°	µ = 9.94 mm^−^^1^
β = 76.467 (2)°	*T* = 100 K
γ = 78.015 (2)°	Irregular, yellow
*V* = 3133.57 (14) Å^3^	0.2 × 0.1 × 0.03 mm

### (2) Data collection

**Table:**

Oxford Diffraction Xcalibur, Eos diffractometer	18142 independent reflections
Radiation source: fine-focus sealed tube	14554 reflections with *I* > 2σ(*I*)
Detector resolution: 16.1419 pixels mm^-1^	*R*_int_ = 0.062
ω scan	θ_max_ = 30.0°, θ_min_ = 2.4°
Absorption correction: multi-scan (CrysAlisPro; Rigaku OD, 2015)	*h* = −16→16
*T*_min_ = 0.439, *T*_max_ = 1.000	*k* = −23→23
179513 measured reflections	*l* = −24→24

### (2) Refinement

**Table:**

Refinement on *F*^2^	Primary atom site location: structure-invariant direct methods
Least-squares matrix: full	Secondary atom site location: difference Fourier map
*R*[*F*^2^ > 2σ(*F*^2^)] = 0.030	Hydrogen site location: inferred from neighbouring sites
*wR*(*F*^2^) = 0.055	H-atom parameters constrained
*S* = 1.05	*w* = 1/[σ^2^(*F*_o_^2^) + (0.0131*P*)^2^ + 6.8476*P*] where *P* = (*F*_o_^2^ + 2*F*_c_^2^)/3
18142 reflections	(Δ/σ)_max_ = 0.003
569 parameters	Δρ_max_ = 1.76 e Å^−^^3^
0 restraints	Δρ_min_ = −1.51 e Å^−^^3^

### (2) Fractional atomic coordinates and isotropic or equivalent isotropic displacement parameters (Å^2^)

**Table:**

	*x*	*y*	*z*	*U*_iso_*/*U*_eq_	
Au1	0.48897 (2)	0.25766 (2)	0.11667 (2)	0.01904 (3)	
P1	0.65776 (9)	0.12888 (6)	0.23566 (5)	0.01599 (19)	
P2	0.32324 (8)	0.37600 (6)	−0.01205 (5)	0.01458 (18)	
S1	0.48357 (9)	0.17782 (6)	0.24001 (6)	0.0223 (2)	
S2	0.49694 (8)	0.33712 (6)	−0.00728 (5)	0.02008 (19)	
C1	0.6636 (4)	0.0667 (2)	0.3368 (2)	0.0218 (8)	
C2	0.7470 (4)	0.2132 (2)	0.2107 (2)	0.0249 (9)	
H2	0.739298	0.238202	0.154450	0.030*	
C3	0.7205 (3)	0.0595 (2)	0.1565 (2)	0.0218 (8)	
H3	0.796910	0.025971	0.169002	0.026*	
C4	0.3193 (3)	0.4617 (2)	−0.0990 (2)	0.0173 (7)	
C5	0.2373 (3)	0.4086 (2)	0.0843 (2)	0.0185 (8)	
H5	0.238463	0.355630	0.123184	0.022*	
C6	0.2555 (3)	0.2910 (2)	−0.0287 (2)	0.0189 (8)	
H6	0.173762	0.316567	−0.037394	0.023*	
C11	0.7849 (4)	0.0125 (3)	0.3374 (3)	0.0330 (10)	
H11A	0.788223	−0.015214	0.392312	0.050*	
H11B	0.846361	0.047727	0.319028	0.050*	
H11C	0.798460	−0.030174	0.301359	0.050*	
C12	0.6438 (5)	0.1290 (3)	0.3984 (3)	0.0513 (16)	
H12A	0.576686	0.173869	0.390043	0.077*	
H12B	0.715499	0.153350	0.390992	0.077*	
H12C	0.626500	0.099208	0.453176	0.077*	
C13	0.5653 (6)	0.0158 (4)	0.3624 (3)	0.0647 (19)	
H13A	0.580882	−0.028721	0.328129	0.097*	
H13B	0.489482	0.052318	0.357070	0.097*	
H13C	0.561548	−0.009454	0.418718	0.097*	
C21	0.6999 (5)	0.2869 (3)	0.2602 (3)	0.0394 (12)	
H21A	0.719734	0.270530	0.313569	0.059*	
H21B	0.613461	0.301665	0.266013	0.059*	
H21C	0.736408	0.335405	0.232470	0.059*	
C22	0.8809 (4)	0.1820 (3)	0.2047 (3)	0.0354 (11)	
H22A	0.924110	0.227828	0.178535	0.053*	
H22B	0.906918	0.135043	0.172786	0.053*	
H22C	0.897101	0.163197	0.258871	0.053*	
C31	0.6414 (5)	−0.0025 (3)	0.1573 (3)	0.0557 (17)	
H31A	0.562335	0.027942	0.151144	0.084*	
H31B	0.634882	−0.040047	0.208461	0.084*	
H31C	0.675727	−0.035745	0.112618	0.084*	
C32	0.7499 (4)	0.1072 (3)	0.0722 (2)	0.0281 (9)	
H32A	0.786250	0.067220	0.033565	0.042*	
H32B	0.805569	0.144478	0.071352	0.042*	
H32C	0.676938	0.140731	0.057536	0.042*	
C41	0.2004 (3)	0.4758 (2)	−0.1253 (2)	0.0212 (8)	
H41A	0.135487	0.488833	−0.079446	0.032*	
H41B	0.192092	0.424833	−0.144179	0.032*	
H41C	0.197517	0.522762	−0.168982	0.032*	
C42	0.3353 (4)	0.5421 (2)	−0.0718 (2)	0.0255 (9)	
H42A	0.346931	0.585296	−0.118628	0.038*	
H42B	0.404837	0.530013	−0.046819	0.038*	
H42C	0.264402	0.562261	−0.032639	0.038*	
C43	0.4226 (4)	0.4404 (3)	−0.1701 (2)	0.0245 (9)	
H43A	0.418038	0.386865	−0.185988	0.037*	
H43B	0.498338	0.435704	−0.153661	0.037*	
H43C	0.417156	0.484922	−0.215714	0.037*	
C51	0.2904 (4)	0.4669 (3)	0.1212 (2)	0.0235 (8)	
H51A	0.282209	0.523054	0.090075	0.035*	
H51B	0.374658	0.444446	0.120056	0.035*	
H51C	0.248157	0.470546	0.177138	0.035*	
C52	0.1062 (3)	0.4419 (3)	0.0846 (2)	0.0234 (8)	
H52A	0.062218	0.444425	0.140235	0.035*	
H52B	0.074902	0.404378	0.059648	0.035*	
H52C	0.097270	0.498308	0.054037	0.035*	
C61	0.3222 (4)	0.2510 (3)	−0.1042 (2)	0.0257 (9)	
H61A	0.406212	0.232869	−0.101671	0.039*	
H61B	0.315043	0.292169	−0.152196	0.039*	
H61C	0.288025	0.202256	−0.107121	0.039*	
C62	0.2419 (4)	0.2228 (3)	0.0441 (2)	0.0301 (10)	
H62A	0.205266	0.179200	0.032166	0.045*	
H62B	0.191409	0.247693	0.090976	0.045*	
H62C	0.320221	0.197724	0.055940	0.045*	
Au2	0.52570 (2)	0.24364 (2)	0.61149 (2)	0.01930 (4)	
P3	0.31820 (9)	0.36332 (6)	0.51182 (5)	0.01685 (19)	
P4	0.72218 (8)	0.13621 (6)	0.73190 (5)	0.01544 (18)	
S3	0.49682 (9)	0.32173 (6)	0.49120 (6)	0.0211 (2)	
S4	0.54537 (8)	0.16443 (6)	0.73283 (6)	0.0218 (2)	
C1'	0.2853 (4)	0.4263 (3)	0.4163 (3)	0.0373 (12)	
C2'	0.2378 (4)	0.2751 (3)	0.5450 (3)	0.0380 (11)	
H2'	0.252865	0.253936	0.600446	0.046*	
C3'	0.2684 (4)	0.4293 (3)	0.5933 (2)	0.0275 (9)	
H3'	0.186042	0.459127	0.590095	0.033*	
C4'	0.7427 (3)	0.0464 (2)	0.8131 (2)	0.0184 (8)	
C5'	0.8066 (3)	0.1136 (2)	0.6315 (2)	0.0183 (7)	
H5'	0.798293	0.168662	0.596019	0.022*	
C6'	0.7780 (3)	0.2252 (2)	0.7523 (2)	0.0202 (8)	
H6'	0.863175	0.205404	0.755815	0.024*	
C11'	0.1651 (4)	0.4848 (3)	0.4305 (3)	0.0367 (11)	
H11D	0.170437	0.530459	0.458597	0.055*	
H11E	0.104696	0.453038	0.463451	0.055*	
H11F	0.143251	0.508320	0.378594	0.055*	
C12'	0.2781 (5)	0.3620 (4)	0.3605 (3)	0.0544 (16)	
H12D	0.260704	0.392602	0.309260	0.082*	
H12E	0.214953	0.329839	0.387207	0.082*	
H12F	0.354198	0.323599	0.350416	0.082*	
C13'	0.3812 (5)	0.4729 (4)	0.3710 (3)	0.0614 (18)	
H13D	0.359083	0.504405	0.321298	0.092*	
H13E	0.455181	0.433099	0.357491	0.092*	
H13F	0.392788	0.512079	0.404245	0.092*	
C21'	0.2811 (6)	0.1994 (3)	0.5013 (4)	0.0657 (18)	
H21D	0.238745	0.153877	0.530006	0.099*	
H21E	0.366506	0.181038	0.499016	0.099*	
H21F	0.266361	0.213757	0.446447	0.099*	
C22'	0.1015 (4)	0.3025 (3)	0.5589 (3)	0.0416 (12)	
H22D	0.077941	0.322226	0.506917	0.062*	
H22E	0.076839	0.347923	0.592604	0.062*	
H22F	0.063127	0.254538	0.586041	0.062*	
C31'	0.3458 (5)	0.4967 (4)	0.5815 (4)	0.068 (2)	
H31D	0.312063	0.534938	0.622057	0.102*	
H31E	0.348268	0.528432	0.527492	0.102*	
H31F	0.426441	0.469793	0.587296	0.102*	
C32'	0.2630 (5)	0.3813 (4)	0.6767 (3)	0.0496 (15)	
H32D	0.343596	0.355874	0.683918	0.074*	
H32E	0.215579	0.337005	0.683586	0.074*	
H32F	0.226339	0.419659	0.716807	0.074*	
C41'	0.8615 (4)	0.0404 (3)	0.8388 (2)	0.0261 (9)	
H41D	0.872739	−0.008814	0.879385	0.039*	
H41E	0.860462	0.091281	0.861679	0.039*	
H41F	0.926805	0.035045	0.791739	0.039*	
C42'	0.7419 (4)	−0.0354 (2)	0.7816 (2)	0.0276 (9)	
H42D	0.738943	−0.081423	0.826405	0.041*	
H42E	0.814144	−0.048362	0.740742	0.041*	
H42F	0.672094	−0.028658	0.757719	0.041*	
C43'	0.6410 (4)	0.0571 (3)	0.8871 (2)	0.0252 (9)	
H43D	0.565266	0.058735	0.871840	0.038*	
H43E	0.639369	0.109708	0.907238	0.038*	
H43F	0.653535	0.009798	0.929376	0.038*	
C51'	0.7593 (4)	0.0546 (3)	0.5922 (2)	0.0247 (9)	
H51D	0.797156	0.057498	0.534931	0.037*	
H51E	0.673156	0.071609	0.597815	0.037*	
H51F	0.777308	−0.003063	0.618711	0.037*	
C52'	0.9404 (3)	0.0867 (3)	0.6284 (2)	0.0231 (8)	
H52D	0.956131	0.029125	0.655723	0.035*	
H52E	0.967510	0.124647	0.655406	0.035*	
H52F	0.982958	0.089005	0.572079	0.035*	
C61'	0.7120 (4)	0.2560 (3)	0.8327 (2)	0.0294 (9)	
H61D	0.736821	0.308121	0.837276	0.044*	
H61E	0.730728	0.213110	0.877341	0.044*	
H61F	0.626197	0.266581	0.834993	0.044*	
C62'	0.7733 (4)	0.2993 (3)	0.6845 (3)	0.0313 (10)	
H62D	0.690635	0.320098	0.679368	0.047*	
H62E	0.819906	0.280639	0.633583	0.047*	
H62F	0.806264	0.344345	0.697038	0.047*	
Au3	0.98348 (2)	0.27544 (2)	0.35919 (2)	0.02001 (4)	
Cl1	1.00561 (11)	0.13536 (7)	0.40405 (6)	0.0336 (2)	
Cl2	0.82031 (10)	0.29765 (8)	0.46001 (6)	0.0347 (3)	
Cl3	0.96111 (10)	0.41602 (7)	0.31367 (7)	0.0354 (3)	
Cl4	1.14644 (10)	0.25137 (7)	0.25917 (6)	0.0316 (2)	
Au4	1.01677 (2)	0.23730 (2)	0.88035 (2)	0.02243 (4)	
Cl5	0.92275 (10)	0.29549 (7)	0.99435 (6)	0.0339 (2)	
Cl6	0.97628 (10)	0.36488 (6)	0.80532 (7)	0.0334 (2)	
Cl7	1.11237 (10)	0.17979 (7)	0.76589 (6)	0.0331 (2)	
Cl8	1.05632 (10)	0.10927 (6)	0.95503 (6)	0.0294 (2)	

### (2) Atomic displacement parameters (Å^2^)

**Table:**

	*U*^11^	*U*^22^	*U*^33^	*U*^12^	*U*^13^	*U*^23^
Au1	0.01852 (7)	0.02126 (7)	0.01813 (7)	0.00208 (6)	−0.00925 (5)	−0.00392 (5)
P1	0.0160 (5)	0.0185 (5)	0.0137 (4)	−0.0016 (4)	−0.0052 (4)	−0.0017 (3)
P2	0.0141 (4)	0.0175 (4)	0.0121 (4)	−0.0018 (4)	−0.0037 (3)	−0.0017 (3)
S1	0.0183 (5)	0.0268 (5)	0.0196 (5)	0.0018 (4)	−0.0056 (4)	−0.0016 (4)
S2	0.0157 (5)	0.0271 (5)	0.0174 (4)	−0.0005 (4)	−0.0057 (3)	−0.0035 (4)
C1	0.022 (2)	0.025 (2)	0.0187 (18)	−0.0033 (16)	−0.0065 (15)	0.0000 (15)
C2	0.035 (2)	0.026 (2)	0.0187 (19)	−0.0108 (18)	−0.0114 (17)	−0.0017 (16)
C3	0.0177 (19)	0.026 (2)	0.0218 (19)	−0.0018 (16)	−0.0014 (15)	−0.0080 (16)
C4	0.0187 (19)	0.0213 (18)	0.0123 (16)	−0.0034 (15)	−0.0057 (14)	0.0006 (14)
C5	0.0188 (19)	0.0214 (18)	0.0138 (17)	0.0004 (15)	−0.0030 (14)	−0.0031 (14)
C6	0.0170 (19)	0.0211 (18)	0.0199 (18)	−0.0044 (15)	−0.0034 (15)	−0.0049 (15)
C11	0.032 (2)	0.031 (2)	0.029 (2)	0.0069 (19)	−0.0100 (19)	0.0055 (18)
C12	0.079 (4)	0.046 (3)	0.019 (2)	0.025 (3)	−0.017 (2)	−0.009 (2)
C13	0.070 (4)	0.083 (4)	0.050 (3)	−0.048 (4)	−0.036 (3)	0.042 (3)
C21	0.059 (3)	0.032 (2)	0.035 (3)	−0.016 (2)	−0.016 (2)	−0.007 (2)
C22	0.033 (3)	0.047 (3)	0.032 (2)	−0.021 (2)	−0.017 (2)	0.008 (2)
C31	0.051 (3)	0.052 (3)	0.069 (4)	−0.034 (3)	0.023 (3)	−0.042 (3)
C32	0.033 (2)	0.034 (2)	0.0164 (19)	−0.0012 (19)	−0.0044 (17)	−0.0073 (17)
C41	0.020 (2)	0.027 (2)	0.0159 (18)	−0.0025 (16)	−0.0070 (15)	0.0006 (15)
C42	0.033 (2)	0.022 (2)	0.022 (2)	−0.0082 (17)	−0.0083 (17)	0.0010 (16)
C43	0.025 (2)	0.033 (2)	0.0160 (18)	−0.0084 (18)	−0.0043 (16)	−0.0004 (16)
C51	0.026 (2)	0.030 (2)	0.0157 (18)	−0.0036 (17)	−0.0057 (16)	−0.0082 (16)
C52	0.021 (2)	0.031 (2)	0.0171 (18)	−0.0020 (17)	−0.0019 (15)	−0.0066 (16)
C61	0.029 (2)	0.028 (2)	0.023 (2)	−0.0073 (18)	−0.0023 (17)	−0.0107 (17)
C62	0.042 (3)	0.030 (2)	0.023 (2)	−0.017 (2)	−0.0086 (19)	−0.0006 (17)
Au2	0.01677 (7)	0.01970 (7)	0.02024 (7)	0.00131 (5)	−0.00621 (5)	−0.00155 (5)
P3	0.0174 (5)	0.0165 (4)	0.0150 (4)	−0.0009 (4)	−0.0037 (4)	0.0003 (4)
P4	0.0159 (5)	0.0156 (4)	0.0146 (4)	−0.0020 (4)	−0.0045 (4)	−0.0004 (3)
S3	0.0177 (5)	0.0217 (5)	0.0192 (4)	0.0031 (4)	−0.0020 (4)	−0.0004 (4)
S4	0.0154 (5)	0.0291 (5)	0.0191 (5)	−0.0022 (4)	−0.0044 (4)	0.0009 (4)
C1'	0.031 (3)	0.039 (3)	0.029 (2)	0.010 (2)	−0.0075 (19)	0.013 (2)
C2'	0.039 (3)	0.026 (2)	0.054 (3)	−0.015 (2)	−0.014 (2)	0.000 (2)
C3'	0.018 (2)	0.034 (2)	0.033 (2)	−0.0010 (17)	−0.0034 (17)	−0.0173 (19)
C4'	0.023 (2)	0.0165 (17)	0.0158 (17)	−0.0067 (15)	−0.0071 (15)	0.0044 (14)
C5'	0.0180 (19)	0.0230 (19)	0.0122 (16)	−0.0020 (15)	−0.0010 (14)	−0.0024 (14)
C6'	0.020 (2)	0.0201 (19)	0.0197 (18)	−0.0052 (16)	−0.0015 (15)	−0.0033 (15)
C11'	0.025 (2)	0.035 (2)	0.041 (3)	0.0050 (19)	−0.011 (2)	0.011 (2)
C12'	0.070 (4)	0.065 (4)	0.026 (3)	0.011 (3)	−0.020 (3)	−0.012 (2)
C13'	0.038 (3)	0.065 (4)	0.056 (3)	−0.004 (3)	−0.002 (3)	0.039 (3)
C21'	0.073 (5)	0.026 (3)	0.103 (5)	−0.010 (3)	−0.027 (4)	−0.010 (3)
C22'	0.031 (3)	0.047 (3)	0.051 (3)	−0.017 (2)	−0.016 (2)	0.006 (2)
C31'	0.038 (3)	0.060 (4)	0.118 (6)	−0.013 (3)	0.001 (3)	−0.062 (4)
C32'	0.040 (3)	0.077 (4)	0.028 (2)	0.017 (3)	−0.012 (2)	−0.024 (3)
C41'	0.025 (2)	0.031 (2)	0.023 (2)	−0.0039 (17)	−0.0111 (16)	0.0039 (17)
C42'	0.039 (3)	0.0195 (19)	0.025 (2)	−0.0094 (18)	−0.0082 (18)	0.0031 (16)
C43'	0.027 (2)	0.033 (2)	0.0171 (19)	−0.0104 (18)	−0.0053 (16)	0.0018 (16)
C51'	0.026 (2)	0.033 (2)	0.0173 (19)	−0.0022 (18)	−0.0087 (16)	−0.0062 (16)
C52'	0.019 (2)	0.032 (2)	0.0187 (19)	−0.0032 (16)	−0.0026 (15)	−0.0062 (16)
C61'	0.029 (2)	0.030 (2)	0.030 (2)	−0.0056 (19)	−0.0004 (18)	−0.0160 (18)
C62'	0.043 (3)	0.019 (2)	0.034 (2)	−0.0115 (19)	−0.012 (2)	0.0027 (17)
Au3	0.02045 (8)	0.02755 (8)	0.01329 (7)	−0.00675 (6)	−0.00314 (5)	−0.00348 (5)
Cl1	0.0455 (7)	0.0283 (5)	0.0255 (5)	−0.0100 (5)	−0.0038 (5)	−0.0001 (4)
Cl2	0.0279 (6)	0.0493 (7)	0.0205 (5)	−0.0034 (5)	0.0021 (4)	−0.0010 (4)
Cl3	0.0357 (6)	0.0284 (5)	0.0331 (6)	−0.0003 (5)	0.0036 (5)	−0.0008 (4)
Cl4	0.0334 (6)	0.0332 (5)	0.0252 (5)	−0.0097 (5)	0.0078 (4)	−0.0099 (4)
Au4	0.02084 (8)	0.02208 (7)	0.02728 (8)	−0.00589 (6)	−0.00502 (6)	−0.00837 (6)
Cl5	0.0346 (6)	0.0333 (6)	0.0330 (6)	−0.0063 (5)	0.0015 (5)	−0.0132 (5)
Cl6	0.0352 (6)	0.0232 (5)	0.0406 (6)	−0.0051 (4)	−0.0055 (5)	−0.0039 (4)
Cl7	0.0418 (7)	0.0307 (5)	0.0279 (5)	−0.0042 (5)	−0.0055 (5)	−0.0118 (4)
Cl8	0.0304 (6)	0.0267 (5)	0.0319 (5)	−0.0052 (4)	−0.0084 (4)	−0.0035 (4)

### (2) Geometric parameters (Å, º)

**Table:**

Au1—S1	2.2869 (9)	P3—C1'	1.860 (4)
Au1—S2	2.2910 (9)	P3—S3	2.0360 (14)
P1—C2	1.840 (4)	P4—C6'	1.837 (4)
P1—C3	1.850 (4)	P4—C5'	1.838 (4)
P1—C1	1.867 (4)	P4—C4'	1.865 (3)
P1—S1	2.0283 (14)	P4—S4	2.0312 (13)
P2—C5	1.834 (4)	C1'—C13'	1.485 (7)
P2—C6	1.837 (4)	C1'—C11'	1.527 (6)
P2—C4	1.869 (3)	C1'—C12'	1.573 (7)
P2—S2	2.0263 (13)	C2'—C21'	1.503 (7)
C1—C13	1.505 (6)	C2'—C22'	1.546 (7)
C1—C11	1.519 (5)	C2'—H2'	1.0000
C1—C12	1.545 (6)	C3'—C32'	1.507 (6)
C2—C21	1.535 (6)	C3'—C31'	1.531 (7)
C2—C22	1.537 (6)	C3'—H3'	1.0000
C2—H2	1.0000	C4'—C42'	1.536 (5)
C3—C31	1.512 (6)	C4'—C43'	1.537 (5)
C3—C32	1.523 (5)	C4'—C41'	1.541 (5)
C3—H3	1.0000	C5'—C51'	1.527 (5)
C4—C41	1.529 (5)	C5'—C52'	1.534 (5)
C4—C42	1.534 (5)	C5'—H5'	1.0000
C4—C43	1.540 (5)	C6'—C62'	1.533 (5)
C5—C52	1.524 (5)	C6'—C61'	1.535 (5)
C5—C51	1.535 (5)	C6'—H6'	1.0000
C5—H5	1.0000	C11'—H11D	0.9800
C6—C62	1.531 (5)	C11'—H11E	0.9800
C6—C61	1.531 (5)	C11'—H11F	0.9800
C6—H6	1.0000	C12'—H12D	0.9800
C11—H11A	0.9800	C12'—H12E	0.9800
C11—H11B	0.9800	C12'—H12F	0.9800
C11—H11C	0.9800	C13'—H13D	0.9800
C12—H12A	0.9800	C13'—H13E	0.9800
C12—H12B	0.9800	C13'—H13F	0.9800
C12—H12C	0.9800	C21'—H21D	0.9800
C13—H13A	0.9800	C21'—H21E	0.9800
C13—H13B	0.9800	C21'—H21F	0.9800
C13—H13C	0.9800	C22'—H22D	0.9800
C21—H21A	0.9800	C22'—H22E	0.9800
C21—H21B	0.9800	C22'—H22F	0.9800
C21—H21C	0.9800	C31'—H31D	0.9800
C22—H22A	0.9800	C31'—H31E	0.9800
C22—H22B	0.9800	C31'—H31F	0.9800
C22—H22C	0.9800	C32'—H32D	0.9800
C31—H31A	0.9800	C32'—H32E	0.9800
C31—H31B	0.9800	C32'—H32F	0.9800
C31—H31C	0.9800	C41'—H41D	0.9800
C32—H32A	0.9800	C41'—H41E	0.9800
C32—H32B	0.9800	C41'—H41F	0.9800
C32—H32C	0.9800	C42'—H42D	0.9800
C41—H41A	0.9800	C42'—H42E	0.9800
C41—H41B	0.9800	C42'—H42F	0.9800
C41—H41C	0.9800	C43'—H43D	0.9800
C42—H42A	0.9800	C43'—H43E	0.9800
C42—H42B	0.9800	C43'—H43F	0.9800
C42—H42C	0.9800	C51'—H51D	0.9800
C43—H43A	0.9800	C51'—H51E	0.9800
C43—H43B	0.9800	C51'—H51F	0.9800
C43—H43C	0.9800	C52'—H52D	0.9800
C51—H51A	0.9800	C52'—H52E	0.9800
C51—H51B	0.9800	C52'—H52F	0.9800
C51—H51C	0.9800	C61'—H61D	0.9800
C52—H52A	0.9800	C61'—H61E	0.9800
C52—H52B	0.9800	C61'—H61F	0.9800
C52—H52C	0.9800	C62'—H62D	0.9800
C61—H61A	0.9800	C62'—H62E	0.9800
C61—H61B	0.9800	C62'—H62F	0.9800
C61—H61C	0.9800	Au3—Cl4	2.2746 (10)
C62—H62A	0.9800	Au3—Cl1	2.2754 (11)
C62—H62B	0.9800	Au3—Cl2	2.2805 (10)
C62—H62C	0.9800	Au3—Cl3	2.2852 (11)
Au2—S4	2.2935 (9)	Au4—Cl5	2.2780 (10)
Au2—S3	2.2953 (9)	Au4—Cl6	2.2825 (11)
P3—C2'	1.828 (4)	Au4—Cl7	2.2828 (10)
P3—C3'	1.835 (4)	Au4—Cl8	2.2839 (10)
			
S1—Au1—S2	179.28 (4)	C1'—P3—S3	106.48 (15)
C2—P1—C3	105.43 (18)	C6'—P4—C5'	105.38 (17)
C2—P1—C1	113.57 (18)	C6'—P4—C4'	109.30 (17)
C3—P1—C1	109.59 (18)	C5'—P4—C4'	113.54 (17)
C2—P1—S1	110.40 (14)	C6'—P4—S4	111.80 (13)
C3—P1—S1	112.92 (13)	C5'—P4—S4	110.47 (13)
C1—P1—S1	105.10 (13)	C4'—P4—S4	106.46 (13)
C5—P2—C6	105.27 (17)	P3—S3—Au2	103.22 (5)
C5—P2—C4	113.74 (17)	P4—S4—Au2	106.42 (5)
C6—P2—C4	109.42 (17)	C13'—C1'—C11'	111.4 (4)
C5—P2—S2	110.69 (12)	C13'—C1'—C12'	105.8 (4)
C6—P2—S2	111.89 (13)	C11'—C1'—C12'	107.7 (4)
C4—P2—S2	105.95 (12)	C13'—C1'—P3	113.3 (4)
P1—S1—Au1	101.88 (5)	C11'—C1'—P3	111.6 (3)
P2—S2—Au1	102.91 (5)	C12'—C1'—P3	106.6 (3)
C13—C1—C11	112.1 (4)	C21'—C2'—C22'	112.6 (4)
C13—C1—C12	107.8 (4)	C21'—C2'—P3	117.8 (4)
C11—C1—C12	107.3 (4)	C22'—C2'—P3	113.2 (3)
C13—C1—P1	110.4 (3)	C21'—C2'—H2'	103.7
C11—C1—P1	111.1 (3)	C22'—C2'—H2'	103.7
C12—C1—P1	108.1 (3)	P3—C2'—H2'	103.7
C21—C2—C22	112.3 (4)	C32'—C3'—C31'	109.5 (4)
C21—C2—P1	115.4 (3)	C32'—C3'—P3	114.3 (3)
C22—C2—P1	114.2 (3)	C31'—C3'—P3	111.1 (3)
C21—C2—H2	104.5	C32'—C3'—H3'	107.2
C22—C2—H2	104.5	C31'—C3'—H3'	107.2
P1—C2—H2	104.5	P3—C3'—H3'	107.2
C31—C3—C32	110.0 (4)	C42'—C4'—C43'	108.5 (3)
C31—C3—P1	112.3 (3)	C42'—C4'—C41'	109.3 (3)
C32—C3—P1	113.4 (3)	C43'—C4'—C41'	108.8 (3)
C31—C3—H3	106.9	C42'—C4'—P4	109.0 (2)
C32—C3—H3	106.9	C43'—C4'—P4	110.5 (3)
P1—C3—H3	106.9	C41'—C4'—P4	110.6 (3)
C41—C4—C42	110.0 (3)	C51'—C5'—C52'	111.5 (3)
C41—C4—C43	110.3 (3)	C51'—C5'—P4	115.9 (3)
C42—C4—C43	107.7 (3)	C52'—C5'—P4	112.9 (2)
C41—C4—P2	110.0 (3)	C51'—C5'—H5'	105.2
C42—C4—P2	108.1 (2)	C52'—C5'—H5'	105.2
C43—C4—P2	110.7 (3)	P4—C5'—H5'	105.2
C52—C5—C51	111.0 (3)	C62'—C6'—C61'	109.3 (3)
C52—C5—P2	113.9 (2)	C62'—C6'—P4	112.4 (3)
C51—C5—P2	116.0 (3)	C61'—C6'—P4	112.6 (3)
C52—C5—H5	104.9	C62'—C6'—H6'	107.5
C51—C5—H5	104.9	C61'—C6'—H6'	107.5
P2—C5—H5	104.9	P4—C6'—H6'	107.5
C62—C6—C61	109.7 (3)	C1'—C11'—H11D	109.5
C62—C6—P2	112.8 (3)	C1'—C11'—H11E	109.5
C61—C6—P2	112.7 (3)	H11D—C11'—H11E	109.5
C62—C6—H6	107.1	C1'—C11'—H11F	109.5
C61—C6—H6	107.1	H11D—C11'—H11F	109.5
P2—C6—H6	107.1	H11E—C11'—H11F	109.5
C1—C11—H11A	109.5	C1'—C12'—H12D	109.5
C1—C11—H11B	109.5	C1'—C12'—H12E	109.5
H11A—C11—H11B	109.5	H12D—C12'—H12E	109.5
C1—C11—H11C	109.5	C1'—C12'—H12F	109.5
H11A—C11—H11C	109.5	H12D—C12'—H12F	109.5
H11B—C11—H11C	109.5	H12E—C12'—H12F	109.5
C1—C12—H12A	109.5	C1'—C13'—H13D	109.5
C1—C12—H12B	109.5	C1'—C13'—H13E	109.5
H12A—C12—H12B	109.5	H13D—C13'—H13E	109.5
C1—C12—H12C	109.5	C1'—C13'—H13F	109.5
H12A—C12—H12C	109.5	H13D—C13'—H13F	109.5
H12B—C12—H12C	109.5	H13E—C13'—H13F	109.5
C1—C13—H13A	109.5	C2'—C21'—H21D	109.5
C1—C13—H13B	109.5	C2'—C21'—H21E	109.5
H13A—C13—H13B	109.5	H21D—C21'—H21E	109.5
C1—C13—H13C	109.5	C2'—C21'—H21F	109.5
H13A—C13—H13C	109.5	H21D—C21'—H21F	109.5
H13B—C13—H13C	109.5	H21E—C21'—H21F	109.5
C2—C21—H21A	109.5	C2'—C22'—H22D	109.5
C2—C21—H21B	109.5	C2'—C22'—H22E	109.5
H21A—C21—H21B	109.5	H22D—C22'—H22E	109.5
C2—C21—H21C	109.5	C2'—C22'—H22F	109.5
H21A—C21—H21C	109.5	H22D—C22'—H22F	109.5
H21B—C21—H21C	109.5	H22E—C22'—H22F	109.5
C2—C22—H22A	109.5	C3'—C31'—H31D	109.5
C2—C22—H22B	109.5	C3'—C31'—H31E	109.5
H22A—C22—H22B	109.5	H31D—C31'—H31E	109.5
C2—C22—H22C	109.5	C3'—C31'—H31F	109.5
H22A—C22—H22C	109.5	H31D—C31'—H31F	109.5
H22B—C22—H22C	109.5	H31E—C31'—H31F	109.5
C3—C31—H31A	109.5	C3'—C32'—H32D	109.5
C3—C31—H31B	109.5	C3'—C32'—H32E	109.5
H31A—C31—H31B	109.5	H32D—C32'—H32E	109.5
C3—C31—H31C	109.5	C3'—C32'—H32F	109.5
H31A—C31—H31C	109.5	H32D—C32'—H32F	109.5
H31B—C31—H31C	109.5	H32E—C32'—H32F	109.5
C3—C32—H32A	109.5	C4'—C41'—H41D	109.5
C3—C32—H32B	109.5	C4'—C41'—H41E	109.5
H32A—C32—H32B	109.5	H41D—C41'—H41E	109.5
C3—C32—H32C	109.5	C4'—C41'—H41F	109.5
H32A—C32—H32C	109.5	H41D—C41'—H41F	109.5
H32B—C32—H32C	109.5	H41E—C41'—H41F	109.5
C4—C41—H41A	109.5	C4'—C42'—H42D	109.5
C4—C41—H41B	109.5	C4'—C42'—H42E	109.5
H41A—C41—H41B	109.5	H42D—C42'—H42E	109.5
C4—C41—H41C	109.5	C4'—C42'—H42F	109.5
H41A—C41—H41C	109.5	H42D—C42'—H42F	109.5
H41B—C41—H41C	109.5	H42E—C42'—H42F	109.5
C4—C42—H42A	109.5	C4'—C43'—H43D	109.5
C4—C42—H42B	109.5	C4'—C43'—H43E	109.5
H42A—C42—H42B	109.5	H43D—C43'—H43E	109.5
C4—C42—H42C	109.5	C4'—C43'—H43F	109.5
H42A—C42—H42C	109.5	H43D—C43'—H43F	109.5
H42B—C42—H42C	109.5	H43E—C43'—H43F	109.5
C4—C43—H43A	109.5	C5'—C51'—H51D	109.5
C4—C43—H43B	109.5	C5'—C51'—H51E	109.5
H43A—C43—H43B	109.5	H51D—C51'—H51E	109.5
C4—C43—H43C	109.5	C5'—C51'—H51F	109.5
H43A—C43—H43C	109.5	H51D—C51'—H51F	109.5
H43B—C43—H43C	109.5	H51E—C51'—H51F	109.5
C5—C51—H51A	109.5	C5'—C52'—H52D	109.5
C5—C51—H51B	109.5	C5'—C52'—H52E	109.5
H51A—C51—H51B	109.5	H52D—C52'—H52E	109.5
C5—C51—H51C	109.5	C5'—C52'—H52F	109.5
H51A—C51—H51C	109.5	H52D—C52'—H52F	109.5
H51B—C51—H51C	109.5	H52E—C52'—H52F	109.5
C5—C52—H52A	109.5	C6'—C61'—H61D	109.5
C5—C52—H52B	109.5	C6'—C61'—H61E	109.5
H52A—C52—H52B	109.5	H61D—C61'—H61E	109.5
C5—C52—H52C	109.5	C6'—C61'—H61F	109.5
H52A—C52—H52C	109.5	H61D—C61'—H61F	109.5
H52B—C52—H52C	109.5	H61E—C61'—H61F	109.5
C6—C61—H61A	109.5	C6'—C62'—H62D	109.5
C6—C61—H61B	109.5	C6'—C62'—H62E	109.5
H61A—C61—H61B	109.5	H62D—C62'—H62E	109.5
C6—C61—H61C	109.5	C6'—C62'—H62F	109.5
H61A—C61—H61C	109.5	H62D—C62'—H62F	109.5
H61B—C61—H61C	109.5	H62E—C62'—H62F	109.5
C6—C62—H62A	109.5	Cl4—Au3—Cl1	89.76 (4)
C6—C62—H62B	109.5	Cl4—Au3—Cl2	179.22 (4)
H62A—C62—H62B	109.5	Cl1—Au3—Cl2	89.46 (4)
C6—C62—H62C	109.5	Cl4—Au3—Cl3	90.14 (4)
H62A—C62—H62C	109.5	Cl1—Au3—Cl3	179.77 (4)
H62B—C62—H62C	109.5	Cl2—Au3—Cl3	90.64 (4)
S4—Au2—S3	177.24 (4)	Cl5—Au4—Cl6	89.61 (4)
C2'—P3—C3'	105.3 (2)	Cl5—Au4—Cl7	179.44 (4)
C2'—P3—C1'	113.0 (2)	Cl6—Au4—Cl7	90.15 (4)
C3'—P3—C1'	109.3 (2)	Cl5—Au4—Cl8	90.54 (4)
C2'—P3—S3	110.71 (16)	Cl6—Au4—Cl8	179.58 (4)
C3'—P3—S3	112.14 (14)	Cl7—Au4—Cl8	89.70 (4)
			
C2—P1—S1—Au1	54.15 (14)	C2'—P3—S3—Au2	−55.89 (18)
C3—P1—S1—Au1	−63.59 (14)	C3'—P3—S3—Au2	61.40 (16)
C1—P1—S1—Au1	176.99 (13)	C1'—P3—S3—Au2	−179.04 (18)
C5—P2—S2—Au1	42.76 (14)	C6'—P4—S4—Au2	78.15 (13)
C6—P2—S2—Au1	−74.31 (13)	C5'—P4—S4—Au2	−38.86 (14)
C4—P2—S2—Au1	166.51 (12)	C4'—P4—S4—Au2	−162.56 (13)
C2—P1—C1—C13	164.8 (4)	C2'—P3—C1'—C13'	−155.7 (4)
C3—P1—C1—C13	−77.6 (4)	C3'—P3—C1'—C13'	87.4 (4)
S1—P1—C1—C13	44.0 (4)	S3—P3—C1'—C13'	−34.0 (4)
C2—P1—C1—C11	−70.3 (3)	C2'—P3—C1'—C11'	77.6 (4)
C3—P1—C1—C11	47.3 (3)	C3'—P3—C1'—C11'	−39.4 (4)
S1—P1—C1—C11	168.9 (3)	S3—P3—C1'—C11'	−160.7 (3)
C2—P1—C1—C12	47.1 (4)	C2'—P3—C1'—C12'	−39.8 (4)
C3—P1—C1—C12	164.7 (3)	C3'—P3—C1'—C12'	−156.7 (3)
S1—P1—C1—C12	−73.7 (3)	S3—P3—C1'—C12'	82.0 (3)
C3—P1—C2—C21	170.8 (3)	C3'—P3—C2'—C21'	−165.1 (4)
C1—P1—C2—C21	−69.2 (4)	C1'—P3—C2'—C21'	75.6 (5)
S1—P1—C2—C21	48.5 (3)	S3—P3—C2'—C21'	−43.7 (5)
C3—P1—C2—C22	−56.8 (3)	C3'—P3—C2'—C22'	60.6 (4)
C1—P1—C2—C22	63.2 (3)	C1'—P3—C2'—C22'	−58.7 (4)
S1—P1—C2—C22	−179.1 (3)	S3—P3—C2'—C22'	−178.0 (3)
C2—P1—C3—C31	−167.5 (4)	C2'—P3—C3'—C32'	44.4 (4)
C1—P1—C3—C31	69.9 (4)	C1'—P3—C3'—C32'	166.1 (3)
S1—P1—C3—C31	−46.9 (4)	S3—P3—C3'—C32'	−76.0 (3)
C2—P1—C3—C32	−42.2 (3)	C2'—P3—C3'—C31'	169.0 (4)
C1—P1—C3—C32	−164.7 (3)	C1'—P3—C3'—C31'	−69.3 (4)
S1—P1—C3—C32	78.5 (3)	S3—P3—C3'—C31'	48.6 (4)
C5—P2—C4—C41	−76.6 (3)	C6'—P4—C4'—C42'	−158.7 (3)
C6—P2—C4—C41	40.8 (3)	C5'—P4—C4'—C42'	−41.4 (3)
S2—P2—C4—C41	161.5 (2)	S4—P4—C4'—C42'	80.4 (3)
C5—P2—C4—C42	43.5 (3)	C6'—P4—C4'—C43'	82.1 (3)
C6—P2—C4—C42	160.9 (3)	C5'—P4—C4'—C43'	−160.5 (3)
S2—P2—C4—C42	−78.3 (3)	S4—P4—C4'—C43'	−38.8 (3)
C5—P2—C4—C43	161.2 (3)	C6'—P4—C4'—C41'	−38.4 (3)
C6—P2—C4—C43	−81.4 (3)	C5'—P4—C4'—C41'	78.9 (3)
S2—P2—C4—C43	39.4 (3)	S4—P4—C4'—C41'	−159.3 (2)
C6—P2—C5—C52	−61.1 (3)	C6'—P4—C5'—C51'	−167.3 (3)
C4—P2—C5—C52	58.7 (3)	C4'—P4—C5'—C51'	73.1 (3)
S2—P2—C5—C52	177.8 (2)	S4—P4—C5'—C51'	−46.4 (3)
C6—P2—C5—C51	168.2 (3)	C6'—P4—C5'—C52'	62.5 (3)
C4—P2—C5—C51	−72.0 (3)	C4'—P4—C5'—C52'	−57.1 (3)
S2—P2—C5—C51	47.1 (3)	S4—P4—C5'—C52'	−176.6 (2)
C5—P2—C6—C62	−50.7 (3)	C5'—P4—C6'—C62'	53.1 (3)
C4—P2—C6—C62	−173.3 (3)	C4'—P4—C6'—C62'	175.4 (3)
S2—P2—C6—C62	69.6 (3)	S4—P4—C6'—C62'	−67.0 (3)
C5—P2—C6—C61	−175.7 (3)	C5'—P4—C6'—C61'	176.9 (3)
C4—P2—C6—C61	61.7 (3)	C4'—P4—C6'—C61'	−60.7 (3)
S2—P2—C6—C61	−55.4 (3)	S4—P4—C6'—C61'	56.9 (3)

### (2) Hydrogen-bond geometry (Å, º)

**Table:**

*D*—H···*A*	*D*—H	H···*A*	*D*···*A*	*D*—H···*A*
C32—H32*C*···Au1	0.98	2.73	3.538 (4)	140
C32′—H32*D*···Au2	0.98	2.73	3.503 (5)	136
C13—H13*B*···S1	0.98	2.62	3.220 (5)	120
C43—H43*B*···S2	0.98	2.75	3.219 (4)	110
C43′—H43*D*···S4	0.98	2.73	3.219 (4)	112
C13′—H13*E*···S3	0.98	2.75	3.227 (5)	111
C52′—H52*F*···Cl1	0.98	2.82	3.733 (4)	155
C5′—H5′···Cl2	1.00	2.87	3.839 (4)	163
C3′—H3′···Cl3^i^	1.00	2.88	3.585 (4)	128
C5—H5···Cl4^ii^	1.00	2.79	3.713 (4)	154
C62—H62*B*···Cl4^ii^	0.98	2.83	3.692 (4)	147
C42—H42*C*···Cl5^i^	0.98	2.91	3.754 (4)	145
C11—H11*C*···Cl7^iii^	0.98	2.80	3.736 (5)	161
C6′—H6′···Cl7	1.00	2.91	3.903 (4)	170
C6′—H6′···Au4	1.00	3.28	4.009 (4)	132
C62—H62*A*···Cl8^iv^	0.98	2.93	3.853 (4)	157

Symmetry codes: (i) −*x*+1, −*y*+1, −*z*+1; (ii) *x*−1, *y*, *z*; (iii) −*x*+2, −*y*, −*z*+1; (iv) *x*−1, *y*, *z*−1.

### Bis(tri-tert-butylphosphane sulfide-κS)gold(I) tetrachloridoaurate(III) (3) Crystal data

**Table:**

[Au(C_12_H_27_PS)_2_][AuCl_4_]	*Z* = 1
*M**_r_* = 1004.46	*F*(000) = 486
Triclinic, *P*1	*D*_x_ = 1.946 Mg m^−^^3^
*a* = 8.5541 (2) Å	Mo *K*α radiation, λ = 0.71073 Å
*b* = 9.1550 (3) Å	Cell parameters from 64231 reflections
*c* = 12.0421 (4) Å	θ = 2.4–30.8°
α = 107.427 (3)°	µ = 9.09 mm^−^^1^
β = 97.511 (3)°	*T* = 100 K
γ = 102.841 (3)°	Block, yellow
*V* = 857.30 (5) Å^3^	0.2 × 0.15 × 0.15 mm

### Bis(tri-tert-butylphosphane sulfide-κS)gold(I) tetrachloridoaurate(III) (3) Data collection

**Table:**

Oxford Diffraction Xcalibur, Eos diffractometer	5209 independent reflections
Radiation source: fine-focus sealed X-ray tube	4622 reflections with *I* > 2σ(*I*)
Detector resolution: 16.1419 pixels mm^-1^	*R*_int_ = 0.043
ω–scan	θ_max_ = 31.1°, θ_min_ = 2.4°
Absorption correction: multi-scan (CrysAlisPro; Rigaku OD, 2015)	*h* = −12→12
*T*_min_ = 0.623, *T*_max_ = 1.000	*k* = −13→13
166022 measured reflections	*l* = −17→17

### Bis(tri-tert-butylphosphane sulfide-κS)gold(I) tetrachloridoaurate(III) (3) Refinement

**Table:**

Refinement on *F*^2^	Secondary atom site location: difference Fourier map
Least-squares matrix: full	Hydrogen site location: inferred from neighbouring sites
*R*[*F*^2^ > 2σ(*F*^2^)] = 0.016	H-atom parameters constrained
*wR*(*F*^2^) = 0.038	*w* = 1/[σ^2^(*F*_o_^2^) + (0.0185*P*)^2^ + 0.7041*P*] where *P* = (*F*_o_^2^ + 2*F*_c_^2^)/3
*S* = 1.09	(Δ/σ)_max_ < 0.001
5209 reflections	Δρ_max_ = 1.02 e Å^−^^3^
167 parameters	Δρ_min_ = −1.38 e Å^−^^3^
0 restraints	Extinction correction: *SHELXL2019/3* (Sheldrick, 2015), *F*_c_^*^ = *kF*_c_[1+0.001×*F*_c_^2^λ^3^/sin(2θ)]^-1/4^
Primary atom site location: structure-invariant direct methods	Extinction coefficient: 0.00495 (18)

### Bis(tri-tert-butylphosphane sulfide-κS)gold(I) tetrachloridoaurate(III) (3) Fractional atomic coordinates and isotropic or equivalent isotropic displacement parameters (Å^2^)

**Table:**

	*x*	*y*	*z*	*U*_iso_*/*U*_eq_	
Au1	0.500000	0.000000	0.500000	0.01381 (4)	
P1	0.23333 (6)	0.11351 (5)	0.31699 (4)	0.01003 (9)	
S1	0.32514 (6)	0.15468 (6)	0.49180 (4)	0.01576 (9)	
C1	0.1171 (2)	0.2704 (2)	0.32846 (16)	0.0142 (3)	
C2	0.0891 (2)	−0.0945 (2)	0.24486 (16)	0.0132 (3)	
C3	0.4057 (2)	0.1460 (2)	0.23434 (15)	0.0129 (3)	
C11	−0.0022 (3)	0.2404 (2)	0.21169 (18)	0.0187 (4)	
H11A	−0.048072	0.331055	0.219483	0.028*	
H11B	−0.091156	0.143334	0.194725	0.028*	
H11C	0.056734	0.227528	0.146391	0.028*	
C12	0.2395 (3)	0.4356 (2)	0.36375 (18)	0.0192 (4)	
H12A	0.294950	0.441402	0.298285	0.029*	
H12B	0.321044	0.453291	0.435083	0.029*	
H12C	0.180728	0.517607	0.380378	0.029*	
C13	0.0194 (3)	0.2785 (2)	0.42887 (18)	0.0200 (4)	
H13A	0.095897	0.310702	0.505547	0.030*	
H13B	−0.055894	0.173395	0.413300	0.030*	
H13C	−0.043387	0.356352	0.431261	0.030*	
C21	0.0403 (2)	−0.1412 (2)	0.10879 (16)	0.0168 (4)	
H21A	−0.044660	−0.243312	0.076309	0.025*	
H21B	0.136711	−0.151025	0.074283	0.025*	
H21C	−0.002069	−0.058982	0.089049	0.025*	
C22	−0.0668 (3)	−0.1054 (2)	0.29582 (19)	0.0202 (4)	
H22A	−0.134025	−0.216395	0.266777	0.030*	
H22B	−0.129498	−0.039380	0.270205	0.030*	
H22C	−0.036434	−0.067401	0.382994	0.030*	
C23	0.1666 (3)	−0.2184 (2)	0.27418 (17)	0.0169 (4)	
H23A	0.191786	−0.193494	0.360662	0.025*	
H23B	0.267939	−0.215856	0.244147	0.025*	
H23C	0.089573	−0.324714	0.236401	0.025*	
C31	0.3485 (3)	0.1787 (2)	0.11975 (16)	0.0163 (4)	
H31A	0.439144	0.190646	0.078153	0.024*	
H31B	0.313906	0.276878	0.140200	0.024*	
H31C	0.256104	0.089492	0.067927	0.024*	
C32	0.5508 (2)	0.2864 (2)	0.31569 (18)	0.0174 (4)	
H32A	0.590922	0.263261	0.386809	0.026*	
H32B	0.514561	0.383441	0.339413	0.026*	
H32C	0.639240	0.301838	0.272617	0.026*	
C33	0.4723 (2)	−0.0006 (2)	0.20037 (17)	0.0156 (4)	
H33A	0.567529	0.022387	0.164807	0.023*	
H33B	0.386675	−0.091506	0.142802	0.023*	
H33C	0.505082	−0.026062	0.271857	0.023*	
Au2	0.500000	0.500000	1.000000	0.01173 (3)	
Cl1	0.47385 (6)	0.37675 (5)	0.80089 (4)	0.01857 (9)	
Cl2	0.26132 (6)	0.56585 (6)	0.96144 (5)	0.02260 (10)	

### Bis(tri-tert-butylphosphane sulfide-κS)gold(I) tetrachloridoaurate(III) (3) Atomic displacement parameters (Å^2^)

**Table:**

	*U*^11^	*U*^22^	*U*^33^	*U*^12^	*U*^13^	*U*^23^
Au1	0.01493 (6)	0.01813 (5)	0.01024 (5)	0.00713 (4)	−0.00002 (3)	0.00674 (4)
P1	0.0118 (2)	0.01147 (18)	0.00798 (18)	0.00499 (16)	0.00147 (15)	0.00387 (15)
S1	0.0196 (3)	0.0211 (2)	0.00863 (18)	0.01049 (18)	0.00142 (16)	0.00501 (16)
C1	0.0176 (10)	0.0159 (8)	0.0132 (8)	0.0105 (7)	0.0041 (7)	0.0063 (6)
C2	0.0130 (10)	0.0138 (7)	0.0131 (8)	0.0037 (6)	0.0016 (6)	0.0053 (6)
C3	0.0146 (10)	0.0129 (7)	0.0118 (7)	0.0043 (7)	0.0038 (6)	0.0043 (6)
C11	0.0186 (11)	0.0247 (9)	0.0178 (9)	0.0128 (8)	0.0013 (7)	0.0103 (7)
C12	0.0254 (12)	0.0135 (8)	0.0195 (9)	0.0086 (7)	0.0043 (8)	0.0045 (7)
C13	0.0245 (12)	0.0253 (9)	0.0179 (9)	0.0165 (8)	0.0097 (8)	0.0091 (7)
C21	0.0178 (11)	0.0161 (8)	0.0129 (8)	0.0020 (7)	−0.0006 (7)	0.0033 (6)
C22	0.0156 (11)	0.0232 (9)	0.0237 (10)	0.0048 (8)	0.0068 (8)	0.0104 (8)
C23	0.0201 (11)	0.0130 (8)	0.0186 (9)	0.0042 (7)	0.0029 (7)	0.0078 (7)
C31	0.0198 (11)	0.0169 (8)	0.0138 (8)	0.0048 (7)	0.0048 (7)	0.0074 (7)
C32	0.0133 (10)	0.0163 (8)	0.0192 (9)	0.0012 (7)	0.0010 (7)	0.0043 (7)
C33	0.0145 (10)	0.0170 (8)	0.0170 (8)	0.0071 (7)	0.0049 (7)	0.0057 (7)
Au2	0.01168 (6)	0.00828 (5)	0.01555 (5)	0.00233 (3)	0.00367 (3)	0.00448 (3)
Cl1	0.0220 (3)	0.01572 (19)	0.0169 (2)	0.00531 (17)	0.00461 (17)	0.00374 (15)
Cl2	0.0178 (3)	0.0207 (2)	0.0266 (2)	0.00959 (18)	0.00110 (18)	0.00259 (18)

### Bis(tri-tert-butylphosphane sulfide-κS)gold(I) tetrachloridoaurate(III) (3) Geometric parameters (Å, º)

**Table:**

Au1—S1	2.2889 (5)	C13—H13C	0.9800
Au1—S1^i^	2.2889 (5)	C21—H21A	0.9800
P1—C2	1.8939 (19)	C21—H21B	0.9800
P1—C3	1.9024 (19)	C21—H21C	0.9800
P1—C1	1.9036 (18)	C22—H22A	0.9800
P1—S1	2.0374 (6)	C22—H22B	0.9800
C1—C12	1.536 (3)	C22—H22C	0.9800
C1—C11	1.537 (3)	C23—H23A	0.9800
C1—C13	1.550 (3)	C23—H23B	0.9800
C2—C22	1.535 (3)	C23—H23C	0.9800
C2—C21	1.538 (2)	C31—H31A	0.9800
C2—C23	1.538 (2)	C31—H31B	0.9800
C3—C33	1.538 (2)	C31—H31C	0.9800
C3—C31	1.539 (2)	C32—H32A	0.9800
C3—C32	1.540 (3)	C32—H32B	0.9800
C11—H11A	0.9800	C32—H32C	0.9800
C11—H11B	0.9800	C33—H33A	0.9800
C11—H11C	0.9800	C33—H33B	0.9800
C12—H12A	0.9800	C33—H33C	0.9800
C12—H12B	0.9800	Au2—Cl1^ii^	2.2802 (5)
C12—H12C	0.9800	Au2—Cl1	2.2802 (5)
C13—H13A	0.9800	Au2—Cl2	2.2836 (5)
C13—H13B	0.9800	Au2—Cl2^ii^	2.2836 (5)
			
S1—Au1—S1^i^	180.0	H13B—C13—H13C	109.5
C2—P1—C3	111.14 (8)	C2—C21—H21A	109.5
C2—P1—C1	111.01 (9)	C2—C21—H21B	109.5
C3—P1—C1	111.40 (8)	H21A—C21—H21B	109.5
C2—P1—S1	110.51 (6)	C2—C21—H21C	109.5
C3—P1—S1	110.85 (6)	H21A—C21—H21C	109.5
C1—P1—S1	101.57 (6)	H21B—C21—H21C	109.5
P1—S1—Au1	107.87 (2)	C2—C22—H22A	109.5
C12—C1—C11	108.62 (15)	C2—C22—H22B	109.5
C12—C1—C13	106.15 (15)	H22A—C22—H22B	109.5
C11—C1—C13	108.74 (16)	C2—C22—H22C	109.5
C12—C1—P1	109.58 (13)	H22A—C22—H22C	109.5
C11—C1—P1	112.97 (12)	H22B—C22—H22C	109.5
C13—C1—P1	110.54 (12)	C2—C23—H23A	109.5
C22—C2—C21	108.92 (16)	C2—C23—H23B	109.5
C22—C2—C23	105.94 (15)	H23A—C23—H23B	109.5
C21—C2—C23	108.80 (15)	C2—C23—H23C	109.5
C22—C2—P1	109.76 (13)	H23A—C23—H23C	109.5
C21—C2—P1	112.22 (12)	H23B—C23—H23C	109.5
C23—C2—P1	110.98 (13)	C3—C31—H31A	109.5
C33—C3—C31	108.25 (14)	C3—C31—H31B	109.5
C33—C3—C32	106.34 (16)	H31A—C31—H31B	109.5
C31—C3—C32	109.41 (15)	C3—C31—H31C	109.5
C33—C3—P1	110.79 (12)	H31A—C31—H31C	109.5
C31—C3—P1	111.87 (13)	H31B—C31—H31C	109.5
C32—C3—P1	110.02 (12)	C3—C32—H32A	109.5
C1—C11—H11A	109.5	C3—C32—H32B	109.5
C1—C11—H11B	109.5	H32A—C32—H32B	109.5
H11A—C11—H11B	109.5	C3—C32—H32C	109.5
C1—C11—H11C	109.5	H32A—C32—H32C	109.5
H11A—C11—H11C	109.5	H32B—C32—H32C	109.5
H11B—C11—H11C	109.5	C3—C33—H33A	109.5
C1—C12—H12A	109.5	C3—C33—H33B	109.5
C1—C12—H12B	109.5	H33A—C33—H33B	109.5
H12A—C12—H12B	109.5	C3—C33—H33C	109.5
C1—C12—H12C	109.5	H33A—C33—H33C	109.5
H12A—C12—H12C	109.5	H33B—C33—H33C	109.5
H12B—C12—H12C	109.5	Cl1^ii^—Au2—Cl1	180.0
C1—C13—H13A	109.5	Cl1^ii^—Au2—Cl2	90.336 (18)
C1—C13—H13B	109.5	Cl1—Au2—Cl2	89.664 (18)
H13A—C13—H13B	109.5	Cl1^ii^—Au2—Cl2^ii^	89.664 (18)
C1—C13—H13C	109.5	Cl1—Au2—Cl2^ii^	90.336 (18)
H13A—C13—H13C	109.5	Cl2—Au2—Cl2^ii^	180.0
			
C2—P1—S1—Au1	69.57 (7)	C3—P1—C2—C21	−46.59 (15)
C3—P1—S1—Au1	−54.11 (6)	C1—P1—C2—C21	77.99 (15)
C1—P1—S1—Au1	−172.57 (6)	S1—P1—C2—C21	−170.10 (11)
C2—P1—C1—C12	−168.83 (12)	C3—P1—C2—C23	75.38 (14)
C3—P1—C1—C12	−44.40 (15)	C1—P1—C2—C23	−160.04 (12)
S1—P1—C1—C12	73.67 (13)	S1—P1—C2—C23	−48.13 (14)
C2—P1—C1—C11	−47.57 (16)	C2—P1—C3—C33	−42.58 (15)
C3—P1—C1—C11	76.86 (15)	C1—P1—C3—C33	−166.94 (12)
S1—P1—C1—C11	−165.08 (13)	S1—P1—C3—C33	80.74 (13)
C2—P1—C1—C13	74.52 (15)	C2—P1—C3—C31	78.30 (14)
C3—P1—C1—C13	−161.04 (13)	C1—P1—C3—C31	−46.06 (14)
S1—P1—C1—C13	−42.98 (14)	S1—P1—C3—C31	−158.38 (11)
C3—P1—C2—C22	−167.85 (12)	C2—P1—C3—C32	−159.88 (12)
C1—P1—C2—C22	−43.27 (15)	C1—P1—C3—C32	75.76 (14)
S1—P1—C2—C22	68.64 (13)	S1—P1—C3—C32	−36.56 (13)

Symmetry codes: (i) −*x*+1, −*y*, −*z*+1; (ii) −*x*+1, −*y*+1, −*z*+2.

### Bis(tri-tert-butylphosphane sulfide-κS)gold(I) tetrachloridoaurate(III) (3) Hydrogen-bond geometry (Å, º)

**Table:**

*D*—H···*A*	*D*—H	H···*A*	*D*···*A*	*D*—H···*A*
C23—H23*A*···Au1	0.98	2.81	3.421 (2)	121
C33—H33*C*···Au1	0.98	2.69	3.5832 (19)	151
C13—H13*A*···S1	0.98	2.66	3.164 (2)	112
C32—H32*A*···S1	0.98	2.87	3.353 (2)	111
C12—H12*A*···Cl1^iii^	0.98	2.91	3.786 (2)	150
C22—H22*A*···Cl1^iv^	0.98	2.83	3.607 (2)	136
C23—H23*B*···Cl1^i^	0.98	2.94	3.782 (2)	145

Symmetry codes: (i) −*x*+1, −*y*, −*z*+1; (iii) −*x*+1, −*y*+1, −*z*+1; (iv) −*x*, −*y*, −*z*+1.

### (4a) Crystal data

**Table:**

[C_20_H_46_AuP_2_S_2_][AuBr_4_]	*F*(000) = 2104
*M**_r_* = 1126.20	*D*_x_ = 2.298 Mg m^−^^3^
Monoclinic, *P*2_1_/*c*	Mo *K*α radiation, λ = 0.71073 Å
*a* = 13.7871 (4) Å	Cell parameters from 21928 reflections
*b* = 10.4042 (3) Å	θ = 2.2–30.8°
*c* = 22.7240 (6) Å	µ = 14.15 mm^−^^1^
β = 93.035 (3)°	*T* = 100 K
*V* = 3255.05 (16) Å^3^	Plate, red
*Z* = 4	0.25 × 0.15 × 0.02 mm

### (4a) Data collection

**Table:**

Oxford Diffraction Xcalibur, Eos diffractometer	6651 independent reflections
Radiation source: fine-focus sealed tube	5373 reflections with *I* > 2σ(*I*)
Graphite monochromator	*R*_int_ = 0.086
Detector resolution: 16.1419 pixels mm^-1^	θ_max_ = 26.4°, θ_min_ = 2.2°
ω scans	*h* = −17→17
Absorption correction: multi-scan (CrysAlisPro; Rigaku OD, 2015)	*k* = −13→13
*T*_min_ = 0.179, *T*_max_ = 1.000	*l* = −28→28
169756 measured reflections	

### (4a) Refinement

**Table:**

Refinement on *F*^2^	Primary atom site location: structure-invariant direct methods
Least-squares matrix: full	Secondary atom site location: difference Fourier map
*R*[*F*^2^ > 2σ(*F*^2^)] = 0.046	Hydrogen site location: inferred from neighbouring sites
*wR*(*F*^2^) = 0.112	H-atom parameters constrained
*S* = 1.01	*w* = 1/[σ^2^(*F*_o_^2^) + (0.0456*P*)^2^ + 52.5404*P*] where *P* = (*F*_o_^2^ + 2*F*_c_^2^)/3
6651 reflections	(Δ/σ)_max_ = 0.001
288 parameters	Δρ_max_ = 4.29 e Å^−^^3^
0 restraints	Δρ_min_ = −2.01 e Å^−^^3^

### (4a) Fractional atomic coordinates and isotropic or equivalent isotropic displacement parameters (Å^2^)

**Table:**

	*x*	*y*	*z*	*U*_iso_*/*U*_eq_	
Au1	0.24485 (2)	0.11240 (3)	0.25008 (2)	0.02621 (10)	
S1	0.41029 (16)	0.0981 (2)	0.24669 (10)	0.0324 (5)	
S2	0.07841 (15)	0.1215 (2)	0.25162 (9)	0.0244 (4)	
P1	0.42826 (15)	0.0729 (2)	0.15946 (10)	0.0263 (5)	
P2	0.05250 (14)	0.1109 (2)	0.33849 (9)	0.0185 (4)	
C1	0.5617 (7)	0.0747 (12)	0.1503 (5)	0.042 (3)	
C2	0.3598 (6)	0.1931 (9)	0.1152 (4)	0.035 (2)	
H2	0.290131	0.171352	0.120356	0.042*	
C3	0.3793 (7)	−0.0843 (9)	0.1331 (4)	0.034 (2)	
H3	0.403408	−0.098618	0.092919	0.041*	
C4	−0.0785 (6)	0.0723 (8)	0.3419 (4)	0.0253 (18)	
C5	0.1320 (6)	−0.0082 (8)	0.3757 (4)	0.0265 (19)	
H5	0.198759	0.029053	0.374540	0.032*	
C6	0.0793 (6)	0.2636 (8)	0.3766 (4)	0.0258 (18)	
H6	0.057290	0.253613	0.417557	0.031*	
C11	0.5854 (8)	0.0056 (14)	0.0919 (5)	0.058 (3)	
H11A	0.549249	0.046773	0.058669	0.086*	
H11B	0.566570	−0.085013	0.093993	0.086*	
H11C	0.655200	0.011751	0.086195	0.086*	
C12	0.5926 (8)	0.2149 (14)	0.1483 (6)	0.066 (4)	
H12A	0.573351	0.258815	0.184025	0.098*	
H12B	0.561060	0.256358	0.113609	0.098*	
H12C	0.663262	0.219893	0.145991	0.098*	
C13	0.6164 (7)	0.0072 (13)	0.2019 (5)	0.053 (3)	
H13A	0.685956	0.005015	0.194901	0.080*	
H13B	0.592112	−0.080852	0.205350	0.080*	
H13C	0.606326	0.054134	0.238455	0.080*	
C21	0.3703 (8)	0.3304 (10)	0.1368 (5)	0.051 (3)	
H21A	0.434797	0.362892	0.128362	0.076*	
H21B	0.362406	0.333117	0.179423	0.076*	
H21C	0.320426	0.384062	0.116684	0.076*	
C22	0.3721 (7)	0.1803 (12)	0.0489 (5)	0.047 (3)	
H22A	0.327595	0.239615	0.027542	0.070*	
H22B	0.357354	0.091942	0.036443	0.070*	
H22C	0.439150	0.201201	0.040191	0.070*	
C31	0.4160 (9)	−0.1961 (10)	0.1715 (6)	0.056 (3)	
H31A	0.403905	−0.178072	0.212789	0.084*	
H31B	0.485941	−0.207142	0.167340	0.084*	
H31C	0.381921	−0.274922	0.159017	0.084*	
C32	0.2687 (7)	−0.0875 (10)	0.1270 (5)	0.044 (3)	
H32A	0.247338	−0.169714	0.109731	0.067*	
H32B	0.245345	−0.016976	0.101434	0.067*	
H32C	0.242271	−0.077874	0.165978	0.067*	
C41	−0.1141 (6)	0.1065 (10)	0.4030 (4)	0.035 (2)	
H41A	−0.182794	0.083275	0.404724	0.052*	
H41B	−0.106347	0.198989	0.409927	0.052*	
H41C	−0.075846	0.058925	0.433394	0.052*	
C42	−0.0943 (7)	−0.0724 (9)	0.3307 (5)	0.037 (2)	
H42A	−0.068124	−0.121210	0.364893	0.056*	
H42B	−0.060853	−0.098273	0.295628	0.056*	
H42C	−0.163946	−0.089882	0.324619	0.056*	
C43	−0.1389 (6)	0.1458 (10)	0.2934 (4)	0.035 (2)	
H43A	−0.208206	0.131701	0.298556	0.053*	
H43B	−0.122229	0.114395	0.254591	0.053*	
H43C	−0.124468	0.237874	0.296491	0.053*	
C51	0.1386 (8)	−0.1385 (9)	0.3453 (5)	0.040 (2)	
H51A	0.193024	−0.187475	0.363524	0.060*	
H51B	0.149021	−0.125584	0.303408	0.060*	
H51C	0.078043	−0.186117	0.349524	0.060*	
C52	0.1148 (7)	−0.0230 (10)	0.4419 (4)	0.035 (2)	
H52A	0.052543	−0.066256	0.446579	0.053*	
H52B	0.113586	0.062093	0.460311	0.053*	
H52C	0.167320	−0.074274	0.460772	0.053*	
C61	0.0213 (8)	0.3770 (9)	0.3487 (4)	0.039 (2)	
H61A	0.047881	0.458103	0.364470	0.058*	
H61B	−0.047003	0.369536	0.358168	0.058*	
H61C	0.026189	0.375374	0.305875	0.058*	
C62	0.1868 (7)	0.2922 (11)	0.3823 (5)	0.049 (3)	
H62A	0.211143	0.306366	0.343095	0.074*	
H62B	0.221040	0.219364	0.401179	0.074*	
H62C	0.197859	0.369531	0.406374	0.074*	
Au2	0.500000	0.000000	0.500000	0.03200 (14)	
Br1	0.62987 (7)	0.15379 (12)	0.48418 (5)	0.0454 (3)	
Br2	0.41237 (7)	0.07330 (12)	0.41121 (5)	0.0455 (3)	
Au3	1.000000	0.500000	0.500000	0.02361 (12)	
Br3	0.90685 (7)	0.67401 (9)	0.45406 (4)	0.0342 (2)	
Br4	0.85112 (6)	0.38951 (8)	0.52231 (4)	0.0311 (2)	

### (4a) Atomic displacement parameters (Å^2^)

**Table:**

	*U*^11^	*U*^22^	*U*^33^	*U*^12^	*U*^13^	*U*^23^
Au1	0.02586 (17)	0.03220 (19)	0.02182 (17)	−0.00487 (14)	0.01293 (13)	−0.00523 (14)
S1	0.0265 (11)	0.0505 (14)	0.0207 (11)	−0.0065 (10)	0.0061 (8)	−0.0088 (10)
S2	0.0254 (10)	0.0293 (11)	0.0193 (10)	−0.0019 (8)	0.0091 (8)	−0.0014 (8)
P1	0.0190 (10)	0.0409 (13)	0.0195 (11)	0.0044 (9)	0.0062 (8)	−0.0019 (10)
P2	0.0187 (9)	0.0209 (10)	0.0166 (10)	0.0025 (8)	0.0063 (7)	−0.0001 (8)
C1	0.022 (4)	0.068 (7)	0.038 (6)	0.005 (5)	0.009 (4)	0.006 (5)
C2	0.021 (4)	0.041 (6)	0.044 (6)	0.003 (4)	0.011 (4)	0.007 (4)
C3	0.037 (5)	0.040 (5)	0.026 (5)	0.002 (4)	0.005 (4)	−0.010 (4)
C4	0.026 (4)	0.029 (4)	0.022 (4)	−0.002 (3)	0.006 (3)	−0.002 (3)
C5	0.030 (4)	0.027 (4)	0.023 (5)	0.009 (4)	0.010 (4)	0.001 (3)
C6	0.030 (4)	0.029 (5)	0.019 (4)	0.003 (4)	0.003 (3)	−0.002 (3)
C11	0.032 (6)	0.096 (10)	0.046 (7)	0.015 (6)	0.016 (5)	0.003 (6)
C12	0.034 (6)	0.087 (10)	0.077 (9)	−0.024 (6)	0.012 (6)	0.011 (8)
C13	0.025 (5)	0.092 (10)	0.043 (7)	0.008 (5)	0.000 (4)	0.006 (6)
C21	0.056 (7)	0.035 (6)	0.063 (8)	−0.001 (5)	0.024 (6)	0.006 (5)
C22	0.035 (5)	0.069 (8)	0.037 (6)	−0.001 (5)	0.003 (4)	0.016 (5)
C31	0.071 (8)	0.032 (6)	0.064 (8)	0.007 (5)	−0.009 (6)	0.003 (5)
C32	0.046 (6)	0.037 (6)	0.051 (7)	−0.011 (5)	0.004 (5)	−0.006 (5)
C41	0.021 (4)	0.051 (6)	0.033 (5)	0.005 (4)	0.011 (4)	0.006 (4)
C42	0.037 (5)	0.032 (5)	0.044 (6)	−0.004 (4)	0.018 (4)	0.000 (4)
C43	0.026 (4)	0.049 (6)	0.031 (5)	0.008 (4)	0.003 (4)	0.002 (4)
C51	0.051 (6)	0.035 (5)	0.036 (6)	0.018 (5)	0.012 (5)	0.002 (4)
C52	0.036 (5)	0.041 (5)	0.029 (5)	0.010 (4)	0.006 (4)	0.010 (4)
C61	0.059 (6)	0.029 (5)	0.029 (5)	0.007 (4)	0.010 (4)	0.000 (4)
C62	0.041 (6)	0.046 (6)	0.062 (7)	−0.010 (5)	0.012 (5)	−0.025 (6)
Au2	0.0213 (2)	0.0516 (3)	0.0233 (3)	0.0109 (2)	0.00309 (18)	−0.0048 (2)
Br1	0.0338 (5)	0.0646 (7)	0.0379 (6)	−0.0013 (5)	0.0037 (4)	0.0039 (5)
Br2	0.0384 (5)	0.0619 (7)	0.0356 (6)	0.0105 (5)	−0.0036 (4)	0.0010 (5)
Au3	0.0247 (2)	0.0193 (2)	0.0267 (3)	−0.00104 (17)	0.00136 (18)	0.00288 (17)
Br3	0.0332 (5)	0.0257 (4)	0.0435 (6)	0.0025 (4)	−0.0003 (4)	0.0066 (4)
Br4	0.0286 (4)	0.0278 (4)	0.0370 (5)	−0.0054 (3)	0.0040 (4)	0.0032 (4)

### (4a) Geometric parameters (Å, º)

**Table:**

Au1—S1	2.291 (2)	C21—H21C	0.9800
Au1—S2	2.299 (2)	C22—H22A	0.9800
S1—P1	2.028 (3)	C22—H22B	0.9800
S2—P2	2.028 (3)	C22—H22C	0.9800
P1—C2	1.834 (10)	C31—H31A	0.9800
P1—C3	1.857 (10)	C31—H31B	0.9800
P1—C1	1.862 (9)	C31—H31C	0.9800
P2—C5	1.831 (9)	C32—H32A	0.9800
P2—C6	1.838 (9)	C32—H32B	0.9800
P2—C4	1.856 (8)	C32—H32C	0.9800
C1—C12	1.521 (17)	C41—H41A	0.9800
C1—C13	1.531 (14)	C41—H41B	0.9800
C1—C11	1.561 (15)	C41—H41C	0.9800
C2—C21	1.515 (15)	C42—H42A	0.9800
C2—C22	1.532 (14)	C42—H42B	0.9800
C2—H2	1.0000	C42—H42C	0.9800
C3—C31	1.524 (14)	C43—H43A	0.9800
C3—C32	1.524 (13)	C43—H43B	0.9800
C3—H3	1.0000	C43—H43C	0.9800
C4—C41	1.540 (12)	C51—H51A	0.9800
C4—C42	1.541 (13)	C51—H51B	0.9800
C4—C43	1.546 (12)	C51—H51C	0.9800
C5—C51	1.527 (12)	C52—H52A	0.9800
C5—C52	1.542 (12)	C52—H52B	0.9800
C5—H5	1.0000	C52—H52C	0.9800
C6—C62	1.510 (13)	C61—H61A	0.9800
C6—C61	1.542 (12)	C61—H61B	0.9800
C6—H6	1.0000	C61—H61C	0.9800
C11—H11A	0.9800	C62—H62A	0.9800
C11—H11B	0.9800	C62—H62B	0.9800
C11—H11C	0.9800	C62—H62C	0.9800
C12—H12A	0.9800	Au2—Br2^i^	2.4195 (10)
C12—H12B	0.9800	Au2—Br2	2.4196 (10)
C12—H12C	0.9800	Au2—Br1	2.4421 (11)
C13—H13A	0.9800	Au2—Br1^i^	2.4421 (11)
C13—H13B	0.9800	Au3—Br3^ii^	2.4238 (9)
C13—H13C	0.9800	Au3—Br3	2.4238 (9)
C21—H21A	0.9800	Au3—Br4	2.4294 (8)
C21—H21B	0.9800	Au3—Br4^ii^	2.4294 (8)
			
S1—Au1—S2	178.28 (8)	H21B—C21—H21C	109.5
P1—S1—Au1	102.39 (11)	C2—C22—H22A	109.5
P2—S2—Au1	103.89 (11)	C2—C22—H22B	109.5
C2—P1—C3	104.9 (5)	H22A—C22—H22B	109.5
C2—P1—C1	114.5 (5)	C2—C22—H22C	109.5
C3—P1—C1	108.5 (5)	H22A—C22—H22C	109.5
C2—P1—S1	111.2 (3)	H22B—C22—H22C	109.5
C3—P1—S1	111.6 (3)	C3—C31—H31A	109.5
C1—P1—S1	106.3 (3)	C3—C31—H31B	109.5
C5—P2—C6	105.5 (4)	H31A—C31—H31B	109.5
C5—P2—C4	113.3 (4)	C3—C31—H31C	109.5
C6—P2—C4	109.8 (4)	H31A—C31—H31C	109.5
C5—P2—S2	110.8 (3)	H31B—C31—H31C	109.5
C6—P2—S2	111.7 (3)	C3—C32—H32A	109.5
C4—P2—S2	105.9 (3)	C3—C32—H32B	109.5
C12—C1—C13	109.6 (10)	H32A—C32—H32B	109.5
C12—C1—C11	110.1 (10)	C3—C32—H32C	109.5
C13—C1—C11	108.9 (9)	H32A—C32—H32C	109.5
C12—C1—P1	107.0 (7)	H32B—C32—H32C	109.5
C13—C1—P1	111.1 (7)	C4—C41—H41A	109.5
C11—C1—P1	110.0 (7)	C4—C41—H41B	109.5
C21—C2—C22	112.8 (9)	H41A—C41—H41B	109.5
C21—C2—P1	115.3 (8)	C4—C41—H41C	109.5
C22—C2—P1	113.6 (7)	H41A—C41—H41C	109.5
C21—C2—H2	104.6	H41B—C41—H41C	109.5
C22—C2—H2	104.6	C4—C42—H42A	109.5
P1—C2—H2	104.6	C4—C42—H42B	109.5
C31—C3—C32	109.6 (9)	H42A—C42—H42B	109.5
C31—C3—P1	112.6 (7)	C4—C42—H42C	109.5
C32—C3—P1	113.2 (7)	H42A—C42—H42C	109.5
C31—C3—H3	107.0	H42B—C42—H42C	109.5
C32—C3—H3	107.0	C4—C43—H43A	109.5
P1—C3—H3	107.0	C4—C43—H43B	109.5
C41—C4—C42	108.9 (7)	H43A—C43—H43B	109.5
C41—C4—C43	110.1 (7)	C4—C43—H43C	109.5
C42—C4—C43	107.5 (8)	H43A—C43—H43C	109.5
C41—C4—P2	110.2 (6)	H43B—C43—H43C	109.5
C42—C4—P2	109.5 (6)	C5—C51—H51A	109.5
C43—C4—P2	110.6 (6)	C5—C51—H51B	109.5
C51—C5—C52	111.6 (8)	H51A—C51—H51B	109.5
C51—C5—P2	116.1 (7)	C5—C51—H51C	109.5
C52—C5—P2	113.5 (6)	H51A—C51—H51C	109.5
C51—C5—H5	104.8	H51B—C51—H51C	109.5
C52—C5—H5	104.8	C5—C52—H52A	109.5
P2—C5—H5	104.8	C5—C52—H52B	109.5
C62—C6—C61	111.7 (8)	H52A—C52—H52B	109.5
C62—C6—P2	112.5 (6)	C5—C52—H52C	109.5
C61—C6—P2	112.4 (6)	H52A—C52—H52C	109.5
C62—C6—H6	106.6	H52B—C52—H52C	109.5
C61—C6—H6	106.6	C6—C61—H61A	109.5
P2—C6—H6	106.6	C6—C61—H61B	109.5
C1—C11—H11A	109.5	H61A—C61—H61B	109.5
C1—C11—H11B	109.5	C6—C61—H61C	109.5
H11A—C11—H11B	109.5	H61A—C61—H61C	109.5
C1—C11—H11C	109.5	H61B—C61—H61C	109.5
H11A—C11—H11C	109.5	C6—C62—H62A	109.5
H11B—C11—H11C	109.5	C6—C62—H62B	109.5
C1—C12—H12A	109.5	H62A—C62—H62B	109.5
C1—C12—H12B	109.5	C6—C62—H62C	109.5
H12A—C12—H12B	109.5	H62A—C62—H62C	109.5
C1—C12—H12C	109.5	H62B—C62—H62C	109.5
H12A—C12—H12C	109.5	Br2^i^—Au2—Br2	180.0
H12B—C12—H12C	109.5	Br2^i^—Au2—Br1	89.52 (4)
C1—C13—H13A	109.5	Br2—Au2—Br1	90.48 (4)
C1—C13—H13B	109.5	Br2^i^—Au2—Br1^i^	90.48 (4)
H13A—C13—H13B	109.5	Br2—Au2—Br1^i^	89.52 (4)
C1—C13—H13C	109.5	Br1—Au2—Br1^i^	180.0
H13A—C13—H13C	109.5	Br3^ii^—Au3—Br3	180.0
H13B—C13—H13C	109.5	Br3^ii^—Au3—Br4	89.48 (3)
C2—C21—H21A	109.5	Br3—Au3—Br4	90.52 (3)
C2—C21—H21B	109.5	Br3^ii^—Au3—Br4^ii^	90.52 (3)
H21A—C21—H21B	109.5	Br3—Au3—Br4^ii^	89.48 (3)
C2—C21—H21C	109.5	Br4—Au3—Br4^ii^	180.0
H21A—C21—H21C	109.5		
			
Au1—S1—P1—C2	49.8 (3)	C2—P1—C3—C32	−45.8 (8)
Au1—S1—P1—C3	−66.9 (3)	C1—P1—C3—C32	−168.6 (7)
Au1—S1—P1—C1	175.0 (4)	S1—P1—C3—C32	74.7 (8)
Au1—S2—P2—C5	−41.5 (3)	C5—P2—C4—C41	77.6 (7)
Au1—S2—P2—C6	75.8 (3)	C6—P2—C4—C41	−40.0 (7)
Au1—S2—P2—C4	−164.7 (3)	S2—P2—C4—C41	−160.8 (6)
C2—P1—C1—C12	41.1 (9)	C5—P2—C4—C42	−42.2 (7)
C3—P1—C1—C12	157.9 (8)	C6—P2—C4—C42	−159.9 (6)
S1—P1—C1—C12	−82.0 (8)	S2—P2—C4—C42	79.4 (6)
C2—P1—C1—C13	160.8 (8)	C5—P2—C4—C43	−160.5 (6)
C3—P1—C1—C13	−82.4 (9)	C6—P2—C4—C43	81.9 (7)
S1—P1—C1—C13	37.6 (9)	S2—P2—C4—C43	−38.9 (6)
C2—P1—C1—C11	−78.5 (9)	C6—P2—C5—C51	−168.6 (7)
C3—P1—C1—C11	38.2 (9)	C4—P2—C5—C51	71.2 (8)
S1—P1—C1—C11	158.3 (7)	S2—P2—C5—C51	−47.6 (7)
C3—P1—C2—C21	167.3 (7)	C6—P2—C5—C52	60.0 (8)
C1—P1—C2—C21	−73.9 (8)	C4—P2—C5—C52	−60.1 (8)
S1—P1—C2—C21	46.6 (8)	S2—P2—C5—C52	−178.9 (6)
C3—P1—C2—C22	−60.3 (8)	C5—P2—C6—C62	50.3 (8)
C1—P1—C2—C22	58.5 (9)	C4—P2—C6—C62	172.6 (7)
S1—P1—C2—C22	179.0 (6)	S2—P2—C6—C62	−70.2 (8)
C2—P1—C3—C31	−170.9 (8)	C5—P2—C6—C61	177.4 (6)
C1—P1—C3—C31	66.4 (9)	C4—P2—C6—C61	−60.2 (7)
S1—P1—C3—C31	−50.4 (8)	S2—P2—C6—C61	57.0 (7)

Symmetry codes: (i) −*x*+1, −*y*, −*z*+1; (ii) −*x*+2, −*y*+1, −*z*+1.

### (4a) Hydrogen-bond geometry (Å, º)

**Table:**

*D*—H···*A*	*D*—H	H···*A*	*D*···*A*	*D*—H···*A*
C32—H32*C*···Au1	0.98	2.75	3.514 (11)	135
C13—H13*C*···S1	0.98	2.76	3.212 (11)	109
C43—H43*B*···S2	0.98	2.77	3.201 (9)	107
C3—H3···Br1^iii^	1.00	3.14	3.809 (9)	126
C52—H52*C*···Br1^i^	0.98	3.11	4.055 (9)	161
C5—H5···Br2	1.00	3.05	3.997 (9)	158
C62—H62*B*···Br2	0.98	3.04	3.883 (12)	145
C2—H2···Br3^iii^	1.00	3.12	3.927 (9)	139
C6—H6···Br3^iv^	1.00	3.03	3.898 (8)	146
C32—H32*B*···Br3^iii^	0.98	3.11	4.023 (11)	156
C42—H42*A*···Br3^v^	0.98	2.97	3.848 (10)	149
C62—H62*C*···Br4^iv^	0.98	3.08	4.007 (10)	158

Symmetry codes: (i) −*x*+1, −*y*, −*z*+1; (iii) −*x*+1, *y*−1/2, −*z*+1/2; (iv) −*x*+1, −*y*+1, −*z*+1; (v) *x*−1, *y*−1, *z*.

### Bis[tert-butylbis(propan-2-yl)-λ5-phosphaneselanone-κSe]gold(I) tetrabromidoaurate(III) (4b) Crystal data

**Table:**

[Au(C_10_H_23_PSe)_2_][AuBr_4_]	*F*(000) = 2248
*M**_r_* = 1220.00	*D*_x_ = 2.460 Mg m^−^^3^
Monoclinic, *P*2_1_/*c*	Mo *K*α radiation, λ = 0.71073 Å
*a* = 13.7265 (3) Å	Cell parameters from 14834 reflections
*b* = 10.5615 (3) Å	θ = 2.1–30.9°
*c* = 22.7782 (5) Å	µ = 16.07 mm^−^^1^
β = 94.096 (2)°	*T* = 100 K
*V* = 3293.78 (14) Å^3^	Block, red
*Z* = 4	0.15 × 0.10 × 0.05 mm

### Bis[tert-butylbis(propan-2-yl)-λ5-phosphaneselanone-κSe]gold(I) tetrabromidoaurate(III) (4b) Data collection

**Table:**

Oxford Diffraction Xcalibur, Eos diffractometer	9556 independent reflections
Radiation source: fine-focus sealed tube	7328 reflections with *I* > 2σ(*I*)
Graphite monochromator	*R*_int_ = 0.068
Detector resolution: 16.1419 pixels mm^-1^	θ_max_ = 30.0°, θ_min_ = 2.1°
ω–scan	*h* = −19→18
Absorption correction: multi-scan (CrysAlisPro; Rigaku OD, 2015)	*k* = −14→14
*T*_min_ = 0.589, *T*_max_ = 1.000	*l* = −31→32
106256 measured reflections	

### Bis[tert-butylbis(propan-2-yl)-λ5-phosphaneselanone-κSe]gold(I) tetrabromidoaurate(III) (4b) Refinement

**Table:**

Refinement on *F*^2^	Primary atom site location: structure-invariant direct methods
Least-squares matrix: full	Secondary atom site location: difference Fourier map
*R*[*F*^2^ > 2σ(*F*^2^)] = 0.030	Hydrogen site location: inferred from neighbouring sites
*wR*(*F*^2^) = 0.049	H-atom parameters constrained
*S* = 1.03	*w* = 1/[σ^2^(*F*_o_^2^) + (0.012*P*)^2^ + 5.9573*P*] where *P* = (*F*_o_^2^ + 2*F*_c_^2^)/3
9556 reflections	(Δ/σ)_max_ = 0.001
288 parameters	Δρ_max_ = 1.57 e Å^−^^3^
0 restraints	Δρ_min_ = −1.09 e Å^−^^3^

### Bis[tert-butylbis(propan-2-yl)-λ5-phosphaneselanone-κSe]gold(I) tetrabromidoaurate(III) (4b) Fractional atomic coordinates and isotropic or equivalent isotropic displacement parameters (Å^2^)

**Table:**

	*x*	*y*	*z*	*U*_iso_*/*U*_eq_	
Au1	0.24873 (2)	0.10808 (2)	0.25292 (2)	0.01848 (4)	
Se1	0.42270 (3)	0.08281 (4)	0.25371 (2)	0.02074 (9)	
Se2	0.07358 (3)	0.12570 (4)	0.24726 (2)	0.01833 (9)	
P1	0.43426 (7)	0.06549 (10)	0.15853 (4)	0.0141 (2)	
P2	0.04571 (7)	0.10969 (9)	0.34028 (4)	0.0134 (2)	
C1	0.5678 (3)	0.0643 (4)	0.14645 (19)	0.0236 (10)	
C2	0.3652 (3)	0.1931 (4)	0.11898 (18)	0.0194 (9)	
H2	0.295074	0.175806	0.125240	0.023*	
C3	0.3795 (3)	−0.0832 (4)	0.12957 (17)	0.0153 (8)	
H3	0.397880	−0.092943	0.088119	0.018*	
C4	−0.0870 (3)	0.0738 (4)	0.34291 (17)	0.0172 (8)	
C5	0.1267 (3)	−0.0098 (4)	0.37674 (17)	0.0165 (8)	
H5	0.194062	0.025662	0.375221	0.020*	
C6	0.0740 (3)	0.2594 (4)	0.37980 (17)	0.0171 (8)	
H6	0.050848	0.250060	0.420222	0.021*	
C11	0.5845 (3)	0.0012 (5)	0.08645 (19)	0.0308 (11)	
H11A	0.544312	0.043902	0.055113	0.046*	
H11B	0.566130	−0.088336	0.087749	0.046*	
H11C	0.653543	0.008335	0.078613	0.046*	
C12	0.6047 (3)	0.2010 (5)	0.1463 (2)	0.0331 (12)	
H12A	0.587663	0.243740	0.182332	0.050*	
H12B	0.574290	0.245624	0.111966	0.050*	
H12C	0.675814	0.201222	0.144413	0.050*	
C13	0.6256 (3)	−0.0082 (5)	0.19610 (19)	0.0314 (11)	
H13A	0.694832	−0.010263	0.188273	0.047*	
H13B	0.600627	−0.094936	0.197944	0.047*	
H13C	0.618066	0.034451	0.233725	0.047*	
C21	0.3847 (3)	0.3265 (4)	0.1430 (2)	0.0311 (11)	
H21A	0.448024	0.356236	0.131240	0.047*	
H21B	0.385387	0.325042	0.186071	0.047*	
H21C	0.333086	0.383713	0.127183	0.047*	
C22	0.3722 (3)	0.1874 (4)	0.05189 (18)	0.0251 (10)	
H22A	0.326132	0.248084	0.032713	0.038*	
H22B	0.356010	0.101816	0.037759	0.038*	
H22C	0.438734	0.208837	0.042465	0.038*	
C31	0.4188 (3)	−0.1992 (4)	0.16431 (19)	0.0274 (10)	
H31A	0.409001	−0.187487	0.206161	0.041*	
H31B	0.488618	−0.209048	0.159131	0.041*	
H31C	0.383729	−0.275104	0.149802	0.041*	
C32	0.2679 (3)	−0.0823 (4)	0.12822 (19)	0.0240 (10)	
H32A	0.242184	−0.160863	0.110274	0.036*	
H32B	0.242496	−0.009761	0.105043	0.036*	
H32C	0.247573	−0.075548	0.168477	0.036*	
C41	−0.1208 (3)	0.1055 (4)	0.40416 (18)	0.0231 (9)	
H41A	−0.189397	0.081130	0.405943	0.035*	
H41B	−0.113890	0.196704	0.411331	0.035*	
H41C	−0.080627	0.059008	0.434214	0.035*	
C42	−0.1041 (3)	−0.0681 (4)	0.3305 (2)	0.0267 (10)	
H42A	−0.073381	−0.118090	0.362976	0.040*	
H42B	−0.075486	−0.091157	0.293759	0.040*	
H42C	−0.174460	−0.085360	0.326776	0.040*	
C43	−0.1477 (3)	0.1481 (4)	0.29472 (18)	0.0215 (9)	
H43A	−0.217398	0.135308	0.299443	0.032*	
H43B	−0.131990	0.117656	0.255859	0.032*	
H43C	−0.132170	0.238473	0.298253	0.032*	
C51	0.1300 (3)	−0.1379 (4)	0.3458 (2)	0.0262 (10)	
H51A	0.188011	−0.184748	0.361037	0.039*	
H51B	0.132754	−0.124701	0.303362	0.039*	
H51C	0.071231	−0.186470	0.353138	0.039*	
C52	0.1124 (3)	−0.0258 (4)	0.44255 (17)	0.0233 (9)	
H52A	0.051663	−0.072157	0.447351	0.035*	
H52B	0.108884	0.057749	0.461002	0.035*	
H52C	0.167590	−0.073191	0.461309	0.035*	
C61	0.0203 (3)	0.3717 (4)	0.35124 (19)	0.0237 (10)	
H61A	0.043459	0.449991	0.370687	0.035*	
H61B	−0.049992	0.362428	0.355154	0.035*	
H61C	0.032726	0.375305	0.309436	0.035*	
C62	0.1842 (3)	0.2847 (4)	0.3870 (2)	0.0271 (10)	
H62A	0.210806	0.285379	0.348149	0.041*	
H62B	0.216222	0.217969	0.411220	0.041*	
H62C	0.196118	0.366949	0.406064	0.041*	
Au2	0.500000	0.000000	0.500000	0.01658 (5)	
Br1	0.62575 (3)	0.15694 (4)	0.48524 (2)	0.02692 (10)	
Br2	0.40674 (3)	0.07184 (4)	0.41181 (2)	0.02478 (10)	
Au3	1.000000	0.500000	0.500000	0.01530 (5)	
Br3	0.90816 (3)	0.67316 (4)	0.45277 (2)	0.02375 (9)	
Br4	0.84937 (3)	0.39442 (4)	0.52190 (2)	0.02172 (9)	

### Bis[tert-butylbis(propan-2-yl)-λ5-phosphaneselanone-κSe]gold(I) tetrabromidoaurate(III) (4b) Atomic displacement parameters (Å^2^)

**Table:**

	*U*^11^	*U*^22^	*U*^33^	*U*^12^	*U*^13^	*U*^23^
Au1	0.02021 (8)	0.02011 (8)	0.01628 (8)	−0.00264 (7)	0.00940 (6)	−0.00332 (7)
Se1	0.0194 (2)	0.0312 (2)	0.0120 (2)	−0.00504 (18)	0.00327 (15)	−0.00602 (17)
Se2	0.0206 (2)	0.0221 (2)	0.0129 (2)	0.00093 (16)	0.00597 (15)	−0.00077 (16)
P1	0.0118 (5)	0.0191 (5)	0.0115 (5)	−0.0008 (4)	0.0023 (4)	−0.0010 (4)
P2	0.0134 (5)	0.0143 (5)	0.0126 (5)	0.0022 (4)	0.0032 (4)	0.0002 (4)
C1	0.0097 (19)	0.037 (3)	0.024 (2)	0.0010 (18)	0.0065 (16)	0.001 (2)
C2	0.016 (2)	0.018 (2)	0.025 (2)	−0.0017 (16)	0.0048 (17)	0.0036 (17)
C3	0.018 (2)	0.0153 (19)	0.013 (2)	−0.0002 (15)	0.0000 (15)	−0.0025 (16)
C4	0.0156 (19)	0.019 (2)	0.018 (2)	0.0035 (16)	0.0041 (16)	0.0003 (17)
C5	0.0136 (19)	0.017 (2)	0.019 (2)	0.0050 (16)	0.0030 (15)	0.0027 (16)
C6	0.019 (2)	0.019 (2)	0.014 (2)	0.0014 (16)	0.0010 (15)	−0.0017 (16)
C11	0.023 (2)	0.046 (3)	0.025 (3)	0.005 (2)	0.0104 (19)	0.000 (2)
C12	0.018 (2)	0.046 (3)	0.035 (3)	−0.014 (2)	0.005 (2)	−0.001 (2)
C13	0.016 (2)	0.054 (3)	0.024 (3)	0.004 (2)	−0.0021 (18)	0.005 (2)
C21	0.035 (3)	0.020 (2)	0.040 (3)	0.000 (2)	0.013 (2)	0.004 (2)
C22	0.022 (2)	0.030 (3)	0.024 (2)	−0.0022 (19)	0.0028 (18)	0.0088 (19)
C31	0.038 (3)	0.020 (2)	0.024 (2)	0.005 (2)	0.000 (2)	−0.0015 (19)
C32	0.022 (2)	0.021 (2)	0.029 (3)	−0.0071 (18)	−0.0001 (18)	−0.0045 (19)
C41	0.018 (2)	0.034 (3)	0.018 (2)	0.0044 (18)	0.0076 (17)	−0.0010 (19)
C42	0.021 (2)	0.026 (2)	0.034 (3)	−0.0043 (19)	0.0071 (19)	0.000 (2)
C43	0.015 (2)	0.029 (2)	0.020 (2)	0.0030 (17)	0.0015 (16)	0.0008 (18)
C51	0.028 (2)	0.018 (2)	0.032 (3)	0.0104 (18)	0.0026 (19)	0.0014 (19)
C52	0.028 (2)	0.023 (2)	0.019 (2)	0.0035 (18)	0.0031 (18)	0.0076 (18)
C61	0.036 (3)	0.014 (2)	0.020 (2)	0.0036 (18)	0.0020 (19)	−0.0031 (17)
C62	0.024 (2)	0.022 (2)	0.036 (3)	−0.0046 (18)	0.003 (2)	−0.009 (2)
Au2	0.01443 (11)	0.02328 (12)	0.01210 (11)	0.00557 (9)	0.00144 (8)	−0.00054 (9)
Br1	0.0228 (2)	0.0343 (2)	0.0235 (2)	−0.00354 (19)	0.00076 (17)	0.00478 (19)
Br2	0.0216 (2)	0.0346 (2)	0.0176 (2)	0.00501 (18)	−0.00265 (16)	0.00469 (18)
Au3	0.01867 (11)	0.01204 (10)	0.01515 (11)	−0.00082 (8)	0.00087 (8)	−0.00027 (8)
Br3	0.0242 (2)	0.0172 (2)	0.0293 (2)	0.00082 (17)	−0.00192 (17)	0.00480 (18)
Br4	0.0218 (2)	0.0187 (2)	0.0248 (2)	−0.00377 (16)	0.00270 (16)	0.00122 (17)

### Bis[tert-butylbis(propan-2-yl)-λ5-phosphaneselanone-κSe]gold(I) tetrabromidoaurate(III) (4b) Geometric parameters (Å, º)

**Table:**

Au1—Se1	2.4017 (4)	C21—H21C	0.9800
Au1—Se2	2.4057 (4)	C22—H22A	0.9800
Se1—P1	2.1929 (10)	C22—H22B	0.9800
Se2—P2	2.1864 (10)	C22—H22C	0.9800
P1—C3	1.843 (4)	C31—H31A	0.9800
P1—C2	1.845 (4)	C31—H31B	0.9800
P1—C1	1.873 (4)	C31—H31C	0.9800
P2—C5	1.840 (4)	C32—H32A	0.9800
P2—C6	1.847 (4)	C32—H32B	0.9800
P2—C4	1.866 (4)	C32—H32C	0.9800
C1—C12	1.530 (6)	C41—H41A	0.9800
C1—C13	1.538 (6)	C41—H41B	0.9800
C1—C11	1.552 (6)	C41—H41C	0.9800
C2—C21	1.528 (6)	C42—H42A	0.9800
C2—C22	1.539 (6)	C42—H42B	0.9800
C2—H2	1.0000	C42—H42C	0.9800
C3—C32	1.530 (5)	C43—H43A	0.9800
C3—C31	1.535 (5)	C43—H43B	0.9800
C3—H3	1.0000	C43—H43C	0.9800
C4—C41	1.538 (5)	C51—H51A	0.9800
C4—C42	1.541 (6)	C51—H51B	0.9800
C4—C43	1.544 (5)	C51—H51C	0.9800
C5—C51	1.528 (5)	C52—H52A	0.9800
C5—C52	1.535 (5)	C52—H52B	0.9800
C5—H5	1.0000	C52—H52C	0.9800
C6—C61	1.519 (5)	C61—H61A	0.9800
C6—C62	1.534 (6)	C61—H61B	0.9800
C6—H6	1.0000	C61—H61C	0.9800
C11—H11A	0.9800	C62—H62A	0.9800
C11—H11B	0.9800	C62—H62B	0.9800
C11—H11C	0.9800	C62—H62C	0.9800
C12—H12A	0.9800	Au2—Br2	2.4254 (4)
C12—H12B	0.9800	Au2—Br2^i^	2.4254 (4)
C12—H12C	0.9800	Au2—Br1	2.4337 (4)
C13—H13A	0.9800	Au2—Br1^i^	2.4337 (4)
C13—H13B	0.9800	Au3—Br3^ii^	2.4285 (4)
C13—H13C	0.9800	Au3—Br3	2.4285 (4)
C21—H21A	0.9800	Au3—Br4^ii^	2.4320 (4)
C21—H21B	0.9800	Au3—Br4	2.4320 (4)
			
Se1—Au1—Se2	176.734 (16)	H21B—C21—H21C	109.5
P1—Se1—Au1	98.27 (3)	C2—C22—H22A	109.5
P2—Se2—Au1	100.69 (3)	C2—C22—H22B	109.5
C3—P1—C2	105.40 (18)	H22A—C22—H22B	109.5
C3—P1—C1	108.68 (19)	C2—C22—H22C	109.5
C2—P1—C1	114.03 (19)	H22A—C22—H22C	109.5
C3—P1—Se1	111.62 (13)	H22B—C22—H22C	109.5
C2—P1—Se1	110.54 (13)	C3—C31—H31A	109.5
C1—P1—Se1	106.65 (14)	C3—C31—H31B	109.5
C5—P2—C6	105.53 (18)	H31A—C31—H31B	109.5
C5—P2—C4	113.97 (18)	C3—C31—H31C	109.5
C6—P2—C4	109.25 (18)	H31A—C31—H31C	109.5
C5—P2—Se2	110.39 (13)	H31B—C31—H31C	109.5
C6—P2—Se2	111.16 (13)	C3—C32—H32A	109.5
C4—P2—Se2	106.60 (13)	C3—C32—H32B	109.5
C12—C1—C13	108.5 (4)	H32A—C32—H32B	109.5
C12—C1—C11	109.4 (4)	C3—C32—H32C	109.5
C13—C1—C11	109.5 (4)	H32A—C32—H32C	109.5
C12—C1—P1	108.8 (3)	H32B—C32—H32C	109.5
C13—C1—P1	110.7 (3)	C4—C41—H41A	109.5
C11—C1—P1	109.9 (3)	C4—C41—H41B	109.5
C21—C2—C22	111.7 (3)	H41A—C41—H41B	109.5
C21—C2—P1	115.3 (3)	C4—C41—H41C	109.5
C22—C2—P1	113.0 (3)	H41A—C41—H41C	109.5
C21—C2—H2	105.2	H41B—C41—H41C	109.5
C22—C2—H2	105.2	C4—C42—H42A	109.5
P1—C2—H2	105.2	C4—C42—H42B	109.5
C32—C3—C31	109.3 (3)	H42A—C42—H42B	109.5
C32—C3—P1	112.6 (3)	C4—C42—H42C	109.5
C31—C3—P1	112.1 (3)	H42A—C42—H42C	109.5
C32—C3—H3	107.6	H42B—C42—H42C	109.5
C31—C3—H3	107.6	C4—C43—H43A	109.5
P1—C3—H3	107.6	C4—C43—H43B	109.5
C41—C4—C42	109.1 (3)	H43A—C43—H43B	109.5
C41—C4—C43	110.6 (3)	C4—C43—H43C	109.5
C42—C4—C43	107.2 (3)	H43A—C43—H43C	109.5
C41—C4—P2	110.1 (3)	H43B—C43—H43C	109.5
C42—C4—P2	109.2 (3)	C5—C51—H51A	109.5
C43—C4—P2	110.6 (3)	C5—C51—H51B	109.5
C51—C5—C52	111.3 (3)	H51A—C51—H51B	109.5
C51—C5—P2	115.8 (3)	C5—C51—H51C	109.5
C52—C5—P2	113.6 (3)	H51A—C51—H51C	109.5
C51—C5—H5	105.0	H51B—C51—H51C	109.5
C52—C5—H5	105.0	C5—C52—H52A	109.5
P2—C5—H5	105.0	C5—C52—H52B	109.5
C61—C6—C62	110.7 (3)	H52A—C52—H52B	109.5
C61—C6—P2	112.4 (3)	C5—C52—H52C	109.5
C62—C6—P2	111.9 (3)	H52A—C52—H52C	109.5
C61—C6—H6	107.2	H52B—C52—H52C	109.5
C62—C6—H6	107.2	C6—C61—H61A	109.5
P2—C6—H6	107.2	C6—C61—H61B	109.5
C1—C11—H11A	109.5	H61A—C61—H61B	109.5
C1—C11—H11B	109.5	C6—C61—H61C	109.5
H11A—C11—H11B	109.5	H61A—C61—H61C	109.5
C1—C11—H11C	109.5	H61B—C61—H61C	109.5
H11A—C11—H11C	109.5	C6—C62—H62A	109.5
H11B—C11—H11C	109.5	C6—C62—H62B	109.5
C1—C12—H12A	109.5	H62A—C62—H62B	109.5
C1—C12—H12B	109.5	C6—C62—H62C	109.5
H12A—C12—H12B	109.5	H62A—C62—H62C	109.5
C1—C12—H12C	109.5	H62B—C62—H62C	109.5
H12A—C12—H12C	109.5	Br2—Au2—Br2^i^	180.0
H12B—C12—H12C	109.5	Br2—Au2—Br1	90.581 (15)
C1—C13—H13A	109.5	Br2^i^—Au2—Br1	89.419 (15)
C1—C13—H13B	109.5	Br2—Au2—Br1^i^	89.419 (15)
H13A—C13—H13B	109.5	Br2^i^—Au2—Br1^i^	90.581 (15)
C1—C13—H13C	109.5	Br1—Au2—Br1^i^	180.0
H13A—C13—H13C	109.5	Br3^ii^—Au3—Br3	180.0
H13B—C13—H13C	109.5	Br3^ii^—Au3—Br4^ii^	90.810 (14)
C2—C21—H21A	109.5	Br3—Au3—Br4^ii^	89.190 (14)
C2—C21—H21B	109.5	Br3^ii^—Au3—Br4	89.191 (14)
H21A—C21—H21B	109.5	Br3—Au3—Br4	90.809 (14)
C2—C21—H21C	109.5	Br4^ii^—Au3—Br4	180.0
H21A—C21—H21C	109.5		
			
Au1—Se1—P1—C3	−68.24 (14)	C2—P1—C3—C31	−171.7 (3)
Au1—Se1—P1—C2	48.73 (14)	C1—P1—C3—C31	65.7 (3)
Au1—Se1—P1—C1	173.20 (15)	Se1—P1—C3—C31	−51.7 (3)
Au1—Se2—P2—C5	−39.44 (14)	C5—P2—C4—C41	77.4 (3)
Au1—Se2—P2—C6	77.32 (14)	C6—P2—C4—C41	−40.4 (3)
Au1—Se2—P2—C4	−163.72 (13)	Se2—P2—C4—C41	−160.6 (2)
C3—P1—C1—C12	157.2 (3)	C5—P2—C4—C42	−42.3 (3)
C2—P1—C1—C12	40.0 (4)	C6—P2—C4—C42	−160.1 (3)
Se1—P1—C1—C12	−82.3 (3)	Se2—P2—C4—C42	79.7 (3)
C3—P1—C1—C13	−83.6 (3)	C5—P2—C4—C43	−160.1 (3)
C2—P1—C1—C13	159.2 (3)	C6—P2—C4—C43	82.2 (3)
Se1—P1—C1—C13	36.9 (3)	Se2—P2—C4—C43	−38.1 (3)
C3—P1—C1—C11	37.4 (4)	C6—P2—C5—C51	−170.1 (3)
C2—P1—C1—C11	−79.8 (3)	C4—P2—C5—C51	70.0 (3)
Se1—P1—C1—C11	157.9 (3)	Se2—P2—C5—C51	−49.9 (3)
C3—P1—C2—C21	169.5 (3)	C6—P2—C5—C52	59.2 (3)
C1—P1—C2—C21	−71.4 (3)	C4—P2—C5—C52	−60.7 (3)
Se1—P1—C2—C21	48.7 (3)	Se2—P2—C5—C52	179.4 (3)
C3—P1—C2—C22	−60.3 (3)	C5—P2—C6—C61	174.2 (3)
C1—P1—C2—C22	58.8 (3)	C4—P2—C6—C61	−62.8 (3)
Se1—P1—C2—C22	178.9 (2)	Se2—P2—C6—C61	54.5 (3)
C2—P1—C3—C32	−48.0 (3)	C5—P2—C6—C62	48.9 (3)
C1—P1—C3—C32	−170.6 (3)	C4—P2—C6—C62	171.8 (3)
Se1—P1—C3—C32	72.0 (3)	Se2—P2—C6—C62	−70.8 (3)

Symmetry codes: (i) −*x*+1, −*y*, −*z*+1; (ii) −*x*+2, −*y*+1, −*z*+1.

### Bis[tert-butylbis(propan-2-yl)-λ5-phosphaneselanone-κSe]gold(I) tetrabromidoaurate(III) (4b) Hydrogen-bond geometry (Å, º)

**Table:**

*D*—H···*A*	*D*—H	H···*A*	*D*···*A*	*D*—H···*A*
C32—H32*C*···Au1	0.98	2.73	3.505 (4)	136
C13—H13*C*···Se1	0.98	2.80	3.305 (4)	113
C43—H43*B*···Se2	0.98	2.84	3.304 (4)	110
C3—H3···Br1^iii^	1.00	3.13	3.788 (4)	125
C52—H52*C*···Br1^i^	0.98	3.13	4.086 (4)	165
C5—H5···Br2	1.00	3.02	3.964 (4)	158
C62—H62*B*···Br2	0.98	3.04	3.802 (4)	136
C2—H2···Br3^iii^	1.00	3.20	3.990 (4)	137
C6—H6···Br3^iv^	1.00	3.02	3.870 (4)	144
C32—H32*B*···Br3^iii^	0.98	3.06	3.985 (4)	158
C42—H42*A*···Br3^v^	0.98	3.03	3.897 (4)	148
C62—H62*C*···Br4^iv^	0.98	3.10	4.018 (4)	158

Symmetry codes: (i) −*x*+1, −*y*, −*z*+1; (iii) −*x*+1, *y*−1/2, −*z*+1/2; (iv) −*x*+1, −*y*+1, −*z*+1; (v) *x*−1, *y*−1, *z*.

### Bis(tri-tert-butylphosphane sulfide)gold(I) tetrabromidoaurate(III) (5a) Crystal data

**Table:**

[Au(C_12_H_27_PS)_2_][AuBr_4_]	*Z* = 1
*M**_r_* = 1182.30	*F*(000) = 558
Triclinic, *P*1	*D*_x_ = 2.202 Mg m^−^^3^
*a* = 8.4858 (4) Å	Mo *K*α radiation, λ = 0.71073 Å
*b* = 9.3738 (4) Å	Cell parameters from 18685 reflections
*c* = 11.9910 (5) Å	θ = 2.3–30.7°
α = 105.533 (4)°	µ = 12.92 mm^−^^1^
β = 97.476 (4)°	*T* = 100 K
γ = 99.318 (4)°	Block, dichroic red / orange
*V* = 891.63 (7) Å^3^	0.12 × 0.12 × 0.08 mm

### Bis(tri-tert-butylphosphane sulfide)gold(I) tetrabromidoaurate(III) (5a) Data collection

**Table:**

Oxford Diffraction Xcalibur, Eos diffractometer	5273 independent reflections
Radiation source: fine-focus sealed tube	4754 reflections with *I* > 2σ(*I*)
Graphite monochromator	*R*_int_ = 0.035
Detector resolution: 16.1419 pixels mm^-1^	θ_max_ = 30.7°, θ_min_ = 2.3°
ω scan	*h* = −12→12
Absorption correction: multi-scan (CrysAlisPro; Rigaku OD, 2015)	*k* = −13→13
*T*_min_ = 0.765, *T*_max_ = 1.000	*l* = −17→17
47038 measured reflections	

### Bis(tri-tert-butylphosphane sulfide)gold(I) tetrabromidoaurate(III) (5a) Refinement

**Table:**

Refinement on *F*^2^	Secondary atom site location: difference Fourier map
Least-squares matrix: full	Hydrogen site location: inferred from neighbouring sites
*R*[*F*^2^ > 2σ(*F*^2^)] = 0.017	H-atom parameters constrained
*wR*(*F*^2^) = 0.033	*w* = 1/[σ^2^(*F*_o_^2^) + (0.0116*P*)^2^ + 0.584*P*] where *P* = (*F*_o_^2^ + 2*F*_c_^2^)/3
*S* = 1.06	(Δ/σ)_max_ = 0.003
5273 reflections	Δρ_max_ = 0.73 e Å^−^^3^
167 parameters	Δρ_min_ = −0.71 e Å^−^^3^
0 restraints	Extinction correction: *SHELXL2019/3* (Sheldrick, 2015), *F*_c_^*^ = *kF*_c_[1+0.001×*F*_c_^2^λ^3^/sin(2θ)]^-1/4^
Primary atom site location: structure-invariant direct methods	Extinction coefficient: 0.00106 (8)

### Bis(tri-tert-butylphosphane sulfide)gold(I) tetrabromidoaurate(III) (5a) Fractional atomic coordinates and isotropic or equivalent isotropic displacement parameters (Å^2^)

**Table:**

	*x*	*y*	*z*	*U*_iso_*/*U*_eq_	
Au1	0.500000	0.500000	0.500000	0.01767 (3)	
P1	0.23519 (6)	0.62570 (6)	0.31823 (4)	0.01195 (9)	
S1	0.32147 (7)	0.65725 (6)	0.49172 (4)	0.01907 (11)	
C1	0.1193 (3)	0.7846 (2)	0.32673 (19)	0.0179 (4)	
C2	0.0947 (2)	0.4340 (2)	0.25201 (18)	0.0161 (4)	
C3	0.4086 (2)	0.6476 (2)	0.23441 (18)	0.0148 (4)	
C11	0.0041 (3)	0.7651 (3)	0.2107 (2)	0.0229 (5)	
H11A	−0.046051	0.853518	0.218391	0.034*	
H11B	−0.080684	0.673822	0.193650	0.034*	
H11C	0.065876	0.755553	0.146441	0.034*	
C12	0.2391 (3)	0.9378 (2)	0.3589 (2)	0.0242 (5)	
H12A	0.294987	0.942582	0.293034	0.036*	
H12B	0.319085	0.948369	0.428893	0.036*	
H12C	0.179396	1.020062	0.375282	0.036*	
C13	0.0190 (3)	0.7956 (3)	0.4264 (2)	0.0255 (5)	
H13A	0.092498	0.822098	0.502309	0.038*	
H13B	−0.054057	0.697986	0.413248	0.038*	
H13C	−0.045101	0.873804	0.426810	0.038*	
C21	0.0490 (3)	0.3926 (3)	0.11710 (18)	0.0214 (4)	
H21A	−0.032059	0.297666	0.087433	0.032*	
H21B	0.146181	0.380317	0.082385	0.032*	
H21C	0.003949	0.473460	0.095605	0.032*	
C22	−0.0621 (3)	0.4321 (3)	0.3032 (2)	0.0249 (5)	
H22A	−0.124008	0.499729	0.275828	0.037*	
H22B	−0.034697	0.466349	0.389330	0.037*	
H22C	−0.127572	0.328918	0.276980	0.037*	
C23	0.1708 (3)	0.3088 (2)	0.2847 (2)	0.0214 (4)	
H23A	0.189478	0.328889	0.370358	0.032*	
H23B	0.274437	0.307373	0.257067	0.032*	
H23C	0.096941	0.210579	0.247410	0.032*	
C31	0.3558 (3)	0.6877 (3)	0.12054 (19)	0.0202 (4)	
H31A	0.446562	0.692699	0.077889	0.030*	
H31B	0.323728	0.785957	0.140472	0.030*	
H31C	0.263619	0.609826	0.070876	0.030*	
C32	0.5513 (3)	0.7717 (3)	0.3137 (2)	0.0215 (4)	
H32A	0.588570	0.743513	0.384194	0.032*	
H32B	0.515439	0.867910	0.337020	0.032*	
H32C	0.640651	0.782509	0.270440	0.032*	
C33	0.4739 (3)	0.5008 (2)	0.20084 (19)	0.0192 (4)	
H33A	0.567617	0.516960	0.162526	0.029*	
H33B	0.388545	0.419573	0.146635	0.029*	
H33C	0.506945	0.472478	0.271985	0.029*	
Au2	0.500000	1.000000	1.000000	0.01376 (3)	
Br1	0.43975 (3)	0.87358 (2)	0.78998 (2)	0.02087 (5)	
Br2	0.77780 (3)	0.96487 (3)	1.00751 (2)	0.02657 (5)	

### Bis(tri-tert-butylphosphane sulfide)gold(I) tetrabromidoaurate(III) (5a) Atomic displacement parameters (Å^2^)

**Table:**

	*U*^11^	*U*^22^	*U*^33^	*U*^12^	*U*^13^	*U*^23^
Au1	0.01701 (6)	0.02443 (6)	0.01215 (6)	0.00535 (4)	−0.00136 (4)	0.00762 (4)
P1	0.0115 (2)	0.0145 (2)	0.0100 (2)	0.00275 (18)	0.00132 (18)	0.00408 (19)
S1	0.0214 (3)	0.0257 (3)	0.0100 (2)	0.0085 (2)	0.00031 (19)	0.0041 (2)
C1	0.0179 (10)	0.0197 (10)	0.0175 (10)	0.0086 (8)	0.0036 (8)	0.0050 (8)
C2	0.0157 (10)	0.0168 (10)	0.0142 (10)	−0.0010 (8)	−0.0002 (8)	0.0061 (8)
C3	0.0147 (9)	0.0164 (9)	0.0151 (10)	0.0037 (7)	0.0056 (8)	0.0059 (8)
C11	0.0208 (11)	0.0294 (12)	0.0233 (11)	0.0117 (9)	0.0026 (9)	0.0124 (10)
C12	0.0279 (12)	0.0161 (10)	0.0292 (13)	0.0080 (9)	0.0060 (10)	0.0053 (9)
C13	0.0259 (12)	0.0316 (12)	0.0233 (12)	0.0148 (10)	0.0114 (10)	0.0069 (10)
C21	0.0235 (11)	0.0210 (11)	0.0153 (11)	−0.0003 (9)	−0.0047 (8)	0.0046 (9)
C22	0.0175 (11)	0.0293 (12)	0.0269 (12)	−0.0025 (9)	0.0050 (9)	0.0103 (10)
C23	0.0250 (11)	0.0170 (10)	0.0220 (11)	0.0009 (8)	0.0011 (9)	0.0087 (9)
C31	0.0222 (11)	0.0235 (11)	0.0197 (11)	0.0066 (9)	0.0082 (9)	0.0111 (9)
C32	0.0137 (10)	0.0237 (11)	0.0265 (12)	0.0013 (8)	0.0027 (9)	0.0086 (9)
C33	0.0190 (10)	0.0234 (11)	0.0182 (11)	0.0089 (8)	0.0060 (8)	0.0070 (9)
Au2	0.01409 (5)	0.00987 (5)	0.01663 (6)	0.00063 (4)	0.00114 (4)	0.00460 (4)
Br1	0.02326 (11)	0.01784 (10)	0.01773 (11)	−0.00018 (8)	0.00122 (8)	0.00270 (8)
Br2	0.01682 (10)	0.02703 (12)	0.03089 (13)	0.00700 (9)	0.00045 (9)	0.00063 (10)

### Bis(tri-tert-butylphosphane sulfide)gold(I) tetrabromidoaurate(III) (5a) Geometric parameters (Å, º)

**Table:**

Au1—S1^i^	2.2891 (5)	C13—H13C	0.9800
Au1—S1	2.2891 (5)	C21—H21A	0.9800
P1—C2	1.892 (2)	C21—H21B	0.9800
P1—C3	1.901 (2)	C21—H21C	0.9800
P1—C1	1.901 (2)	C22—H22A	0.9800
P1—S1	2.0384 (7)	C22—H22B	0.9800
C1—C12	1.540 (3)	C22—H22C	0.9800
C1—C11	1.541 (3)	C23—H23A	0.9800
C1—C13	1.546 (3)	C23—H23B	0.9800
C2—C22	1.535 (3)	C23—H23C	0.9800
C2—C21	1.539 (3)	C31—H31A	0.9800
C2—C23	1.540 (3)	C31—H31B	0.9800
C3—C33	1.539 (3)	C31—H31C	0.9800
C3—C31	1.541 (3)	C32—H32A	0.9800
C3—C32	1.541 (3)	C32—H32B	0.9800
C11—H11A	0.9800	C32—H32C	0.9800
C11—H11B	0.9800	C33—H33A	0.9800
C11—H11C	0.9800	C33—H33B	0.9800
C12—H12A	0.9800	C33—H33C	0.9800
C12—H12B	0.9800	Au2—Br1	2.4245 (2)
C12—H12C	0.9800	Au2—Br1^ii^	2.4245 (2)
C13—H13A	0.9800	Au2—Br2^ii^	2.4260 (2)
C13—H13B	0.9800	Au2—Br2	2.4260 (2)
			
S1^i^—Au1—S1	180.0	H13B—C13—H13C	109.5
C2—P1—C3	111.22 (9)	C2—C21—H21A	109.5
C2—P1—C1	111.27 (10)	C2—C21—H21B	109.5
C3—P1—C1	111.39 (9)	H21A—C21—H21B	109.5
C2—P1—S1	110.08 (7)	C2—C21—H21C	109.5
C3—P1—S1	110.94 (7)	H21A—C21—H21C	109.5
C1—P1—S1	101.58 (7)	H21B—C21—H21C	109.5
P1—S1—Au1	107.15 (3)	C2—C22—H22A	109.5
C12—C1—C11	108.38 (18)	C2—C22—H22B	109.5
C12—C1—C13	106.10 (18)	H22A—C22—H22B	109.5
C11—C1—C13	108.66 (18)	C2—C22—H22C	109.5
C12—C1—P1	110.09 (14)	H22A—C22—H22C	109.5
C11—C1—P1	113.07 (15)	H22B—C22—H22C	109.5
C13—C1—P1	110.28 (14)	C2—C23—H23A	109.5
C22—C2—C21	108.46 (18)	C2—C23—H23B	109.5
C22—C2—C23	105.66 (17)	H23A—C23—H23B	109.5
C21—C2—C23	109.05 (17)	C2—C23—H23C	109.5
C22—C2—P1	109.93 (15)	H23A—C23—H23C	109.5
C21—C2—P1	112.27 (14)	H23B—C23—H23C	109.5
C23—C2—P1	111.23 (14)	C3—C31—H31A	109.5
C33—C3—C31	108.08 (17)	C3—C31—H31B	109.5
C33—C3—C32	106.56 (17)	H31A—C31—H31B	109.5
C31—C3—C32	109.51 (16)	C3—C31—H31C	109.5
C33—C3—P1	110.98 (14)	H31A—C31—H31C	109.5
C31—C3—P1	111.94 (14)	H31B—C31—H31C	109.5
C32—C3—P1	109.63 (14)	C3—C32—H32A	109.5
C1—C11—H11A	109.5	C3—C32—H32B	109.5
C1—C11—H11B	109.5	H32A—C32—H32B	109.5
H11A—C11—H11B	109.5	C3—C32—H32C	109.5
C1—C11—H11C	109.5	H32A—C32—H32C	109.5
H11A—C11—H11C	109.5	H32B—C32—H32C	109.5
H11B—C11—H11C	109.5	C3—C33—H33A	109.5
C1—C12—H12A	109.5	C3—C33—H33B	109.5
C1—C12—H12B	109.5	H33A—C33—H33B	109.5
H12A—C12—H12B	109.5	C3—C33—H33C	109.5
C1—C12—H12C	109.5	H33A—C33—H33C	109.5
H12A—C12—H12C	109.5	H33B—C33—H33C	109.5
H12B—C12—H12C	109.5	Br1—Au2—Br1^ii^	180.0
C1—C13—H13A	109.5	Br1—Au2—Br2^ii^	89.687 (9)
C1—C13—H13B	109.5	Br1^ii^—Au2—Br2^ii^	90.313 (9)
H13A—C13—H13B	109.5	Br1—Au2—Br2	90.312 (9)
C1—C13—H13C	109.5	Br1^ii^—Au2—Br2	89.687 (9)
H13A—C13—H13C	109.5	Br2^ii^—Au2—Br2	180.0
			
C2—P1—S1—Au1	71.01 (7)	C3—P1—C2—C21	−46.85 (17)
C3—P1—S1—Au1	−52.53 (7)	C1—P1—C2—C21	77.96 (17)
C1—P1—S1—Au1	−171.01 (7)	S1—P1—C2—C21	−170.22 (13)
C2—P1—C1—C12	−169.51 (14)	C3—P1—C2—C23	75.67 (16)
C3—P1—C1—C12	−44.79 (17)	C1—P1—C2—C23	−159.52 (14)
S1—P1—C1—C12	73.37 (15)	S1—P1—C2—C23	−47.70 (16)
C2—P1—C1—C11	−48.13 (18)	C2—P1—C3—C33	−40.99 (17)
C3—P1—C1—C11	76.59 (17)	C1—P1—C3—C33	−165.73 (14)
S1—P1—C1—C11	−165.24 (14)	S1—P1—C3—C33	81.89 (15)
C2—P1—C1—C13	73.75 (17)	C2—P1—C3—C31	79.86 (16)
C3—P1—C1—C13	−161.53 (15)	C1—P1—C3—C31	−44.89 (17)
S1—P1—C1—C13	−43.37 (16)	S1—P1—C3—C31	−157.27 (13)
C3—P1—C2—C22	−167.67 (14)	C2—P1—C3—C32	−158.42 (13)
C1—P1—C2—C22	−42.86 (17)	C1—P1—C3—C32	76.83 (15)
S1—P1—C2—C22	68.96 (15)	S1—P1—C3—C32	−35.55 (15)

Symmetry codes: (i) −*x*+1, −*y*+1, −*z*+1; (ii) −*x*+1, −*y*+2, −*z*+2.

### Bis(tri-tert-butylphosphane sulfide)gold(I) tetrabromidoaurate(III) (5a) Hydrogen-bond geometry (Å, º)

**Table:**

*D*—H···*A*	*D*—H	H···*A*	*D*···*A*	*D*—H···*A*
C33—H33*C*···Au1	0.98	2.69	3.567 (2)	150
C23—H23*A*···Au1	0.98	2.86	3.421 (2)	118
C22—H22*C*···Br1^iii^	0.98	2.88	3.756 (2)	150
C12—H12*A*···Br1^iv^	0.98	3.05	3.890 (2)	145
C32—H32*A*···S1	0.98	2.86	3.338 (2)	111
C23—H23*A*···S1	0.98	2.98	3.456 (2)	111
C13—H13*A*···S1	0.98	2.67	3.160 (2)	112

Symmetry codes: (iii) −*x*, −*y*+1, −*z*+1; (iv) −*x*+1, −*y*+2, −*z*+1.

### Bis(tri-tert-butylphosphane selenide-κSe)gold(I) tetrabromidoaurate(III) (5b) Crystal data

**Table:**

[Au(C_12_H_27_PSe)_2_][AuBr_4_]	*Z* = 1
*M**_r_* = 1276.10	*F*(000) = 594
Triclinic, *P*1	*D*_x_ = 2.330 Mg m^−^^3^
*a* = 8.4403 (4) Å	Mo *K*α radiation, λ = 0.71073 Å
*b* = 9.2135 (4) Å	Cell parameters from 14356 reflections
*c* = 12.6496 (5) Å	θ = 2.3–30.8°
α = 106.172 (4)°	µ = 14.56 mm^−^^1^
β = 101.100 (4)°	*T* = 100 K
γ = 97.485 (4)°	Block, dichroic red / orange
*V* = 909.28 (7) Å^3^	0.12 × 0.12 × 0.04 mm

### Bis(tri-tert-butylphosphane selenide-κSe)gold(I) tetrabromidoaurate(III) (5b) Data collection

**Table:**

Oxford Diffraction Xcalibur, Eos diffractometer	5398 independent reflections
Radiation source: fine-focus sealed tube	4704 reflections with *I* > 2σ(*I*)
Graphite monochromator	*R*_int_ = 0.039
Detector resolution: 16.1419 pixels mm^-1^	θ_max_ = 30.9°, θ_min_ = 2.4°
ω scan	*h* = −12→12
Absorption correction: multi-scan (CrysAlisPro; Rigaku OD, 2015)	*k* = −13→13
*T*_min_ = 0.468, *T*_max_ = 1.000	*l* = −18→18
48315 measured reflections	

### Bis(tri-tert-butylphosphane selenide-κSe)gold(I) tetrabromidoaurate(III) (5b) Refinement

**Table:**

Refinement on *F*^2^	Primary atom site location: structure-invariant direct methods
Least-squares matrix: full	Secondary atom site location: difference Fourier map
*R*[*F*^2^ > 2σ(*F*^2^)] = 0.020	Hydrogen site location: inferred from neighbouring sites
*wR*(*F*^2^) = 0.036	H-atom parameters constrained
*S* = 1.06	*w* = 1/[σ^2^(*F*_o_^2^) + (0.013*P*)^2^ + 0.5218*P*] where *P* = (*F*_o_^2^ + 2*F*_c_^2^)/3
5398 reflections	(Δ/σ)_max_ = 0.001
166 parameters	Δρ_max_ = 0.83 e Å^−^^3^
0 restraints	Δρ_min_ = −0.87 e Å^−^^3^

### Bis(tri-tert-butylphosphane selenide-κSe)gold(I) tetrabromidoaurate(III) (5b) Fractional atomic coordinates and isotropic or equivalent isotropic displacement parameters (Å^2^)

**Table:**

	*x*	*y*	*z*	*U*_iso_*/*U*_eq_	
Au1	0.500000	0.500000	0.500000	0.01861 (4)	
P1	0.23823 (7)	0.64599 (7)	0.31359 (5)	0.01078 (12)	
Se1	0.33995 (3)	0.69690 (3)	0.49665 (2)	0.01875 (6)	
C1	0.1452 (3)	0.8210 (3)	0.3065 (2)	0.0149 (5)	
C2	0.0740 (3)	0.4642 (3)	0.2605 (2)	0.0157 (5)	
C3	0.4077 (3)	0.6294 (3)	0.2335 (2)	0.0149 (5)	
C11	0.0226 (3)	0.7960 (3)	0.1928 (2)	0.0187 (5)	
H11A	−0.012248	0.893101	0.191675	0.028*	
H11B	−0.073718	0.717803	0.183909	0.028*	
H11C	0.075797	0.761303	0.130341	0.028*	
C12	0.2828 (3)	0.9601 (3)	0.3281 (2)	0.0211 (6)	
H12A	0.335033	0.943442	0.264003	0.032*	
H12B	0.365231	0.972203	0.397672	0.032*	
H12C	0.236033	1.053424	0.336352	0.032*	
C13	0.0553 (3)	0.8691 (3)	0.4026 (2)	0.0236 (6)	
H13A	0.133672	0.894224	0.476149	0.035*	
H13B	−0.033236	0.783986	0.394871	0.035*	
H13C	0.008642	0.959636	0.397541	0.035*	
C21	0.0156 (3)	0.4067 (3)	0.1300 (2)	0.0240 (6)	
H21A	−0.077729	0.320043	0.106641	0.036*	
H21B	0.105937	0.373030	0.097288	0.036*	
H21C	−0.018463	0.490517	0.103187	0.036*	
C22	−0.0744 (3)	0.4956 (3)	0.3114 (3)	0.0247 (6)	
H22A	−0.125847	0.570296	0.281828	0.037*	
H22B	−0.037130	0.537331	0.394132	0.037*	
H22C	−0.154595	0.399274	0.290525	0.037*	
C23	0.1345 (3)	0.3327 (3)	0.2995 (2)	0.0213 (6)	
H23A	0.165564	0.364689	0.382498	0.032*	
H23B	0.230240	0.308679	0.269764	0.032*	
H23C	0.046253	0.241134	0.270923	0.032*	
C31	0.3563 (3)	0.6577 (3)	0.1182 (2)	0.0222 (6)	
H31A	0.445177	0.645911	0.078661	0.033*	
H31B	0.334466	0.762354	0.130622	0.033*	
H31C	0.256479	0.582947	0.072048	0.033*	
C32	0.5661 (3)	0.7457 (3)	0.3056 (2)	0.0218 (6)	
H32A	0.603830	0.722334	0.376128	0.033*	
H32B	0.543344	0.850345	0.323377	0.033*	
H32C	0.651798	0.738455	0.262964	0.033*	
C33	0.4516 (3)	0.4686 (3)	0.2097 (2)	0.0196 (5)	
H33A	0.543109	0.465501	0.172319	0.029*	
H33B	0.355681	0.391299	0.160333	0.029*	
H33C	0.483986	0.446471	0.281408	0.029*	
Au2	0.500000	1.000000	1.000000	0.01304 (3)	
Br1	0.38051 (3)	0.89229 (3)	0.79758 (2)	0.02139 (6)	
Br2	0.77027 (3)	0.96169 (3)	0.97214 (2)	0.02058 (6)	

### Bis(tri-tert-butylphosphane selenide-κSe)gold(I) tetrabromidoaurate(III) (5b) Atomic displacement parameters (Å^2^)

**Table:**

	*U*^11^	*U*^22^	*U*^33^	*U*^12^	*U*^13^	*U*^23^
Au1	0.01911 (7)	0.02043 (7)	0.01488 (7)	0.00373 (5)	−0.00363 (5)	0.00853 (6)
P1	0.0104 (3)	0.0105 (3)	0.0104 (3)	0.0024 (2)	0.0000 (2)	0.0032 (2)
Se1	0.02330 (13)	0.01952 (13)	0.01099 (12)	0.00578 (10)	−0.00139 (10)	0.00389 (10)
C1	0.0162 (11)	0.0135 (11)	0.0157 (12)	0.0049 (9)	0.0032 (9)	0.0049 (10)
C2	0.0134 (11)	0.0126 (11)	0.0178 (13)	−0.0007 (9)	−0.0017 (9)	0.0047 (10)
C3	0.0126 (11)	0.0191 (12)	0.0160 (12)	0.0058 (9)	0.0051 (9)	0.0079 (10)
C11	0.0156 (12)	0.0219 (13)	0.0206 (14)	0.0086 (10)	0.0020 (10)	0.0093 (11)
C12	0.0228 (13)	0.0122 (12)	0.0286 (15)	0.0037 (10)	0.0052 (11)	0.0075 (11)
C13	0.0267 (14)	0.0236 (14)	0.0235 (15)	0.0123 (11)	0.0106 (11)	0.0054 (11)
C21	0.0220 (13)	0.0189 (13)	0.0215 (14)	−0.0001 (11)	−0.0079 (11)	0.0021 (11)
C22	0.0144 (12)	0.0246 (14)	0.0383 (17)	0.0014 (11)	0.0058 (11)	0.0160 (13)
C23	0.0212 (13)	0.0141 (12)	0.0279 (15)	0.0015 (10)	0.0005 (11)	0.0099 (11)
C31	0.0244 (14)	0.0291 (15)	0.0197 (14)	0.0122 (11)	0.0097 (11)	0.0118 (12)
C32	0.0121 (11)	0.0244 (14)	0.0314 (15)	0.0038 (10)	0.0044 (11)	0.0129 (12)
C33	0.0209 (13)	0.0219 (13)	0.0192 (13)	0.0118 (10)	0.0072 (10)	0.0065 (11)
Au2	0.01454 (6)	0.00968 (6)	0.01406 (7)	0.00039 (5)	0.00263 (5)	0.00384 (5)
Br1	0.02170 (13)	0.02263 (13)	0.01498 (13)	−0.00268 (10)	0.00182 (10)	0.00310 (10)
Br2	0.01669 (12)	0.02068 (13)	0.02315 (14)	0.00422 (10)	0.00508 (10)	0.00445 (10)

### Bis(tri-tert-butylphosphane selenide-κSe)gold(I) tetrabromidoaurate(III) (5b) Geometric parameters (Å, º)

**Table:**

Au1—Se1	2.4036 (3)	C13—H13C	0.9800
Au1—Se1^i^	2.4036 (3)	C21—H21A	0.9800
P1—C2	1.895 (2)	C21—H21B	0.9800
P1—C1	1.900 (2)	C21—H21C	0.9800
P1—C3	1.904 (2)	C22—H22A	0.9800
P1—Se1	2.2009 (6)	C22—H22B	0.9800
C1—C12	1.537 (3)	C22—H22C	0.9800
C1—C11	1.541 (3)	C23—H23A	0.9800
C1—C13	1.547 (3)	C23—H23B	0.9800
C2—C22	1.538 (4)	C23—H23C	0.9800
C2—C23	1.538 (3)	C31—H31A	0.9800
C2—C21	1.543 (4)	C31—H31B	0.9800
C3—C33	1.537 (3)	C31—H31C	0.9800
C3—C32	1.543 (3)	C32—H32A	0.9800
C3—C31	1.543 (3)	C32—H32B	0.9800
C11—H11A	0.9800	C32—H32C	0.9800
C11—H11B	0.9800	C33—H33A	0.9800
C11—H11C	0.9800	C33—H33B	0.9800
C12—H12A	0.9800	C33—H33C	0.9800
C12—H12B	0.9800	Au2—Br1^ii^	2.4265 (3)
C12—H12C	0.9800	Au2—Br1	2.4265 (3)
C13—H13A	0.9800	Au2—Br2	2.4295 (3)
C13—H13B	0.9800	Au2—Br2^ii^	2.4295 (3)
			
Se1—Au1—Se1^i^	180.0	H13B—C13—H13C	109.5
C2—P1—C1	111.15 (11)	C2—C21—H21A	109.5
C2—P1—C3	111.98 (11)	C2—C21—H21B	109.5
C1—P1—C3	110.57 (11)	H21A—C21—H21B	109.5
C2—P1—Se1	109.06 (8)	C2—C21—H21C	109.5
C1—P1—Se1	102.74 (8)	H21A—C21—H21C	109.5
C3—P1—Se1	110.96 (8)	H21B—C21—H21C	109.5
P1—Se1—Au1	101.806 (19)	C2—C22—H22A	109.5
C12—C1—C11	109.1 (2)	C2—C22—H22B	109.5
C12—C1—C13	105.1 (2)	H22A—C22—H22B	109.5
C11—C1—C13	108.1 (2)	C2—C22—H22C	109.5
C12—C1—P1	109.79 (17)	H22A—C22—H22C	109.5
C11—C1—P1	113.56 (17)	H22B—C22—H22C	109.5
C13—C1—P1	110.76 (17)	C2—C23—H23A	109.5
C22—C2—C23	106.1 (2)	C2—C23—H23B	109.5
C22—C2—C21	109.1 (2)	H23A—C23—H23B	109.5
C23—C2—C21	108.2 (2)	C2—C23—H23C	109.5
C22—C2—P1	109.27 (17)	H23A—C23—H23C	109.5
C23—C2—P1	111.81 (16)	H23B—C23—H23C	109.5
C21—C2—P1	112.14 (17)	C3—C31—H31A	109.5
C33—C3—C32	106.7 (2)	C3—C31—H31B	109.5
C33—C3—C31	107.3 (2)	H31A—C31—H31B	109.5
C32—C3—C31	109.5 (2)	C3—C31—H31C	109.5
C33—C3—P1	111.40 (17)	H31A—C31—H31C	109.5
C32—C3—P1	109.97 (17)	H31B—C31—H31C	109.5
C31—C3—P1	111.73 (17)	C3—C32—H32A	109.5
C1—C11—H11A	109.5	C3—C32—H32B	109.5
C1—C11—H11B	109.5	H32A—C32—H32B	109.5
H11A—C11—H11B	109.5	C3—C32—H32C	109.5
C1—C11—H11C	109.5	H32A—C32—H32C	109.5
H11A—C11—H11C	109.5	H32B—C32—H32C	109.5
H11B—C11—H11C	109.5	C3—C33—H33A	109.5
C1—C12—H12A	109.5	C3—C33—H33B	109.5
C1—C12—H12B	109.5	H33A—C33—H33B	109.5
H12A—C12—H12B	109.5	C3—C33—H33C	109.5
C1—C12—H12C	109.5	H33A—C33—H33C	109.5
H12A—C12—H12C	109.5	H33B—C33—H33C	109.5
H12B—C12—H12C	109.5	Br1^ii^—Au2—Br1	180.0
C1—C13—H13A	109.5	Br1^ii^—Au2—Br2	89.101 (10)
C1—C13—H13B	109.5	Br1—Au2—Br2	90.899 (10)
H13A—C13—H13B	109.5	Br1^ii^—Au2—Br2^ii^	90.898 (10)
C1—C13—H13C	109.5	Br1—Au2—Br2^ii^	89.102 (10)
H13A—C13—H13C	109.5	Br2—Au2—Br2^ii^	180.0
			
C2—P1—Se1—Au1	72.38 (9)	C1—P1—C2—C23	−161.96 (18)
C1—P1—Se1—Au1	−169.62 (8)	C3—P1—C2—C23	73.8 (2)
C3—P1—Se1—Au1	−51.43 (8)	Se1—P1—C2—C23	−49.39 (19)
C2—P1—C1—C12	−169.27 (17)	C1—P1—C2—C21	76.3 (2)
C3—P1—C1—C12	−44.3 (2)	C3—P1—C2—C21	−47.9 (2)
Se1—P1—C1—C12	74.22 (17)	Se1—P1—C2—C21	−171.13 (16)
C2—P1—C1—C11	−46.8 (2)	C2—P1—C3—C33	−39.8 (2)
C3—P1—C1—C11	78.21 (19)	C1—P1—C3—C33	−164.31 (17)
Se1—P1—C1—C11	−163.33 (16)	Se1—P1—C3—C33	82.36 (17)
C2—P1—C1—C13	75.1 (2)	C2—P1—C3—C32	−157.89 (17)
C3—P1—C1—C13	−159.92 (17)	C1—P1—C3—C32	77.56 (19)
Se1—P1—C1—C13	−41.45 (18)	Se1—P1—C3—C32	−35.76 (18)
C1—P1—C2—C22	−44.8 (2)	C2—P1—C3—C31	80.3 (2)
C3—P1—C2—C22	−168.98 (17)	C1—P1—C3—C31	−44.3 (2)
Se1—P1—C2—C22	67.81 (18)	Se1—P1—C3—C31	−157.59 (16)

Symmetry codes: (i) −*x*+1, −*y*+1, −*z*+1; (ii) −*x*+1, −*y*+2, −*z*+2.

### Bis(tri-tert-butylphosphane selenide-κSe)gold(I) tetrabromidoaurate(III) (5b) Hydrogen-bond geometry (Å, º)

**Table:**

*D*—H···*A*	*D*—H	H···*A*	*D*···*A*	*D*—H···*A*
C33—H33*C*···Au1	0.98	2.64	3.540 (3)	152
C23—H23*A*···Au1	0.98	2.86	3.449 (3)	120
C22—H22*C*···Br1^iii^	0.98	2.88	3.851 (3)	173
C12—H12*A*···Br1^iv^	0.98	3.02	3.793 (3)	137
C32—H32*A*···Se1	0.98	2.95	3.442 (3)	112
C23—H23*A*···Se1	0.98	3.03	3.556 (3)	115
C13—H13*A*···Se1	0.98	2.70	3.263 (3)	117

Symmetry codes: (iii) −*x*, −*y*+1, −*z*+1; (iv) −*x*+1, −*y*+2, −*z*+1.
